# Selective maternal seeding and environment shape the human gut microbiome

**DOI:** 10.1101/gr.233940.117

**Published:** 2018-04

**Authors:** Katri Korpela, Paul Costea, Luis Pedro Coelho, Stefanie Kandels-Lewis, Gonneke Willemsen, Dorret I. Boomsma, Nicola Segata, Peer Bork

**Affiliations:** 1 European Molecular Biology Laboratory, 69117 Heidelberg, Germany;; 2 Department of Bacteriology and Immunology, Immunobiology Research Program, University of Helsinki, 00014 Helsinki, Finland;; 3 Department of Biological Psychology, Vrije Universiteit, 1081 Amsterdam, The Netherlands;; 4 Centre for Integrative Biology, University of Trento, 38123 Trento, Italy;; 5 Max-Delbrück-Centre for Molecular Medicine, 13125 Berlin, Germany;; 6 Molecular Medicine Partnership Unit, 69120 Heidelberg, Germany;; 7 Department of Bioinformatics, Biocenter, University of Würzberg, 97024 Würzberg, Germany

## Abstract

Vertical transmission of bacteria from mother to infant at birth is postulated to initiate a life-long host-microbe symbiosis, playing an important role in early infant development. However, only the tracking of strictly defined unique microbial strains can clarify where the intestinal bacteria come from, how long the initial colonizers persist, and whether colonization by other strains from the environment can replace existing ones. Using rare single nucleotide variants in fecal metagenomes of infants and their family members, we show strong evidence of selective and persistent transmission of maternal strain populations to the vaginally born infant and their occasional replacement by strains from the environment, including those from family members, in later childhood. Only strains from the classes Actinobacteria and Bacteroidia, which are essential components of the infant microbiome, are transmitted from the mother and persist for at least 1 yr. In contrast, maternal strains of Clostridia, a dominant class in the mother's gut microbiome, are not observed in the infant. Caesarean-born infants show a striking lack of maternal transmission at birth. After the first year, strain influx from the family environment occurs and continues even in adulthood. Fathers appear to be more frequently donors of novel strains to other family members than receivers. Thus, the infant gut is seeded by selected maternal bacteria, which expand to form a stable community, with a rare but stable continuing strain influx over time.

The infant's intestinal microbiota are believed to guide the development of the host (Houghteling and Walker 2015 and references therein) and to be transmitted from the mother at birth (Dominguez-Bello et al. 2010; Cho and Blaser 2012; Funkhouser and Bordenstein 2013; Bäckhed et al. 2015). Transmission of bacteria is even suggested to represent a form of epigenetic inheritance (Gilbert 2014). The infant's immune system develops in tight interaction with the intestinal microbiome, which guides its maturation and may significantly influence the risk of immune-related diseases (Houghteling and Walker 2015). The early gut colonizers are therefore thought to play a fundamental role in the long-term health of the child (Houghteling and Walker 2015). However, despite the consensus view of maternal inheritance of the microbiome at birth, the source of the early colonizers has never been exhaustively studied. Targeted cultivation-based investigations have shown that mother-infant pairs often share strains of bifidobacteria and lactobacilli (Tannock et al. 1990; Matsumiya et al. 2002; Jiménez et al. 2008; Albesharat et al. 2011; Makino et al. 2013; Jost et al. 2014). Large scale 16S rRNA and metagenomic surveys have found that vaginally born infants often harbor species that can also be detected in the mother (Dominguez-Bello et al. 2010; Bäckhed et al. 2015), thus hinting at broad vertical transmission of the microbiota, but the same species can also be shared by unrelated individuals. Based on the taxonomic profile of the infant gut, it is clear that vaginal microbiota, consisting mostly of lactobacilli (Ravel et al. 2011), cannot account for the majority of infant gut species. The same is true for breast milk, as the overlap with infant gut taxa is minor (Asnicar et al. 2017). The most likely source of bacteria to the infant is the mother's gut, which contains most of the species present in the infant gut, albeit at different relative abundances (Bäckhed et al. 2015; Asnicar et al. 2017).

In order to track bacterial transmission events, high taxonomic resolution at the genomic level is required (Schloissnig et al. 2013; Zhu et al. 2015; Li et al. 2016; Nayfach et al. 2016; Truong et al. 2017). The nucleotide variation of bacterial genomes allows high-resolution fingerprinting of bacteria shared between individuals or within individuals over time. However, detecting the same single nucleotide variants (SNVs) in two samples is not evidence of transmission, as common SNVs (and common strains) can prevail in the population (Yassour et al. 2016). Only the detection of rare variants shared exclusively between two individuals assures true transmission. Recently, the ability of tracking maternal transmission and intra-individual stability of strain populations, measured as shared SNVs in metagenomics data, has been demonstrated (Nayfach et al. 2016; Yassour et al. 2016; Asnicar et al. 2017). As the infant's intestinal microbial composition changes considerably during the first year of life (Bäckhed et al. 2015) and beyond, the fate of maternal strains and the impact of family members or environment in general still remain to be uncovered. Recent data suggest that some postnatal colonization from the environment may occur (Yassour et al. 2016), but it is currently unknown if there is a restricted age window, during which colonization is possible. We here compiled a cohort of family members that allowed us to assess strain persistence and intra-family strain transmissions at birth and later in life, identifying strain transmission at different ages up to adulthood.

## Results

### Tracking strain sharing between metagenomes using rare marker SNVs

To identify the source and persistence of bacterial strains in children and their family members, we adapted genomic variation approaches (Schloissnig et al. 2013; Li et al. 2016) to monitor SNVs in fecal metagenomes from several European and North American cohorts, totaling 695 samples from 307 individuals in 159 families with children and adults of different ages, sampled at multiple time points (Supplemental Table S1). The cohorts included 139 infants, of whom 25 were born by Caesarean section (C-section). We collected publicly available metagenomic data on Swedish, Italian, and US mother-infant pairs (Bäckhed et al. 2015; Asnicar et al. 2017; Chu et al. 2017) and unrelated adults (The Human Microbiome Project Consortium 2012; Le Chatelier et al. 2013; Zeller et al. 2014) from the US, France, Denmark, and Spain and amended these data sets with newly generated data from 10 German and Dutch families (of European ethnicity) with children of various ages (Supplemental Table S2) to assess potential transmission between family members at different stages in life. In addition, we profiled publicly available data of 139 unrelated US adults, followed in time over 1 yr (The Human Microbiome Project Consortium 2012). The number of compared pairs of individuals by relation is presented in Supplemental Table S3. Although we pooled data from different cohorts, we did not find clear batch effects in the observed species composition (Supplemental Fig. S1). Rather, as expected, the species composition was strongly associated with age (Supplemental Fig. S1).

As it is currently not possible to fully resolve multiple individual bacterial strains using shotgun metagenomic data, we considered SNV species profiles to represent strain populations, whereby multiple strains of the same species may occur within individuals, some of which may be shared between individuals or at different time points. Given the complexity of SNV analysis in metagenomes and various sources of errors (Schloissnig et al. 2013; Li et al. 2016), we used a very conservative approach to define strain sharing. Namely, we only tracked rare marker SNVs (hereafter rmSNVs) that were not shared with any non-family-member to avoid analyzing uninformative common SNVs prevalent in the population. In addition to the analyzed families, we included 884 deeply sequenced metagenomes of Europeans and North Americans (The Human Microbiome Project Consortium 2012; Le Chatelier et al. 2013; Zeller et al. 2014) as a background population to exclude common variants that do not unambiguously ensure shared origin. For an illustration of the definition of rmSNVs, see Supplemental Figure S2. For each pairwise sample comparison (between any two samples A and B), we defined as the reference population a sample set that excluded all samples from the donors of samples A and B and from their family members. For sample A, we identified exclusive alleles (rmSNVs) that were not found in the reference population containing all samples from non-family-members of the compared pair of individuals. Each comparison had a different reference population and a different set of rmSNVs. As even in this restricted set, a small fraction of rmSNVs was shared by chance between pairs of unrelated individuals, i.e., from other families (mean similarity, 0.2%), we required >20% rmSNV sharing between compared samples as evidence of strain sharing (see Methods).

### Maternal transmission of strains and their persistence during infant development

**Figure 1.**
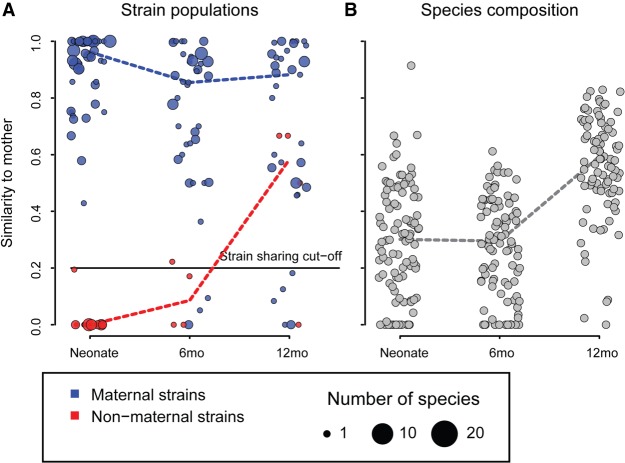
Similarity to mother over time of vaginally born infants (*n* = 114), measured as rmSNV similarity (*A*) and species composition similarity (*B*). Strains were classified as originally maternal if rmSNV similarity to mother was >0.2 (strain sharing cut-off) in the first neonatal sample available for each pair. Maternal/nonmaternal strains were summarized in each circle as median per individual. Even if the similarity to the mother increased in later time points, the original distinction of maternal/nonmaternal was kept for each child-mother pair through all further time points.

Thirty-four species had high genomic coverage (>10×) in at least two family members to allow rigorous statistical analysis (Supplemental Fig. S3). To track vertical strain transmission at birth in vaginally delivered infants in regard to those species, we compared the rmSNV profiles of the neonates to those of their mothers. In 87% of the 55 vaginally born neonates whose strain populations could be compared to those of their mothers, we found strong evidence of maternal transmission of bacteria (Fig. 1A), as indicated by high rmSNV similarity between neonates and their mothers (median 92%). While the total microbiota composition of these neonates did not resemble that of their mothers (mean correlation = 0.29 between mother-neonate pairs) (Fig. 1B), the similarity to the mother in composition increased with age as the child's microbiota matured (*P* < 0.0001) (Fig. 1B). As it was never significantly higher than the similarity to unrelated mothers, the increase in similarity to the mothers likely reflects the changes in the gut environment due to child development and diet on the microbiota composition.

**Figure 2.**
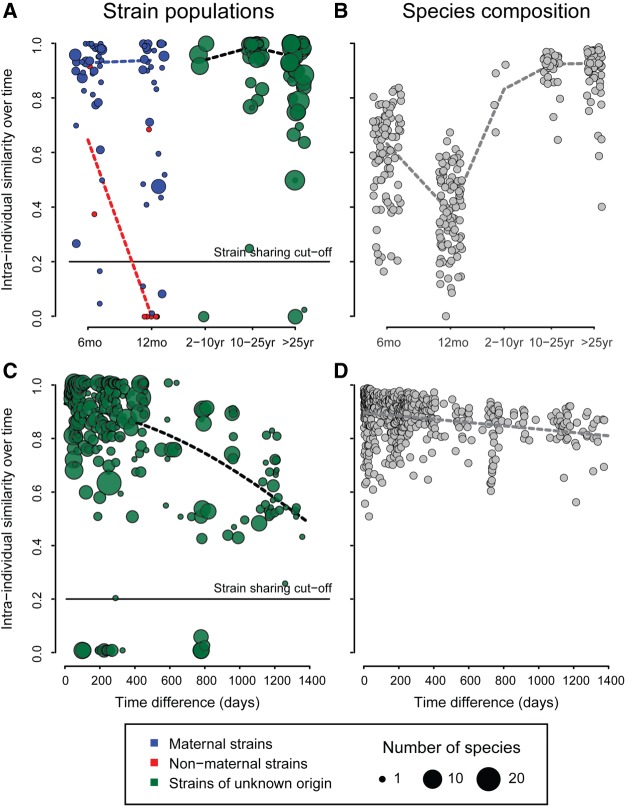
Intra-individual similarity over time. Panels *A* and *B* show similarity to previous time points at different ages (median over all time points per individual, including vaginally born infants and children, and adults irrespective of birth mode; *N* = 282). Panels *C* and *D* show intra-individual similarity by time difference between samples in >1-yr-olds (*N* = 304).

After establishing colonization with maternal strains, we studied strain persistence, tracking rmSNVs within individuals over time (Fig. 2). The rmSNV similarity between time points of the same individual was extremely high (median 90% similarity within a year) at all ages, even in infants (Fig. 2A), despite the considerable species-level compositional fluctuation observed there (Fig. 2B). This is consistent with generally high strain stability within individuals observed in previous studies (Schloissnig et al. 2013; Yassour et al. 2016). In infants, maternal strains were very stable over the first year of life, but the originally nonmaternal strains were nearly always replaced by the age of 12 mo, as indicated by close to zero intra-individual rmSNV similarity (Fig. 2A). In children and adults (*n* = 304), strain population similarity between time points declined gradually over time, with 13% rmSNV change per year on average (Fig. 2C), while the total species-level composition remained at close to 90% similarity over time spans of several years (Fig. 2D).

### Selective seeding by maternal Actinobacteria and Bacteroidia

As the maternally derived strains appeared to be more stable than the nonmaternal strains in the infants, we considered that different taxa might be differentially transmissible. Of the 34 species analyzed, 31 belong to only three bacterial classes, Actinobacteria (phylum Actinobacteria), Bacteroidia (phylum Bacteroidetes), and Clostridia (phylum Firmicutes), that together make up 90% of the assignable relative species abundance. We therefore focus on these three classes. Adults had a considerable abundance of Clostridia (mean relative abundance 34% of the total) and Bacteroidia (50%) and a low abundance of Actinobacteria (4%) (Supplemental Figs S1, S4). The microbiota of infants was dominated by Actinobacteria and Bacteroidia (together representing 59% of the total assignable abundance), with low abundance of Clostridia (8%).

**Figure 3.**
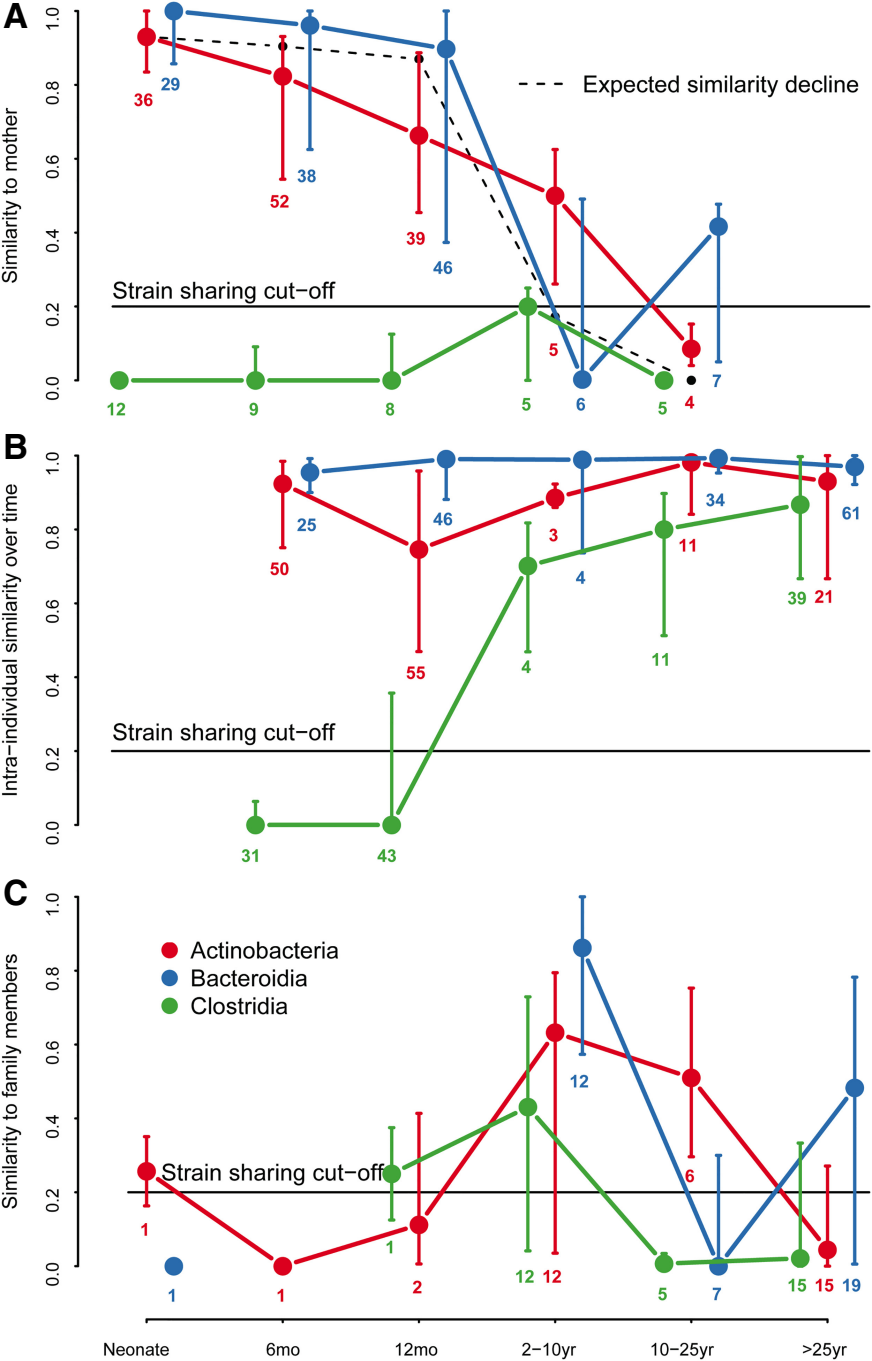
Phylogenetic signal in strain transmission and stability. (*A*) Strain population (rmSNV profile) similarity of vaginally born infants to mother. (*B*) Intra-individual rmSNV similarity over time in vaginally born infants and children, and adults regardless of birth mode. (*C*) Strain population (rmSNV) similarity to other family members. The symbols represent age group medians ± inter-quartile range per bacterial class. The number of individuals included in each group is indicated. Expected similarity decline is based on the intra-individual similarity decline in Figure 2C.

Distinct phylogenetic patterns were indeed evident in the transmissibility of strains from mother to neonate (Fig. 3A; Supplemental Table S4). Strain populations from Actinobacteria and Bacteroidia were nearly always shared between mother and vaginally born neonate: The strain populations in 97% of the neonates with tracked Actinobacteria (*n* = 36) and in 93% of those with tracked Bacteroidia species (*n* = 29) were identified as maternal (Fig. 3A), indicating transmission during birth (Supplemental Table S4). Maternal strains of Clostridia were never observed in any neonate with tracked Clostridia species (*n* = 12) in the first days of life (Fig. 3A; Supplemental Table S4), albeit the abundance of Clostridia in neonates was generally low (Supplemental Figs S1, S4). Of the few other species that did not belong to these three classes (Supplemental Fig. S5A; Supplemental Table S4), *Akkermansia muciniphila* (phylum Verrucomicrobia) and *Dialister invisus* (phylum Firmicutes) were not transmitted at birth, while *Collinsella aerofaciens* was (phylum Actinobacteria). Thus, although only about a half of the relative abundance of the mother's intestinal bacteria seem transmissible at birth (Actinobacteria and Bacteroidia together), the maternal strains of these two classes represent the vast majority of abundance in the neonate, with an expansion of environmental strains of Clostridia later in life.

Bacteroidia strain populations showed higher stability than Actinobacteria and Clostridia (*P* < 0.0001) (Fig. 3B), suggesting that they are less frequently replaced by new strains. Maternal Bacteroidia were still observable after 1 yr in 94% of the infants with maternal Bacteroidia strains (*n* = 46), compared to 81% of the infants retaining traceable maternal Actinobacteria strains (*n* = 37) (Fig. 3B). Diet changes did not influence the stability of maternal SNVs (*P* = 0.45) (Supplemental Fig. S6). All infants initially received breast milk (exclusively or supplemented with formula), and 14 infants later transitioned to formula feeding before solid foods by age 12 mo. After the age of 12 mo, rmSNV similarity to the mother gradually declined, roughly following the same time course as the decline in intra-individual rmSNV similarity in adults (Fig. 3A; Supplemental Fig. S5A).

Clostridia strains, as well as strains from *Dialister* and *Akkermansia*, appeared to colonize the infants persistently only after the first year, and even then, *Akkermansia* was unstable in a subset of the individuals (Fig. 3B; Supplemental Fig. S5B). The persistent colonization by these taxa coincided with a shift to a diet containing plant polysaccharides, the preferred substrate of Clostridia and *Dialister*, which likely enabled their stable colonization.

The transient nonmaternal strains appeared to be partly replaced by maternal ones during the first year (Fig. 1A), demonstrated by the high rmSNV similarity of the originally nonmaternally derived species to the maternal strains at 12 mo, implying postnatal colonization by strains from the environment. We therefore further investigated the potential influx of strains from the environment in later stages of life by also tracking rmSNVs between father-child, sibling, and spouse pairs and considering them as indicators of colonization by environmental strains.

### Strain transmission between family members in adulthood

**Figure 4.**
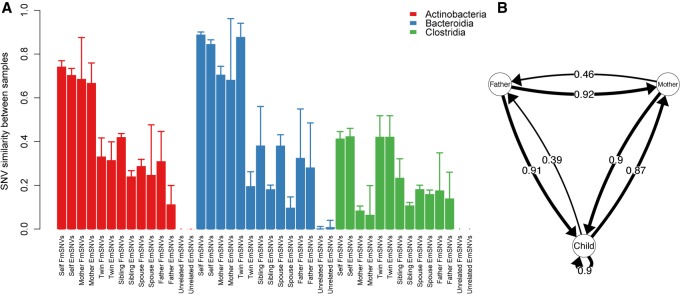
Strain similarity and transmissions within families. (*A*) The bars indicate mean ± SD. Two sets of rare marker SNVs were defined per pair of individuals: rare marker SNVs, which were allowed to be shared with other family members (marked here as Family marker SNVs, FmSNVs), and exclusive marker SNVs (EmSNVs), which were only shared between the compared pair. (*B*) The arrows indicate the cases where novel SNVs emerging in the focal individual were observed in previous time points of a given family member. The arrow from child to child indicates transmission between siblings. The numbers on the arrows represent the proportion of identified transmission events between the indicated pair of family members (number of observed transmission events/total number of family member comparisons).

The influence of the family environment peaked at age 2–10 yr (*n* = 12) (Fig. 3C; Supplemental Fig. S5C). After the age of 10 yr, strain sharing was generally low (*n* = 27) (Fig. 3C; Supplemental Fig. S5C), although still significantly more frequent than with unrelated individuals (*P* < 0.0001) (Fig. 4A), concordant with results from adult twins (Rothschild et al. 2017). Strain sharing between family members was stable over at least 1 mo, indicating that the shared strains were resident microbes. All family member pairs had similar levels of rmSNV sharing, apart from mother-child pairs, which shared more rmSNVs, perhaps a reflection of the maternal seeding. Twins generally did not have more similar rmSNV profiles than nontwin siblings (Fig. 4A). An exception was Clostridia, showing higher rmSNV similarity between twins than mother-child pairs, likely due to the lack of maternal seeding and the strong impact of shared environment for this class (Fig. 4A). In some cases, siblings had (nonsignificantly) higher rmSNV similarity than twins, which is likely due to chance. Overall, the results are in line with a recent study on adult twins in which monozygotic twins did not share more strains than dizygotic twins, indicating a lack of genetic influence on strain sharing (Xie et al. 2016; Rothschild et al. 2017).

Another indication for the retaining of maternal strains later in life is the exclusive sharing of many rmSNVs, i.e., the rmSNVs shared with mother were not observed in other family members, as indicated by the exclusively shared rmSNVs (EmSNVs) in Figure 4A. In mother-child pairs, the EmSNVs encompassed nearly all of the shared rmSNVs. This is in contrast to the shared rmSNVs between twins, siblings, father-child pairs, and spouses that were often also shared with a third family member (EmSNV sharing is often much lower than the total rmSNV sharing in Fig. 4A), implying a source in the shared household environment, with no evidence for direct transmission from a particular person.

To assess the directionality of strain transmissions within families, we tracked the emergence of novel SNVs that were not detected in previous samples of the focal individual, now extending the analysis from rmSNVs to all SNVs to increase statistical power. As the newly observed SNVs in one family member were indeed often present in previous samples of one or several family members, we assumed strain transmission between family members (Fig. 4B). Surprisingly, the father was significantly more often than the mother or the children the likely donor of intra-family transmissions, including transmissions between the parents and between parent and child (*P* < 0.0001), and less often the recipient (*P* < 0.0001). Thus, the fathers in the 10 families with at least two siblings analyzed appeared to bring most of the novel strains into the family environment.

### C-section prevents maternal seeding

As maternal selective seeding is likely to be affected by birth mode, we also analyzed 25 infants and six 2- to 10-yr-olds in our cohorts that were born by Caesarean section, which were excluded in the prior analyses. These children showed strikingly different strain transmission and persistence patterns compared to the vaginally born (Supplemental Figs S7, S8). The microbiome in the Caesarean-delivered infants was mostly devoid of the maternally transmitted seeding classes Actinobacteria and Bacteroidia during the first months; species of Bacteroidia in particular were consistently missing (Supplemental Fig. S7A), as observed in other cohorts (Bokulich et al. 2016; Yassour et al. 2016). Only six of the 15 Caesarean-delivered neonates had any species overlap with the mothers to allow SNV analysis, and we did not observe sharing of a single strain (Supplemental Fig. S7B), clearly implicating vaginal birth as the main transmission route. The likelihood of having maternal strains was 0.87 in the vaginally born neonates and 0 in the Caesarean-born (χ^2^ test, *P* < 0.0001). During their first year of life, the Caesarean-delivered infants gradually acquired maternal Actinobacteria and Bacteroidia strains, which replaced the original nonmaternal ones (Supplemental Fig. S7B). In addition to the absence of maternal strains, the Caesarean-delivered infants showed higher strain flux than the vaginally born ones, particularly regarding Bacteroidia strains (*P* < 0.0001) (Supplemental Fig. S8).

## Discussion

Taken together, our results indicate that maternal transmission of bacteria is a controlled process, in which only a minor, selected part of the maternal microbiome colonizes the neonate and expands to form the stable seeding community of the infant microbiome. The results demonstrate that colonization of the infant gut is a selective process, rather than randomly determined by the strains that the infant is exposed to. The selection most likely occurs in the infant gut after initial inoculation by maternal fecal microbes and may be due to the milk-based diet, which Actinobacteria and Bacteroidia species are able to utilize, thus gaining a selective advantage (Sela and Mills 2010; Marcobal et al. 2011). These taxa are considered important for healthy metabolic and immunological development in infants (Mazmanian et al. 2005; Korpela et al. 2017).

The high stability of the maternal strains in the infant gut demonstrates the importance of maternal seeding. It was recently suggested that certain species colonize the infant once and remain stable thereafter while others spread in the population, colonizing each individual several times (Yassour et al. 2016). Our results further this idea, identifying the maternally derived strains to be stable and the nonmaternal ones to be replaceable. Generally, the results may indicate that different symbiotic bacterial species have evolved different transmission strategies, some relying on vertical transmission at birth and others on horizontal transmission in later life. Horizontal transfer via the environment could be facilitated by endospore formation, which is common among Clostridia.

Surprisingly, infants and children are not more commonly colonized by novel strains than adults. After initial colonization, the maternal strains persist in infants. This implies that the transmitted part of the maternal gut microbiome may have a protective function, preventing the influx of environmental conspecific strains, which may have a higher risk of carrying unwanted properties. It is currently not clear why the maternal strains are more stable than nonmaternal conspecific strains. A candidate explanation is breast milk, which contains maternal immunoglobulins as well as oligosaccharide structures similar to those present in the maternal gut mucosa. Maternal bacterial strains may thus have an advantage as they are preselected to be compatible with these breast milk components. However, transitions to formula feeding and to solid foods during the first year did not reduce the stability of the maternal strains. Evidently the bacteria, especially Bacteroidia, were able to adapt to the new dietary pattern. Bacteroidia species are known to have a diverse repertoire of carbohydrate-active enzymes, which gives them considerable substrate flexibility (El Kaoutari et al. 2013).

Over the years, strain similarity to the mother declines, most likely due to gradual strain replacement, although mutations may contribute to this as well. Additionally, several species, including members of Clostridia and *Akkermansia muciniphila*, appear to colonize persistently only after the first year of life and are not derived from the mother. Such behavior of late-colonizers may be explained by their high degree of specialization to the conditions in the adult gut, rendering them unable to persistently colonize the neonate. Due to the frequent absence or very low abundance of Clostridia in the neonates, we cannot refute occasional transmission from the mother, but the consistent lack of maternal Clostridia rmSNVs in children of all ages, despite commonly observed paternal and fraternal strains, makes transmission at birth highly unlikely or at least extremely rare.

Our data show the dramatic effect of Caesarean section on infant gut colonization: These infants fail to receive maternal strains at birth and instead show a high degree of strain flux in early life, comparable to the flux observed in nontransmitted species in vaginally born infants. This suggests that there is initially a mismatch between the bacteria and the host, which is gradually resolved as the C-section-born infants acquire maternal strains postnatally from the environment. How the disruption of bacterial transmission affects the developing immune system is currently not known. Despite the low number of infants studied, these results do not support the generality of the recently suggested intra-uterine bacterial transmission via the placenta (Aagaard et al. 2014) or the entero-mammary route of transmission (Funkhouser and Bordenstein 2013; Jost et al. 2014; Rodriguez et al. 2014), as these should affect infants regardless of birth mode. It remains unclear whether this early fluctuation implies an increased risk of colonization by undesired strains with respective health consequences.

While the species composition of gut microbiota has been reported to be influenced by host genetics (Xie et al. 2016), our results demonstrate that the identity of the strains within a species is dependent on the environment (Rothschild et al. 2017). Family members are likely the most important environmental source of human gut microbes, and bacterial transmission between family members occurs also in later stages of life. The frequency, the constraints, and the functional consequences of strain transmission between family members and from other environmental sources still need to be investigated.

## Methods

### Data

We monitored bacterial strains in gut metagenomes of 100 Swedish mother-infant pairs, including 15 Caesarean-delivered infants (Bäckhed et al. 2015), 42 US infants—10 of which were Caesarean-delivered (Chu et al. 2017)—and five vaginally born Italian infants (Asnicar et al. 2017). The Swedish infants had been sampled during their first postnatal week and at ages 4 and 12 mo, the mothers only during the infant's first week. The US infants and mothers were sampled at birth and at 6 wk. The Italian infants and mothers were sampled at birth, at 7 mo, and at 1 yr. In addition, we sequenced metagenomes of eight German and two Dutch families with children of different ages, including two families with infants, two families with adult children, and five families with twins of different ages, some of which were Caesarean-delivered (Supplemental Table S2). As an additional adult cohort, we analyzed 139 female Human Microbiome Project participants sampled at one to three time points with 6–12 mo intervals. As the background population, we included 884 samples from different European cohorts.

We categorized the samples into six age groups: <1 wk (“Neonate,” *N* = 113), >1 wk to 6 mo (“6 months,” *N* = 139), 6 mo to 2 yr (“12 months,” *N* = 105), 2–10 yr (*N* = 12), 10–25 yr (*N* = 60), and >25 yr (*N* = 232).

### Sample collection, DNA extraction, and sequencing

Samples were collected fresh and immediately frozen at −20°C until arriving at the laboratory, where they were kept in long-term storage at −80°C. Genomic DNA was extracted from frozen fecal samples as previously described (Zeller et al. 2014) using a GNOME DNA Isolation Kit (MP Biomedicals). Libraries were generated and shotgun sequenced on the Illumina HiSeq 2000/2500 (Illumina) platform in a paired-end sequenced setup with 100-bp read lengths at the Genomics Core Facility, European Molecular Biology Laboratory.

### Species abundance estimation

All samples were processed with the same computational protocol. Reads were quality filtered and screened against the human genome sequence for removing contamination as previously described (Zeller et al. 2014). Sequencing reads were then mapped to a reference set consisting of 1753 genomes, each representative of one specI cluster (Mende et al. 2013) using MOCAT (Kultima et al. 2012) with default parameters. Specifically, reads were mapped at 97% identity, and multiple mappers were discarded. Computation of genome coverage for each specI cluster was performed using qaCompute (https://github.com/CosteaPaul/qaTools), resulting in estimations of both horizontal and vertical coverage per sample, per genome. The abundance of species was estimated based on the genome coverages and transformed into relative abundances. Species composition similarity between samples was calculated as the Pearson correlation of the log-transformed relative abundances.

### SNV sharing

Determination of SNVs was performed using the metaSNV tool (available at https://git.embl.de/costea/metaSNV) with default parameters. The number of SNVs varied by species, ranging from five to 116,915 (mean 22,308) for Clostridia, 3133–30,823 (mean 20,695) for Bacteroidetes, and 2–80,287 (mean 46,697) for Actinobacteria. Sharing of SNVs between samples was calculated as the number of shared SNVs divided by the number of positions that were detected in both samples. In cases where a species was not detected in a sample (genome coverage was 0), SNV sharing of that species between that sample and all other samples was set to 0.

To ensure that the shared SNVs were indeed of the same origin, the main part of the study was based on marker SNVs (rmSNVs) excluding all SNVs that were shared between unrelated individuals (apart from the pair of individuals being compared, even if they were unrelated), as these are uninformative for strain source tracking. The reference population thus contained all samples from individuals not related to the donors of the pair of samples (sample A and sample B) currently being compared. For sample A, we defined rmSNVs as the SNVs, which were not present in the reference population (keeping in mind that the reference population excludes sample B, even if the donor of sample B is not related to the donor of sample A). These rmSNVs were then compared to the SNVs in sample B. SNV sharing was only assessed if >100 marker-positions were available for the compared pair of samples.

We initially monitored the SNVs in 108 species but selected for detailed analysis a subset of 34 based on sufficient genome coverage (>40%). To make sure the observed inter-sample rmSNV similarity was not influenced by the observed number of rmSNVs in the samples or the abundance of the species, we checked for correlations between rmSNV sharing and rmSNV richness and species abundance. For the classes Actinobacteria, Bacteroidia, and Clostridia, the observed number of rmSNVs or the abundance of the species were not associated with rmSNV sharing. However, for other classes, the observed rmSNV similarity was not independent of rmSNV richness or species abundance, suggesting that, for many cases, the rmSNV similarity was underestimated due to insufficient sampling, despite the high sequencing depth. We therefore excluded these taxa from the analysis.

SNV frequencies within species often showed bimodal distributions, peaking at <20% or >80%. This indicates that individuals mostly harbored one dominant strain, either the one with the reference SNV or the one with the nonreference SNV. However, particularly among the infants, there were also many cases with unimodal or even distributions of SNV frequencies, indicating that having two or more strains simultaneously was common. Therefore, we considered the possibility that <100% SNV similarity could indicate the sharing of one strain, which coexisted with a nonshared strain. To establish a cut-off for reliably demonstrating strain sharing (with the possibility of coexisting nonshared strains), we assessed rmSNV similarities between unrelated individuals. Unrelated individuals rarely shared rmSNVs. The frequency of >20% similarity between unrelated pairs was 0.5% (Supplemental Fig. S9). We used this conservative cut-off for defining strain sharing: rmSNV similarity <20% was deemed insufficient evidence of strain sharing, resulting in 0.005 false discovery rate.

For more detailed analysis on intra-family strain sharing, we further tracked a subset of rmSNVs that were exclusively shared between the compared pair of individuals and with no other family members. We compared the family-specific and the exclusive rmSNV similarities within a pair of individuals to establish if the shared rmSNVs were likely a result of direct transmission (all shared rmSNVs were shared exclusively) or more likely came from a common source in the family environment (shared rmSNVs were also shared with other family members).

To establish directionality of strain transmission, we compared novel SNVs not seen in previous samples of the same individual to SNVs in previous samples of family members, including all SNVs to maximize the number of novel SNVs observed. Strain transmission from a family member was assumed to have occurred if novel SNVs appearing in an individual were observed in previous samples of a family member. Novel SNVs were rare, so novel rmSNVs were too rare for robust analysis. However, in a few individuals, we were able to trace novel rmSNVs to a family member, validating the concept.

The number of rmSNVs compared by species and type of relation between individuals is shown in Supplemental Figure S10.

### Statistical tests

Significance of group differences in SNV similarity (proportion of shared rmSNVs) was assessed using beta-regression (package betareg [Cribari-Neto and Zeileis 2010] in R [R Core Team 2015]). The significance of trends and group differences in compositional similarity, which was normally distributed, was assessed using linear models.

## Data access

The newly generated metagenomes from this study have been submitted to the European Nucleotide Archive (ENA; http://www.ebi.ac.uk/ena) under accession number PRJEB24041.

## Supplementary Material

Supplemental Material
