# Supplementary material for: Selective maternal seeding and environment shape the human gut microbiome
Source: Genome Res. 2018 Apr;28(4):561–8. doi: 10.1101/gr.233940.117 (PMC5880245; doi:10.1101/gr.233940.117)
Supplement: Supplemental Material [file supp_28_4_561__index.html]

Selective maternal seeding and environment shape the human gut microbiome — Supplemental Material 

# Selective maternal seeding and environment shape the human gut microbiome

## Supplemental Material

- Supplemental\_Fig\_S1.pdf
- Supplemental\_Fig\_S3.pdf
- Supplemental\_Fig\_S4.pdf
- Supplemental\_Fig\_S5.pdf
- Supplemental\_Fig\_S6.pdf
- Supplemental\_Fig\_S7.pdf
- Supplemental\_Fig\_S8.pdf
- Supplemental\_Fig\_S9.pdf
- Supplemental\_Fig\_S10.pdf
- Supplemental\_Table\_S1.docx
- Supplemental\_Table\_S2.docx
- Supplemental\_Table\_S3.docx
- Supplemental\_Table\_S4.docx
- Supplemental\_Fig\_S2.pdf
