# Supplementary material for: Selective maternal seeding and environment shape the human gut microbiome
Source: Genome Res. 2018 Apr;28(4):561–8. doi: 10.1101/gr.233940.117 (PMC5880245; doi:10.1101/gr.233940.117)
Supplement: Supplemental Material [file supp_gr.233940.117_Supplemental_Fig_S1.pdf]

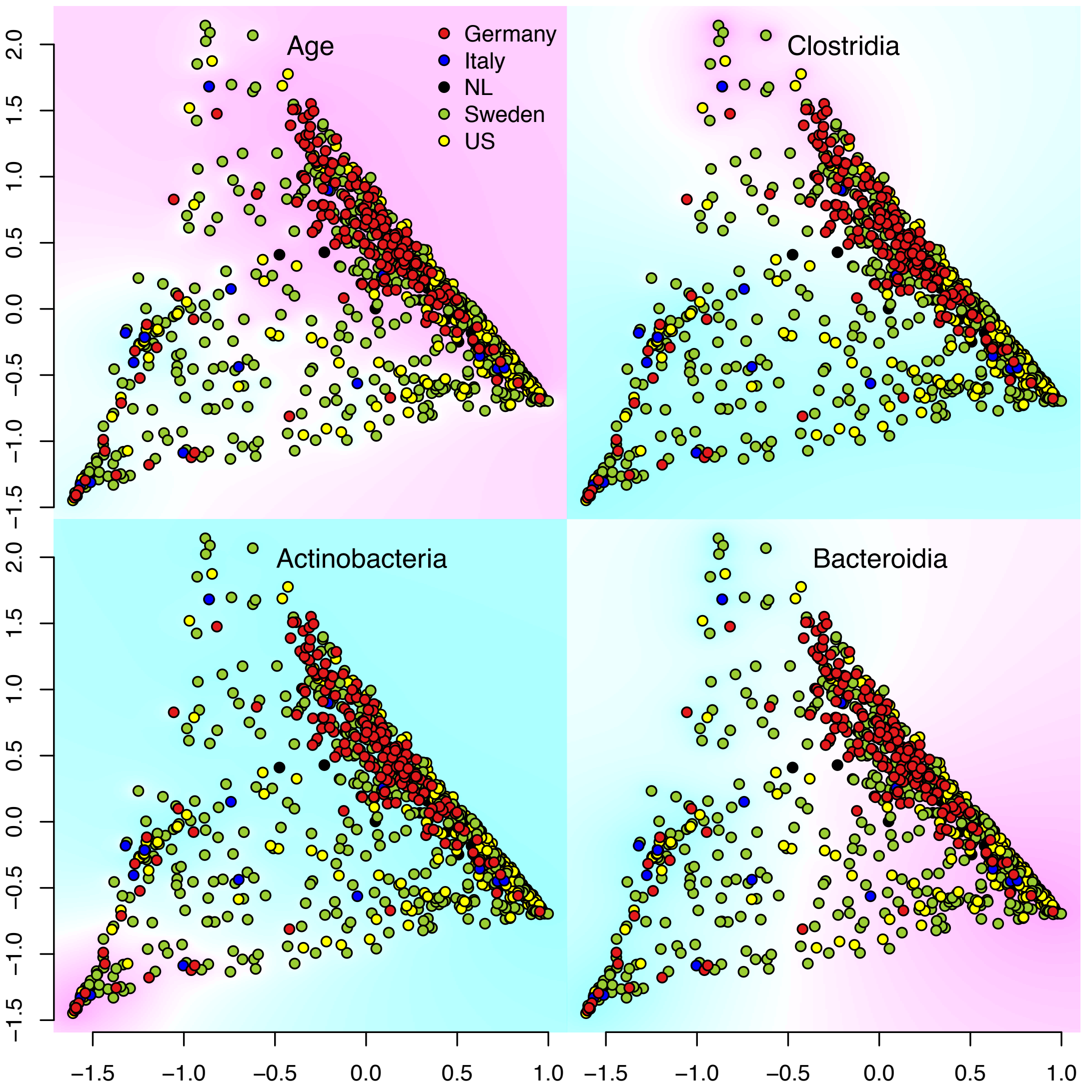

Supplemental Figure S1.
Taxonomic composition in the analysed cohorts. Each panel shows the first two principal coordinates based on Bray-Curtis distances, with the background on the panel coloured by distance-weighted interpolated values of age and the abundances of the three most abundant bacterial classes: light blue indicating low values and purple high values.
