# Supplementary material for: Selective maternal seeding and environment shape the human gut microbiome
Source: Genome Res. 2018 Apr;28(4):561–8. doi: 10.1101/gr.233940.117 (PMC5880245; doi:10.1101/gr.233940.117)
Supplement: Supplemental Material [file supp_gr.233940.117_Supplemental_Fig_S3.pdf]

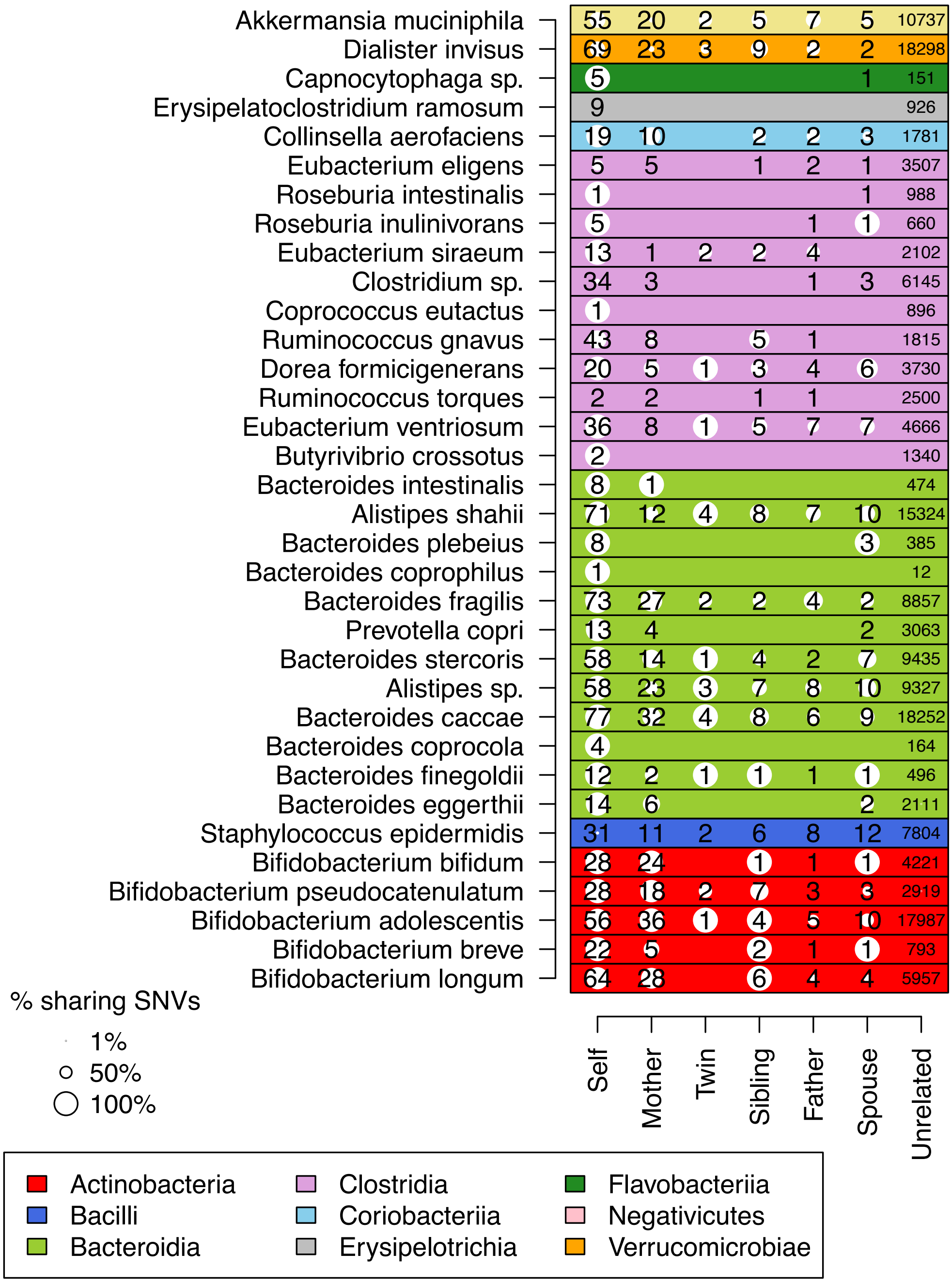

Supplemental Figure S2.
rmSNV similarity between different time points of the same individual, between family members, and between unrelated individuals, by species. The numbers indicate the number of compared sample pairs and the size of the bubble shows the proportion on comparisons sharing >20% of the rmSNVs.
