# Supplementary material for: Selective maternal seeding and environment shape the human gut microbiome
Source: Genome Res. 2018 Apr;28(4):561–8. doi: 10.1101/gr.233940.117 (PMC5880245; doi:10.1101/gr.233940.117)
Supplement: Supplemental Material [file supp_gr.233940.117_Supplemental_Fig_S4.pdf]

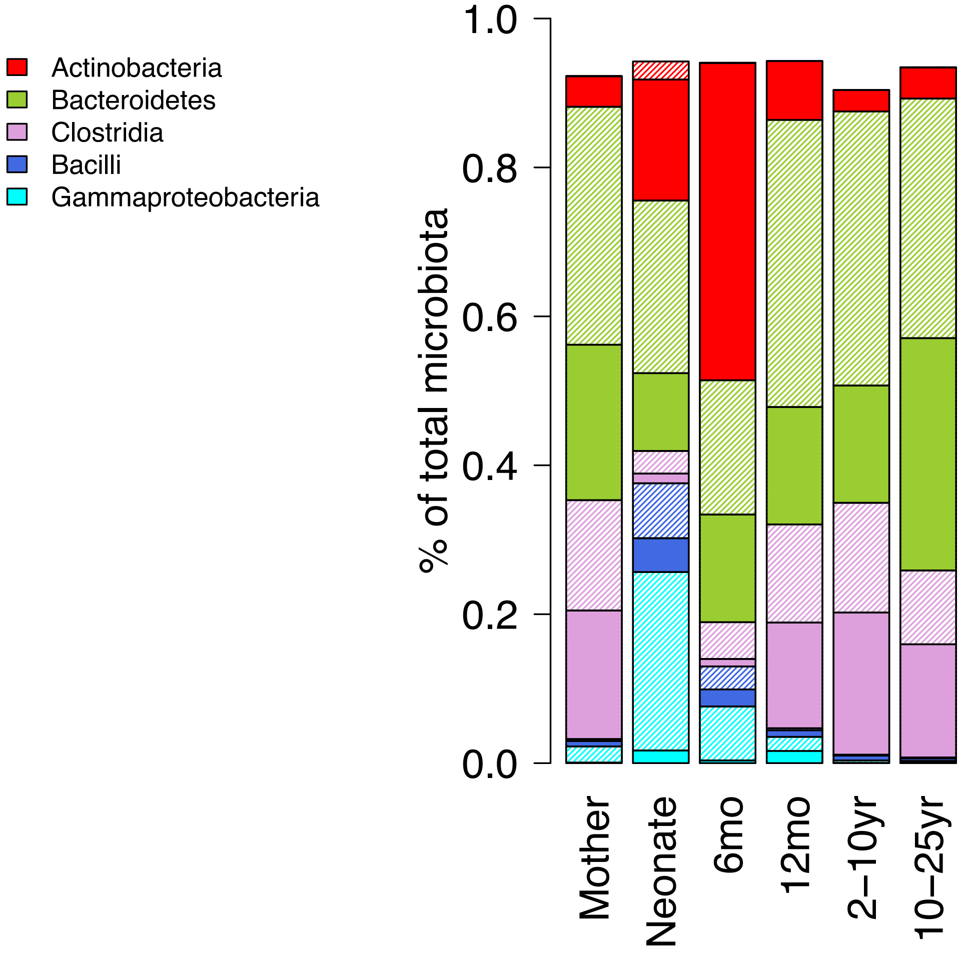

Supplemental Figure S3.
Average relative abundance of the most abundant bacterial taxa at different ages. Solid colours indicate rmSNV-tracked species, and dashed colours species that were not tracked.
