# Supplementary material for: Selective maternal seeding and environment shape the human gut microbiome
Source: Genome Res. 2018 Apr;28(4):561–8. doi: 10.1101/gr.233940.117 (PMC5880245; doi:10.1101/gr.233940.117)
Supplement: Supplemental Material [file supp_gr.233940.117_Supplemental_Fig_S5.pdf]

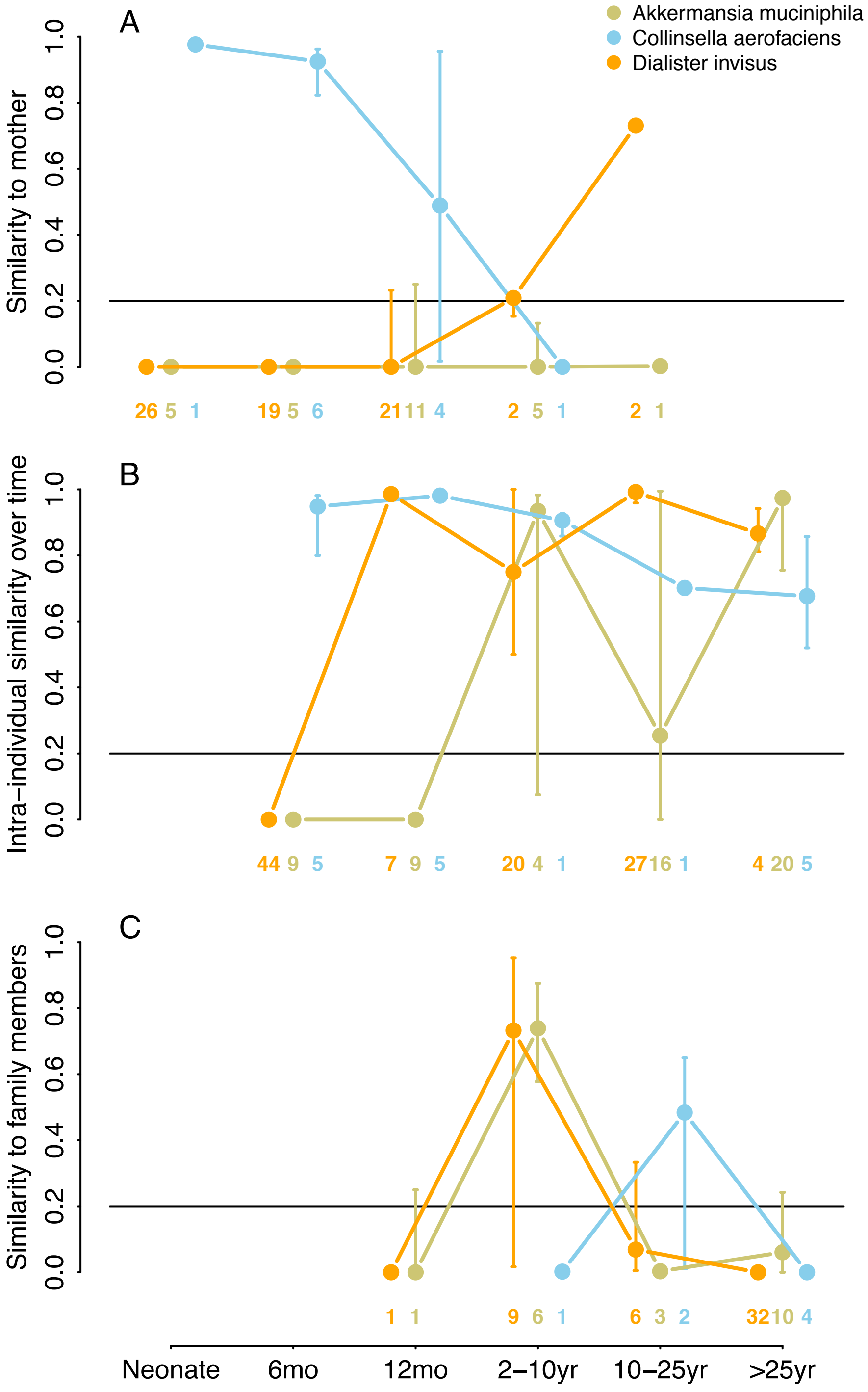

Supplemental Figure S4.
Strain similarity to mother (A), intra-individual similarity over time (B) and similarity to other family members (C) in three bacterial species not belonging to the abundant classes. The symbols represent group medians ± inter-quartile range.
