# Supplementary material for: Selective maternal seeding and environment shape the human gut microbiome
Source: Genome Res. 2018 Apr;28(4):561–8. doi: 10.1101/gr.233940.117 (PMC5880245; doi:10.1101/gr.233940.117)
Supplement: Supplemental Material [file supp_gr.233940.117_Supplemental_Fig_S6.pdf]

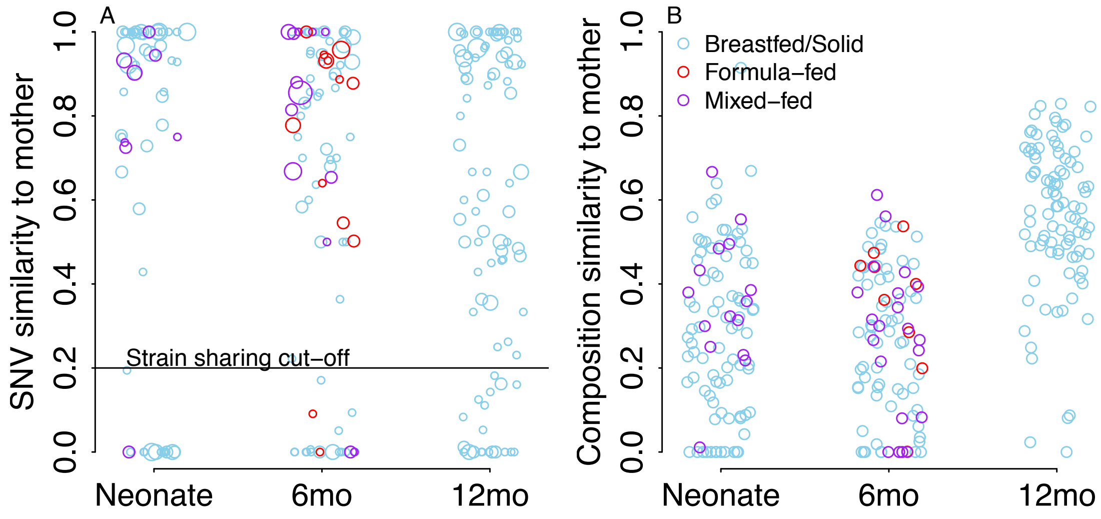

Supplemental Figure S5.
Similarity of SNV populations (A) and species composition (B) to mother by feeding mode. All infants initially received breast milk (exclusively or supplemented with formula), but 14 infants later transitioned to formula feeding before solid foods by age 12 months. None of the diet transitions reduced the stability of maternal SNVs (p=0.45).
