# Supplementary material for: Selective maternal seeding and environment shape the human gut microbiome
Source: Genome Res. 2018 Apr;28(4):561–8. doi: 10.1101/gr.233940.117 (PMC5880245; doi:10.1101/gr.233940.117)
Supplement: Supplemental Material [file supp_gr.233940.117_Supplemental_Fig_S7.pdf]

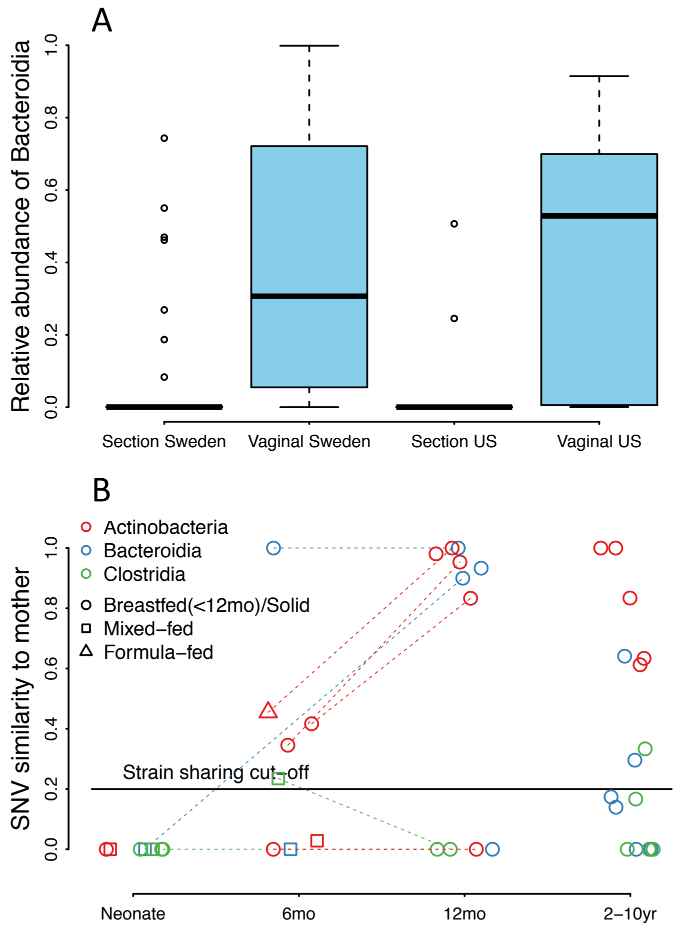

Supplemental Figure S6.
Maternal transmission in Caesarean-born infants. (A) Relative abundance of Bacteroidia in Caesarean-born and vaginally born US and Swedish infants during the first 3 months. (B) Similarity of the rmSNV profiles to mother in Caesarean-born infants at different ages. Each dot represents the median over the tracked species of a class in an individual. Dashed lines connect samples of the same child. At the age of 4 months, 4 out of 6 infants had acquired some maternal strains. However, these appeared to coexist with non-maternal ones, as the median rmSNV similarity to the mother in the species with shared strains was 0.46 (compared to 0.92 in the vaginally born infants, $p<0.001$). By 12 months, 6/9 infants had maternal strains, which had mostly replaced the non-maternal ones, as the median similarity of the species with shared strains had increased to 0.93. Although an obvious source of maternal strains might have been breast milk, in particular as the maternal strains were mainly bifidobacteria, most strains appeared in the faecal samples only after the cessation of breastfeeding, implying limited relevance of this potential transmission route. However, as we did not analyse milk samples, we cannot refute transfer of bifidobacteria from breast milk. Of the 2-10 year old Caesarean-born children, 5 of 6 had maternal strains, and all had paternal and fraternal strains. The maternal strains were nearly always also shared with other family members, which suggests they were obtained from the environment, rather than directly from the mother at birth.
