# Supplementary material for: Selective maternal seeding and environment shape the human gut microbiome
Source: Genome Res. 2018 Apr;28(4):561–8. doi: 10.1101/gr.233940.117 (PMC5880245; doi:10.1101/gr.233940.117)
Supplement: Supplemental Material [file supp_gr.233940.117_Supplemental_Fig_S8.pdf]

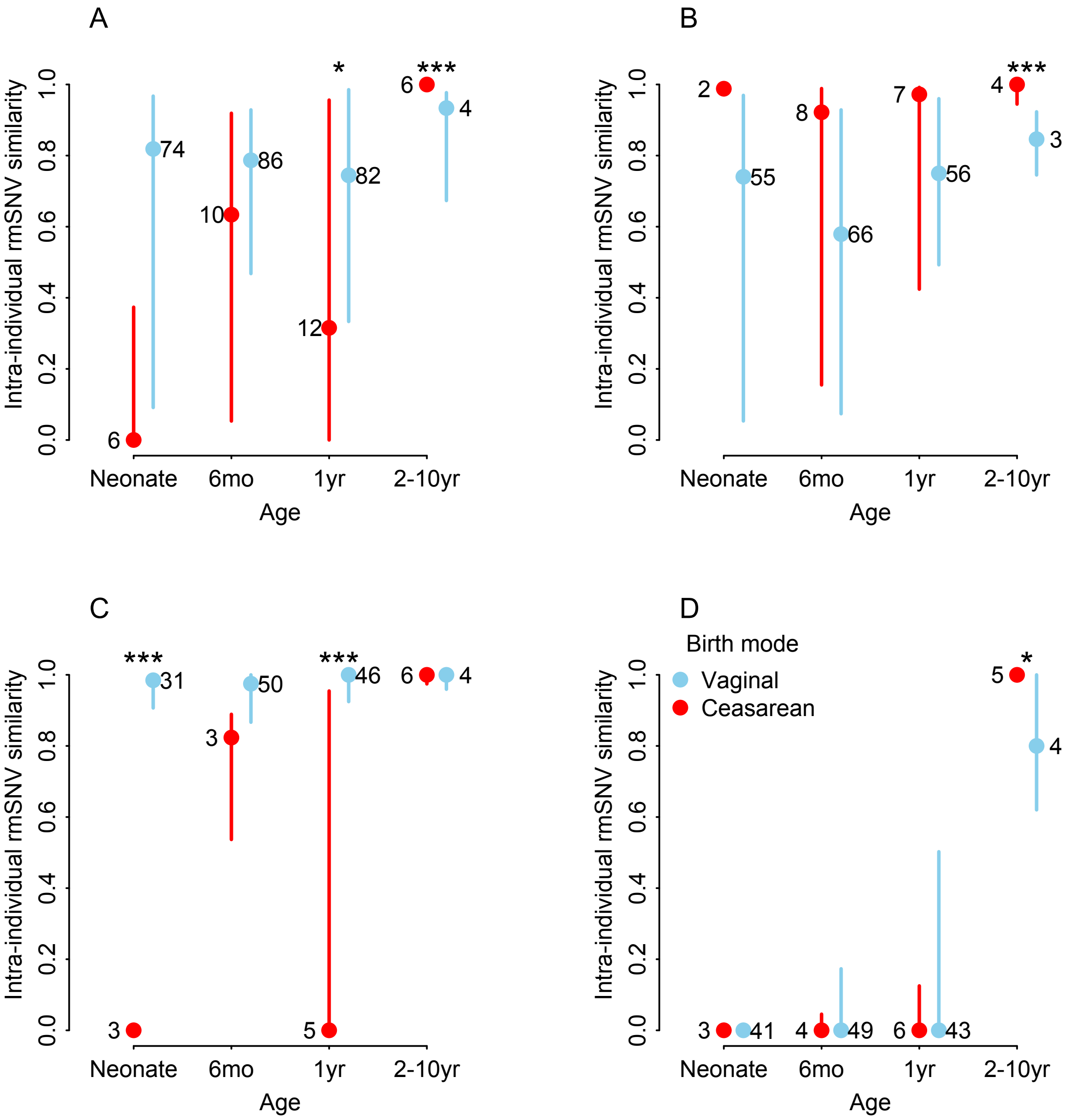

Supplemental Figure S7.
Intra-individual strain population stability (rmSNV profile similarity) at different ages by birth mode. The circles represent group medians, and the lines span the inter-quartile range. Significant differences between the birth modes are indicate with asterisks (beta regression; * $p<0.05$; *** $p < 0.001$). (A) All monitored species. (B) Actinobacteria. (C) Bacteroidia. (D) Clostridia.
