# Supplementary material for: Selective maternal seeding and environment shape the human gut microbiome
Source: Genome Res. 2018 Apr;28(4):561–8. doi: 10.1101/gr.233940.117 (PMC5880245; doi:10.1101/gr.233940.117)
Supplement: Supplemental Material [file supp_gr.233940.117_Supplemental_Fig_S9.pdf]

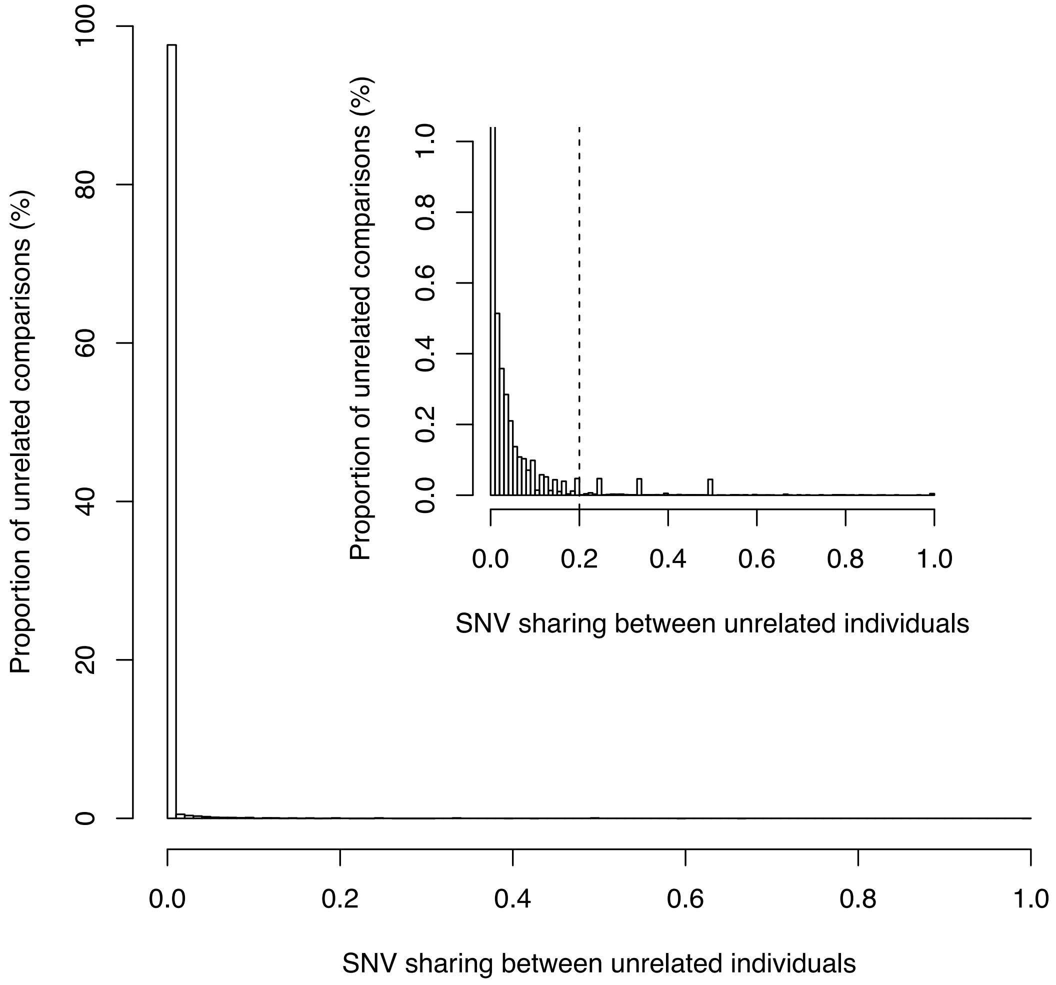

Supplemental Figure S8.
Histogram of rmSNV sharing between unrelated individuals. The small panel is a zoomed-in version of the larger panel. The dashed line denotes the cut-off used as evidence of strain sharing.
