# Supplementary material for: Selective maternal seeding and environment shape the human gut microbiome
Source: Genome Res. 2018 Apr;28(4):561–8. doi: 10.1101/gr.233940.117 (PMC5880245; doi:10.1101/gr.233940.117)
Supplement: Supplemental Material [file supp_gr.233940.117_Supplemental_Fig_S10.pdf]

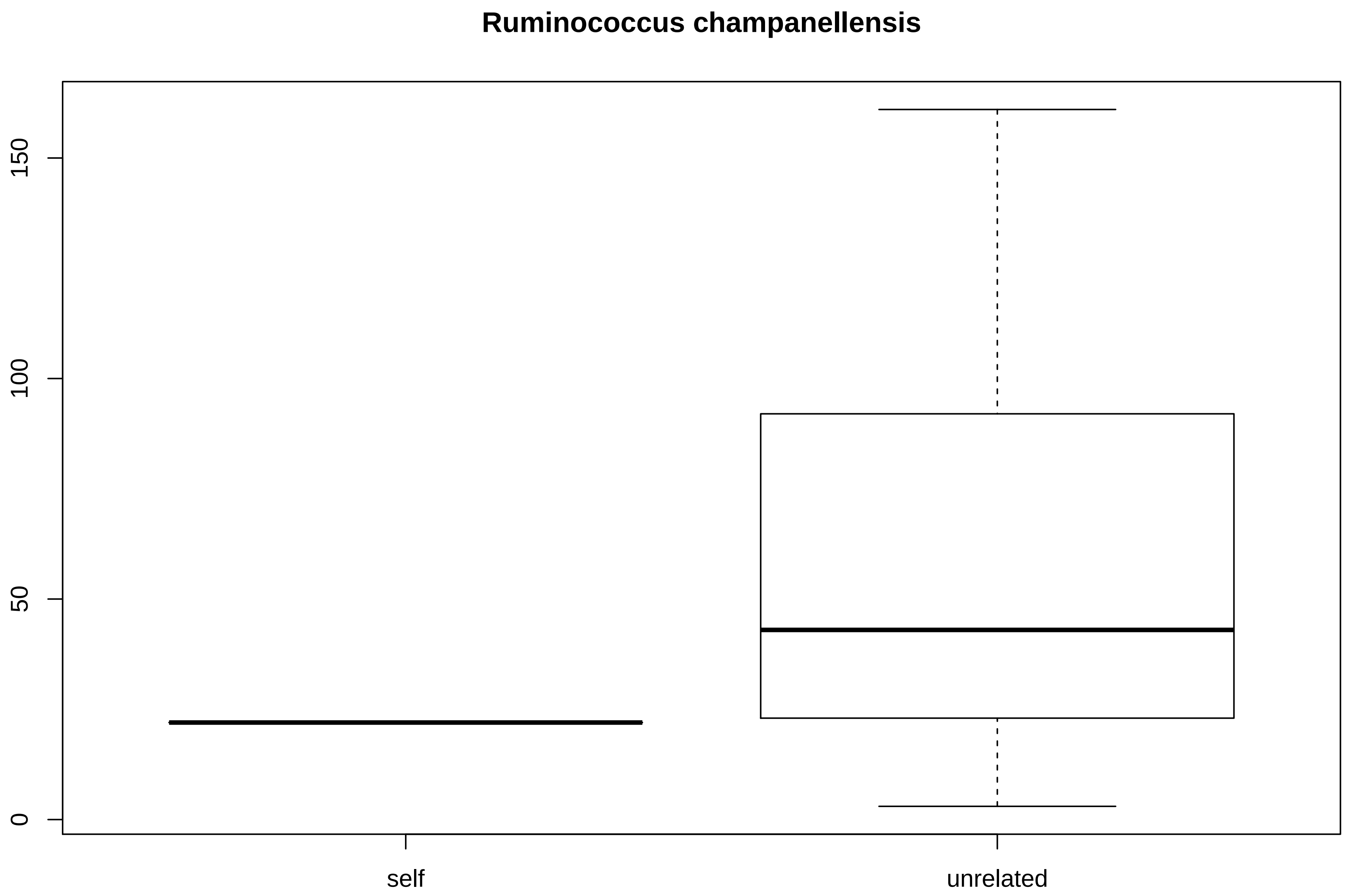

Ruminococcus champanellensis
0
50
100
150
self
unrelated

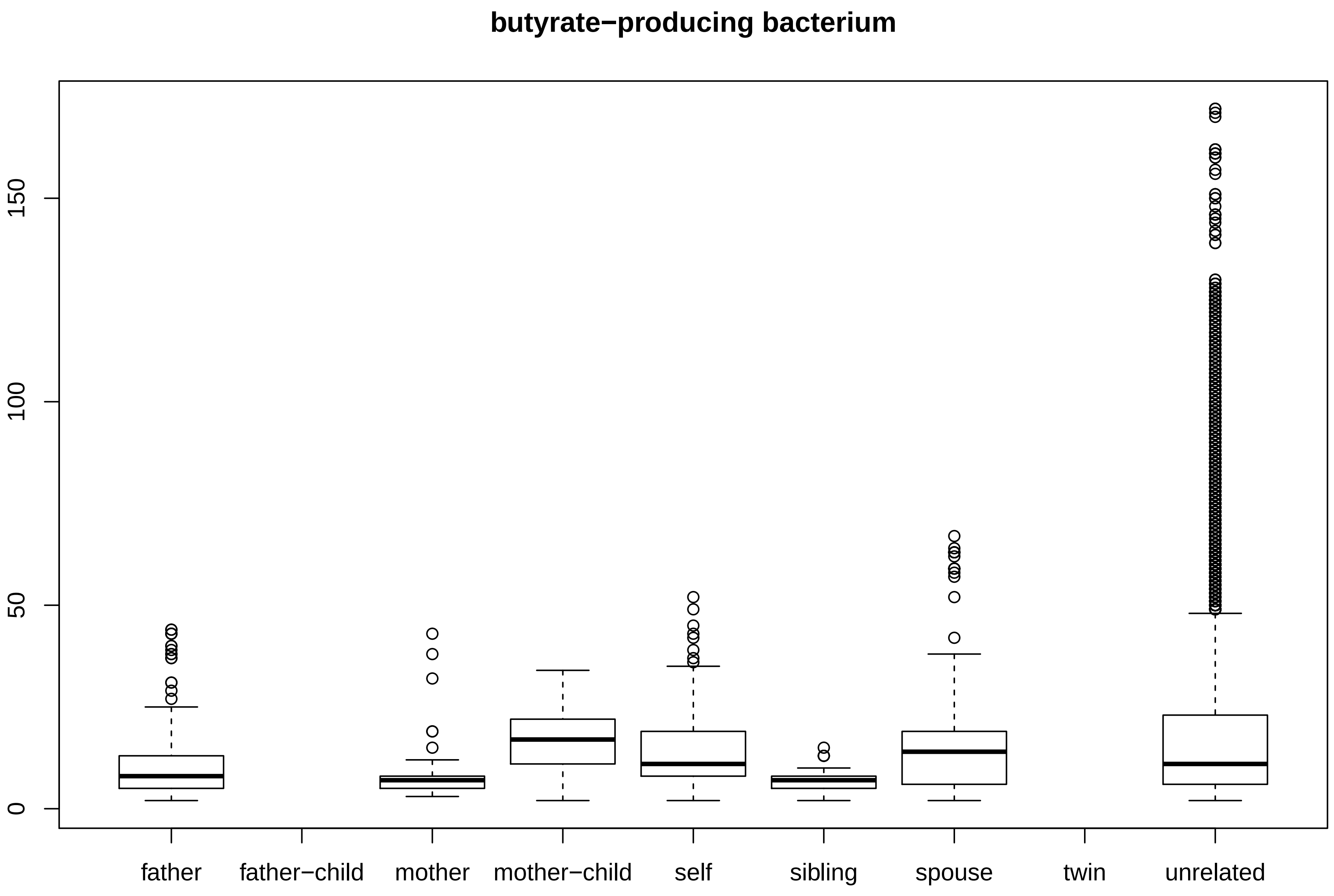
butyrate−producing bacterium
0
50
100
150
father
father−child
mother
mother−child
self
sibling
spouse
twin
unrelated

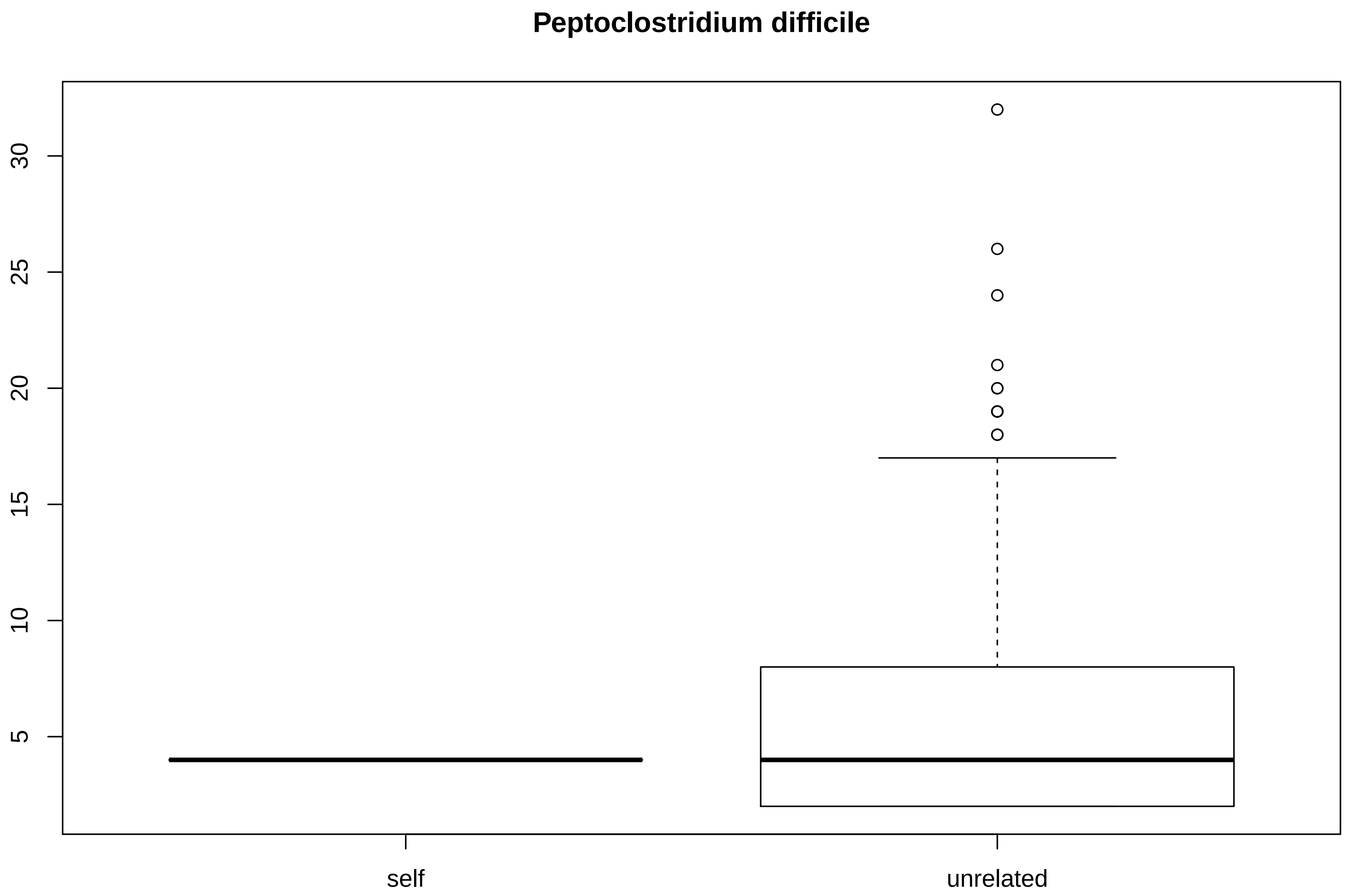
Peptoclostridium difficile
30
25
20
15
10
5
self
unrelated

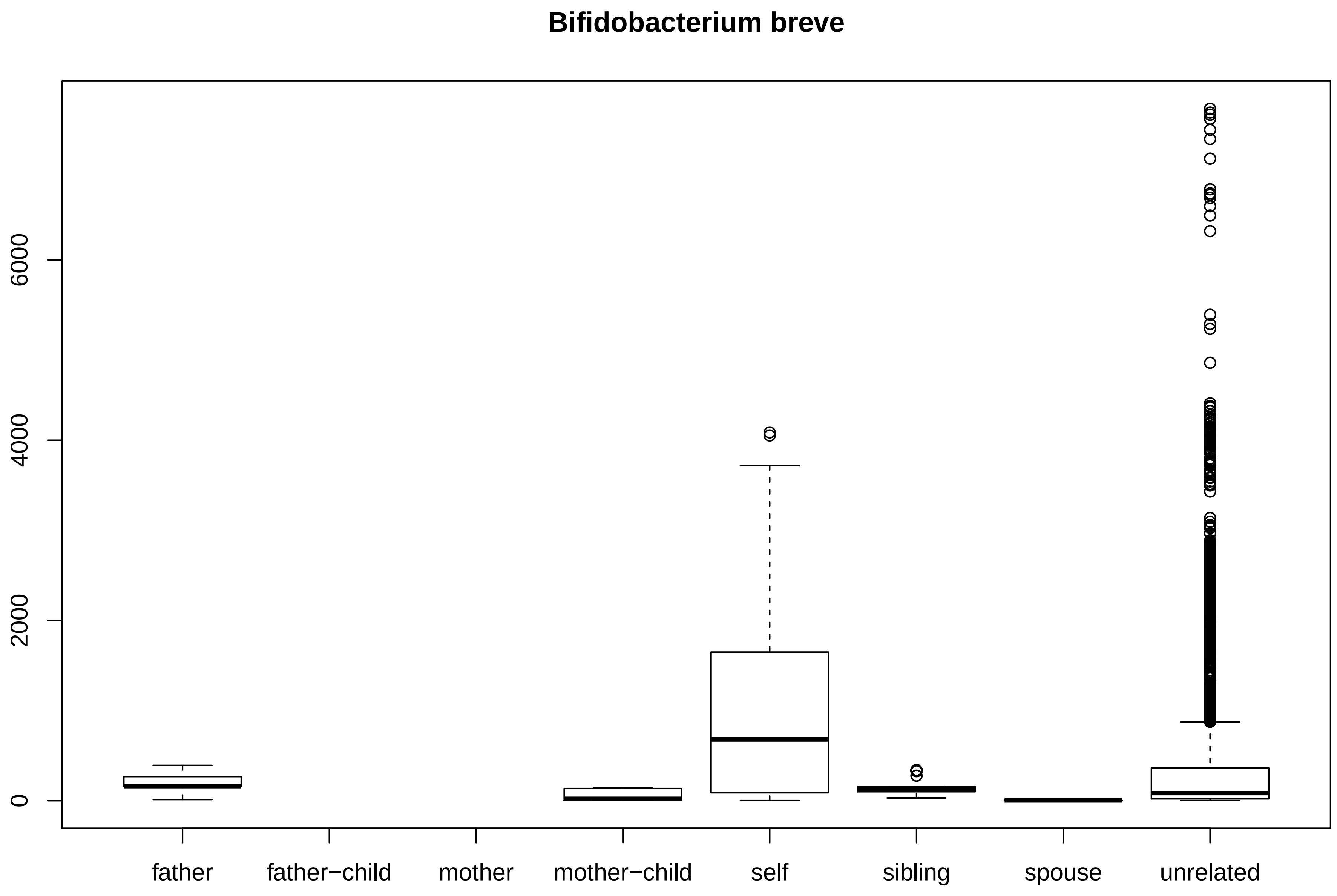

Bifidobacterium breve
0
2000
4000
6000
father
father−child
mother
mother−child
self
sibling
spouse
unrelated

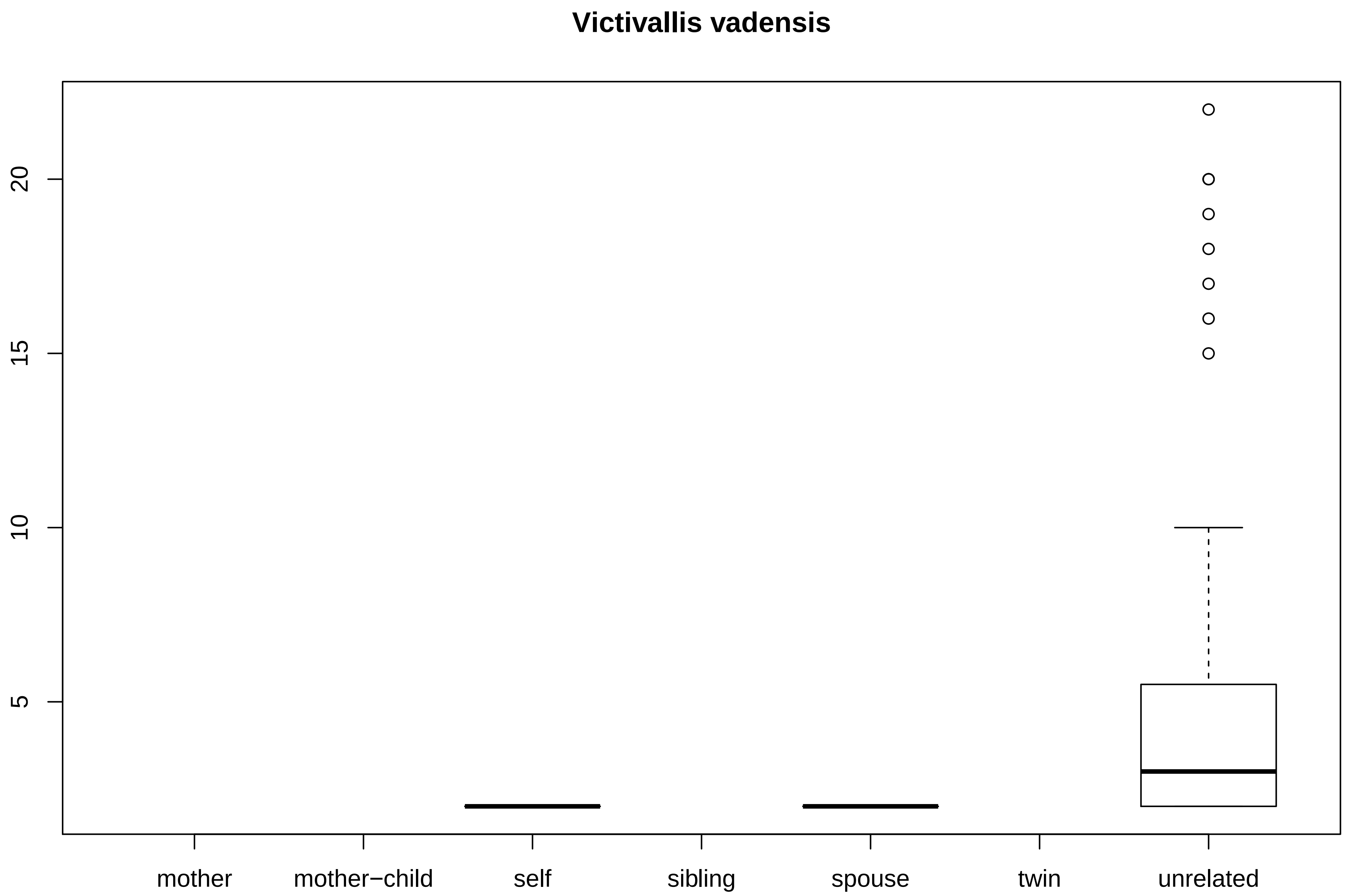
Victivallis vadensis
5
10
15
20
mother
mother−child
self
sibling
spouse
twin
unrelated

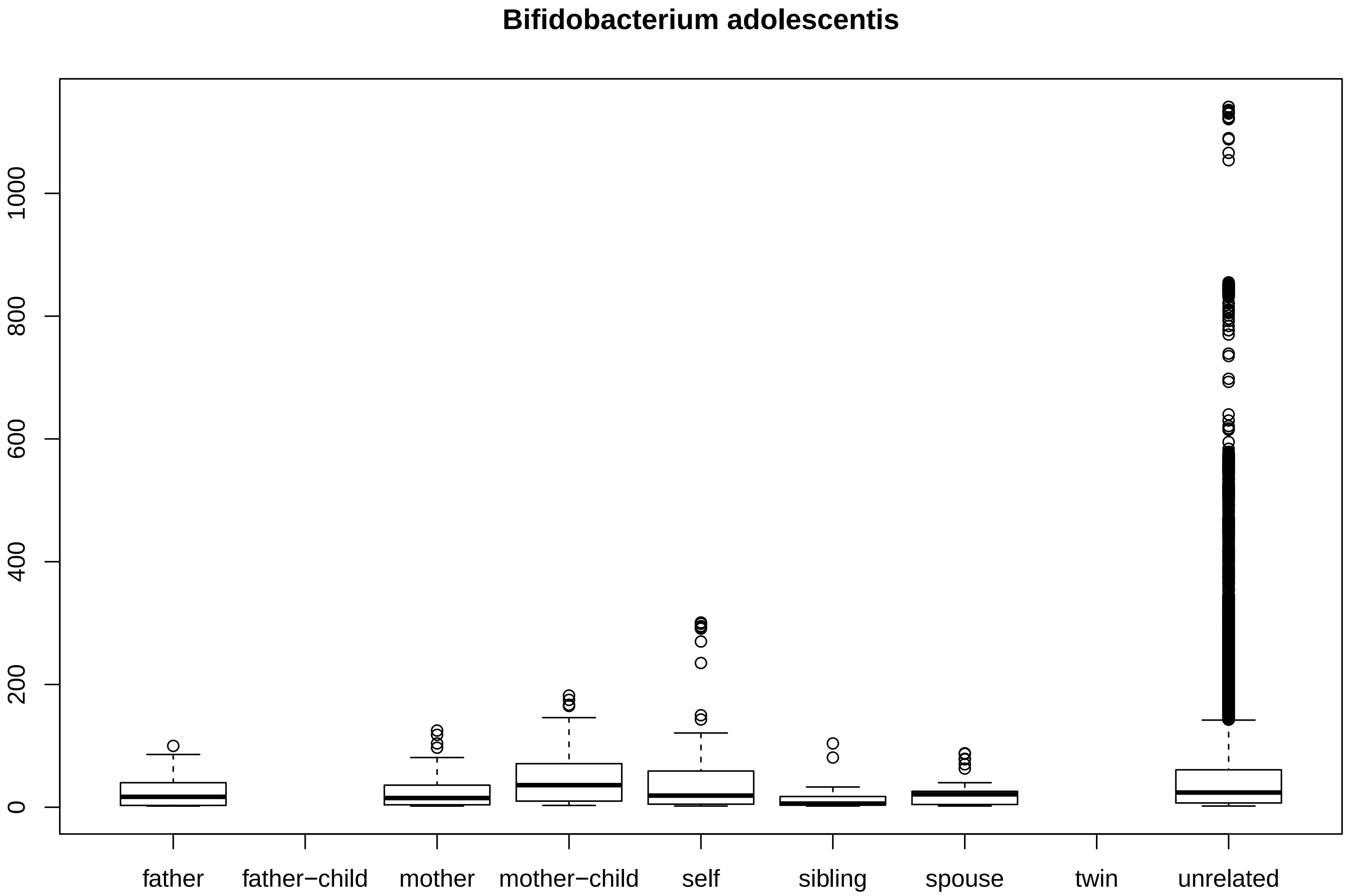
Bifidobacterium adolescentis
0
200
400
600
800
1000
father
father−child
mother
mother−child
self
sibling
spouse
twin
unrelated

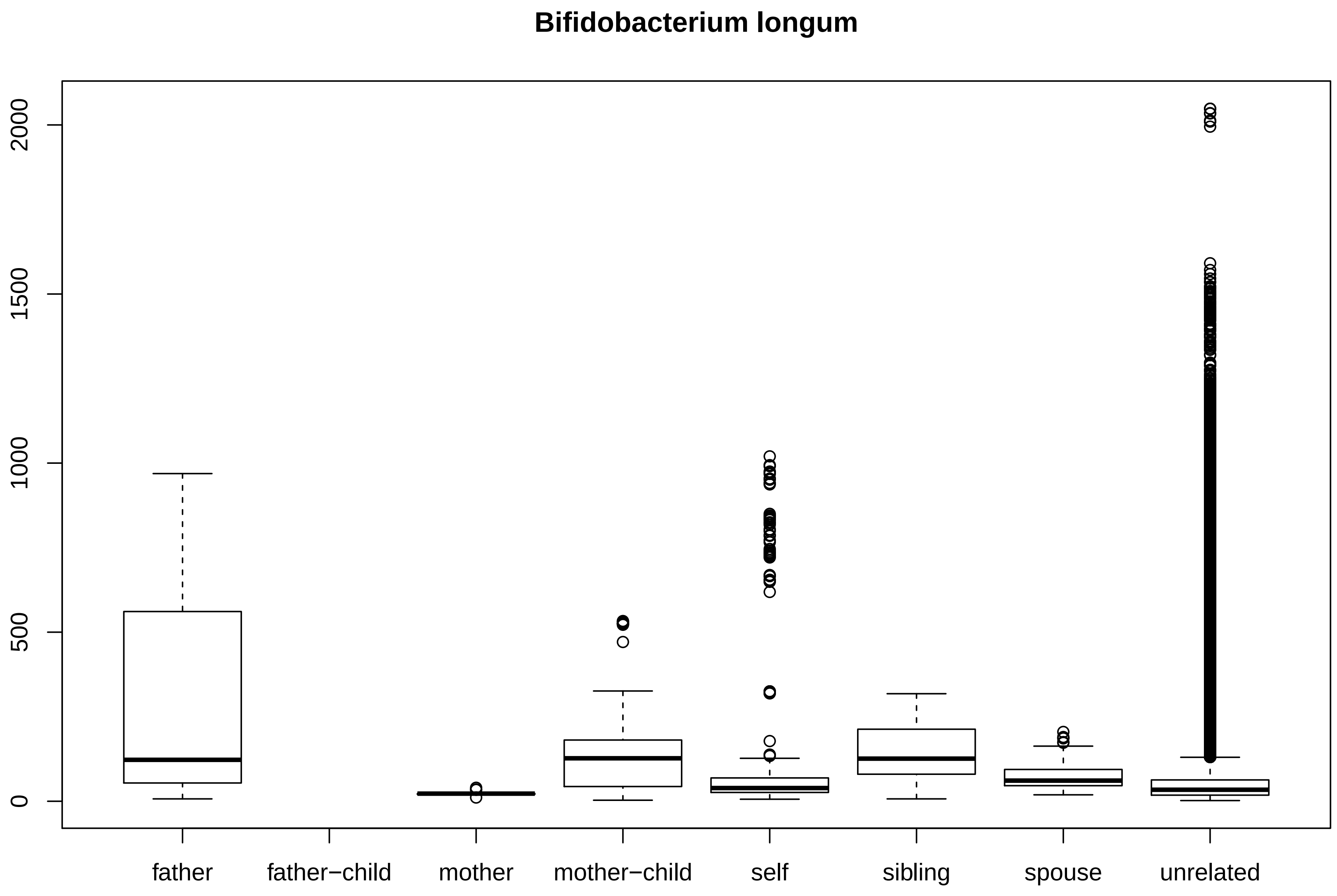
Bifidobacterium longum
0
500
1000
1500
2000
father
father−child
mother
mother−child
self
sibling
spouse
unrelated

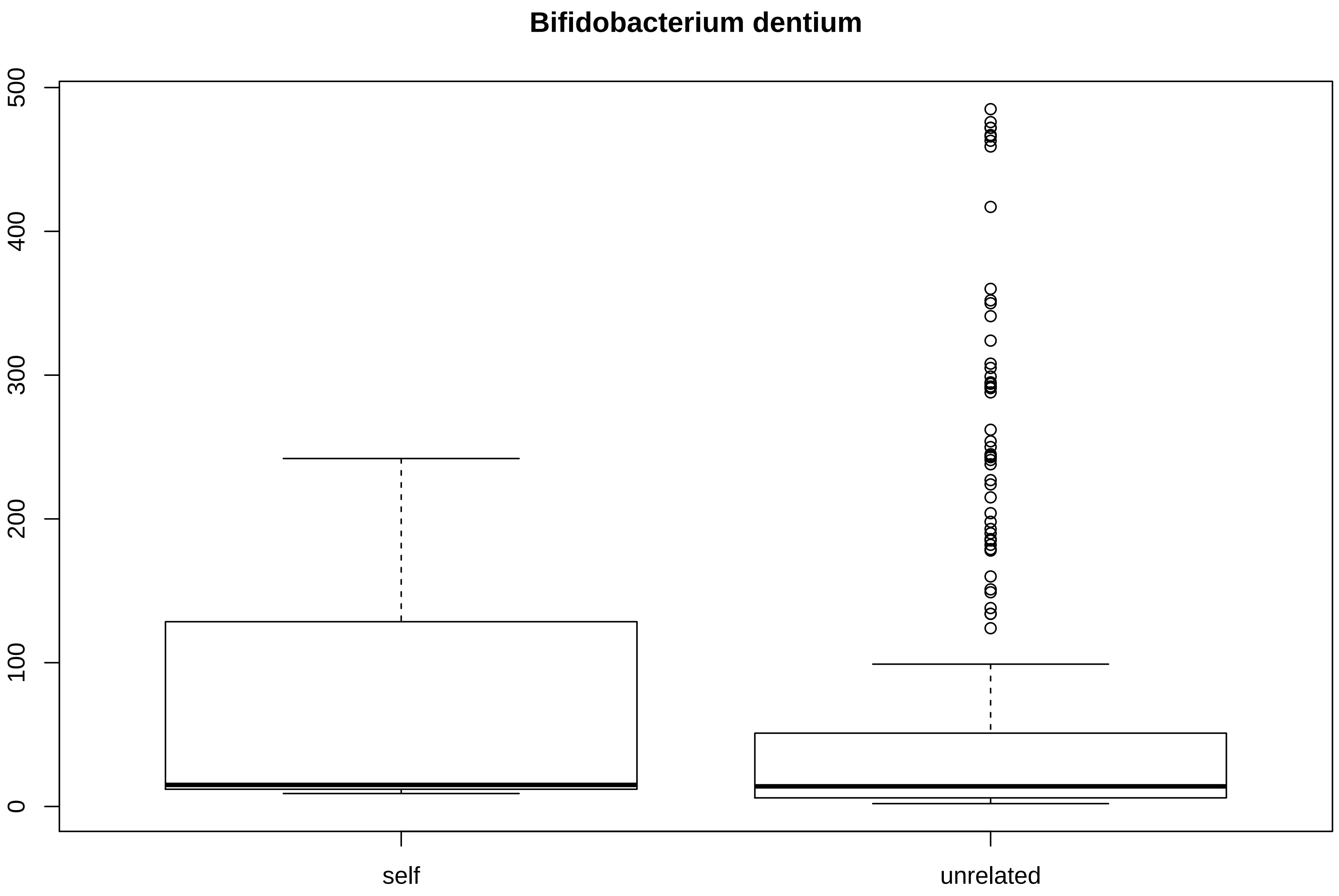
Bifidobacterium dentium
0
100
200
300
400
500
self
unrelated

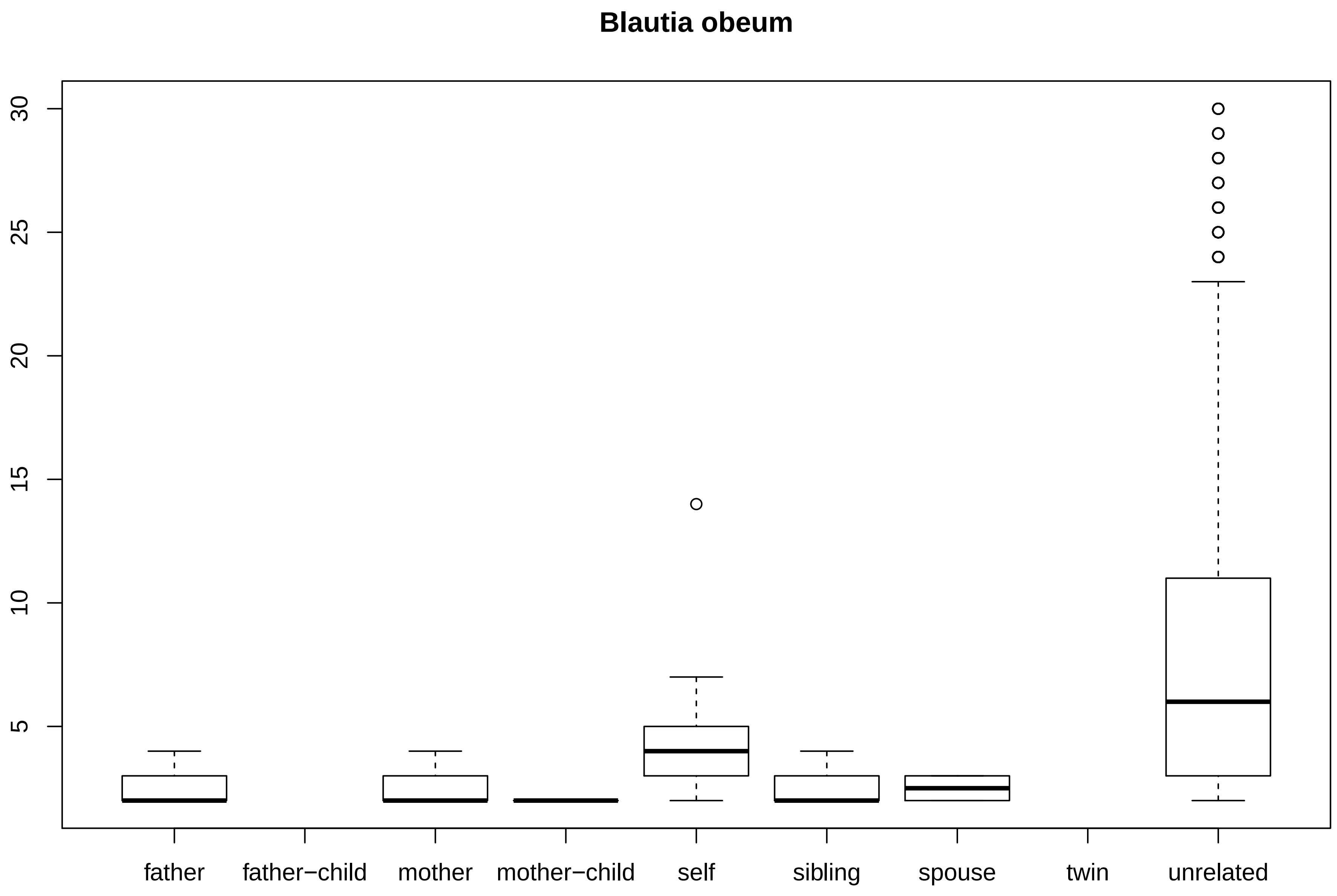
Blautia obeum
30
25
20
15
10
5
father
father−child
mother
mother−child
self
sibling
spouse
twin
unrelated

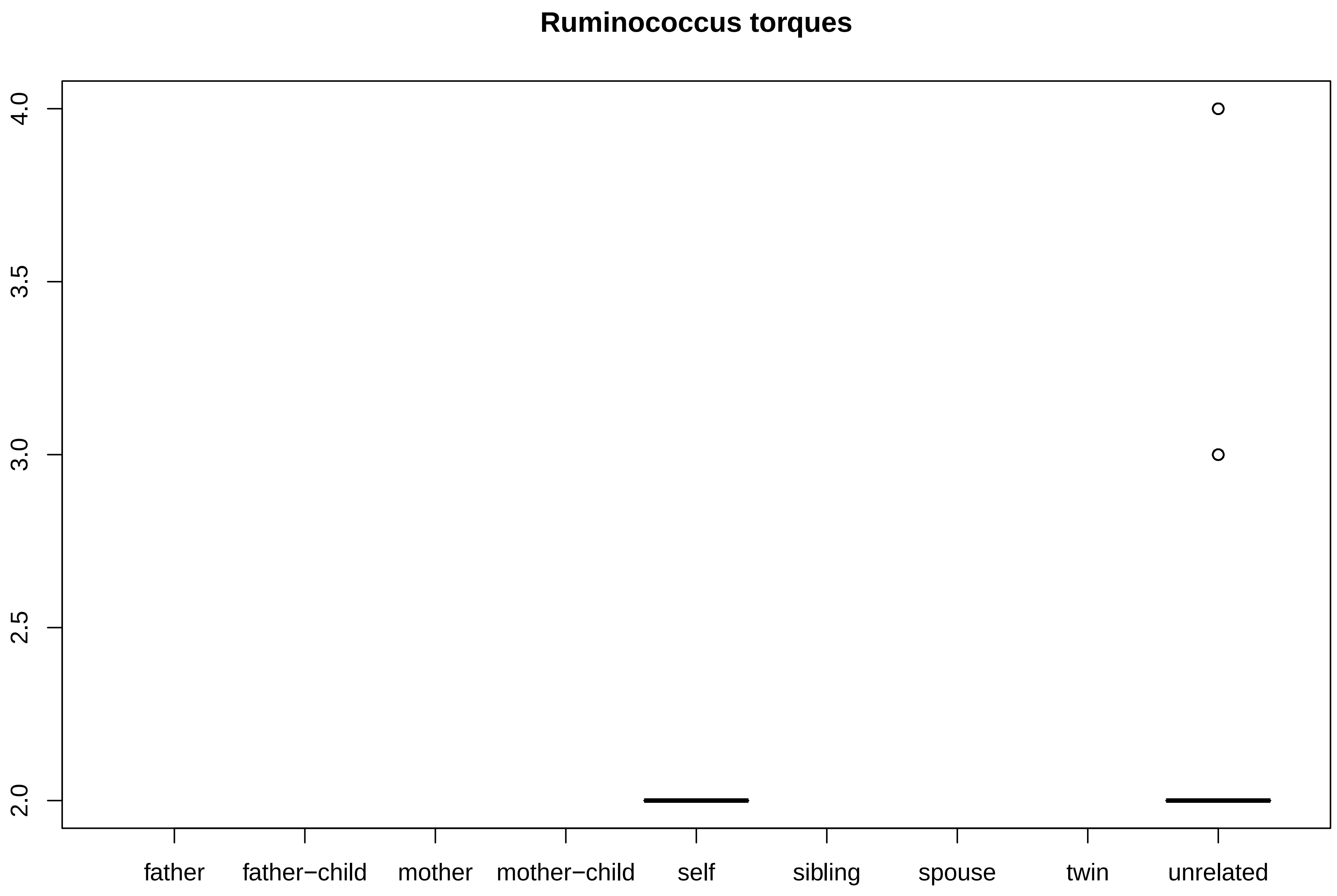
Ruminococcus torques
4.0
3.5
3.0
2.5
2.0
father
father−child
mother
mother−child
self
sibling
spouse
twin
unrelated

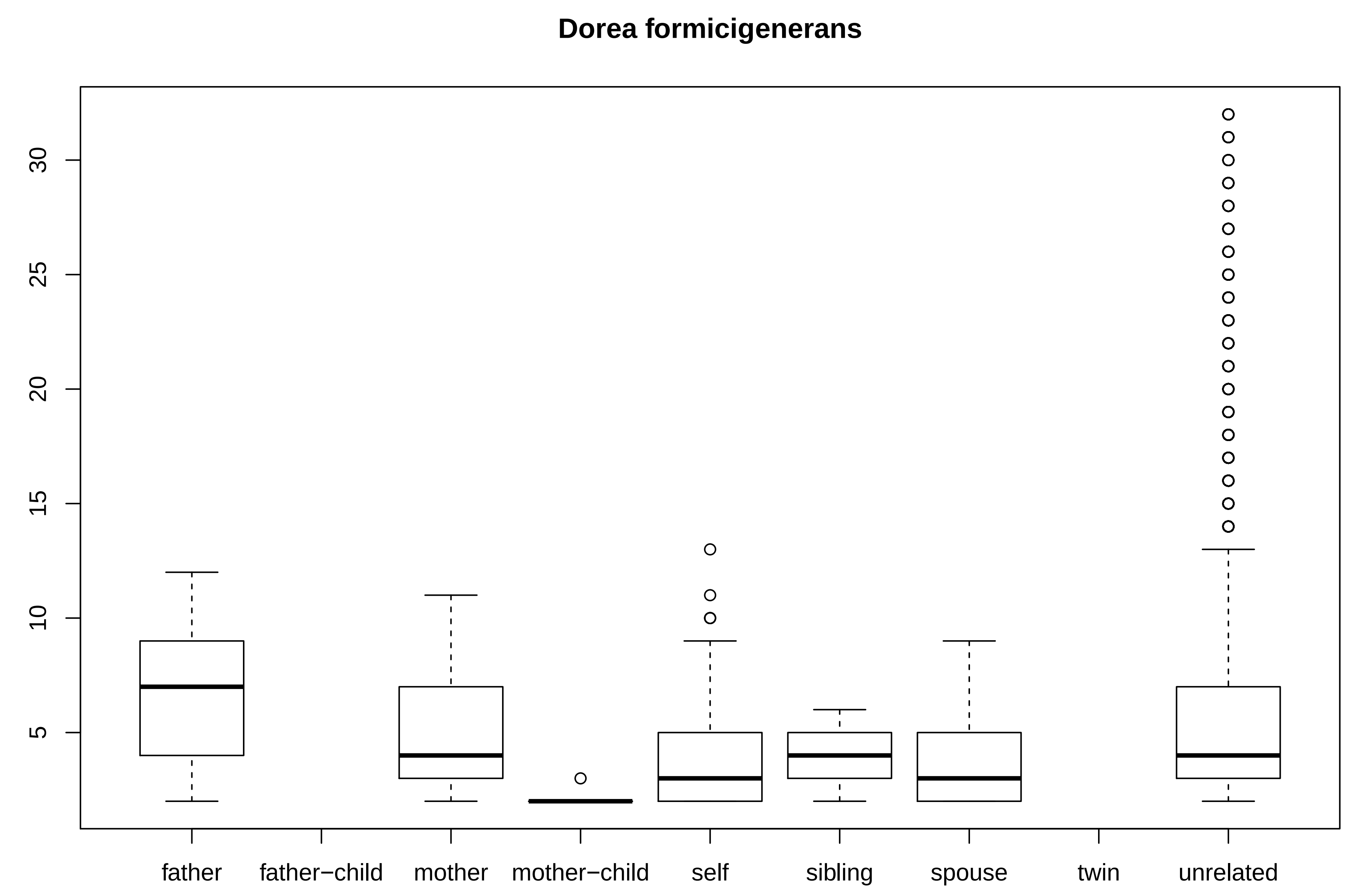

Dorea formicigenerans
5
10
15
20
25
30
father
father−child
mother
mother−child
self
sibling
spouse
twin
unrelated

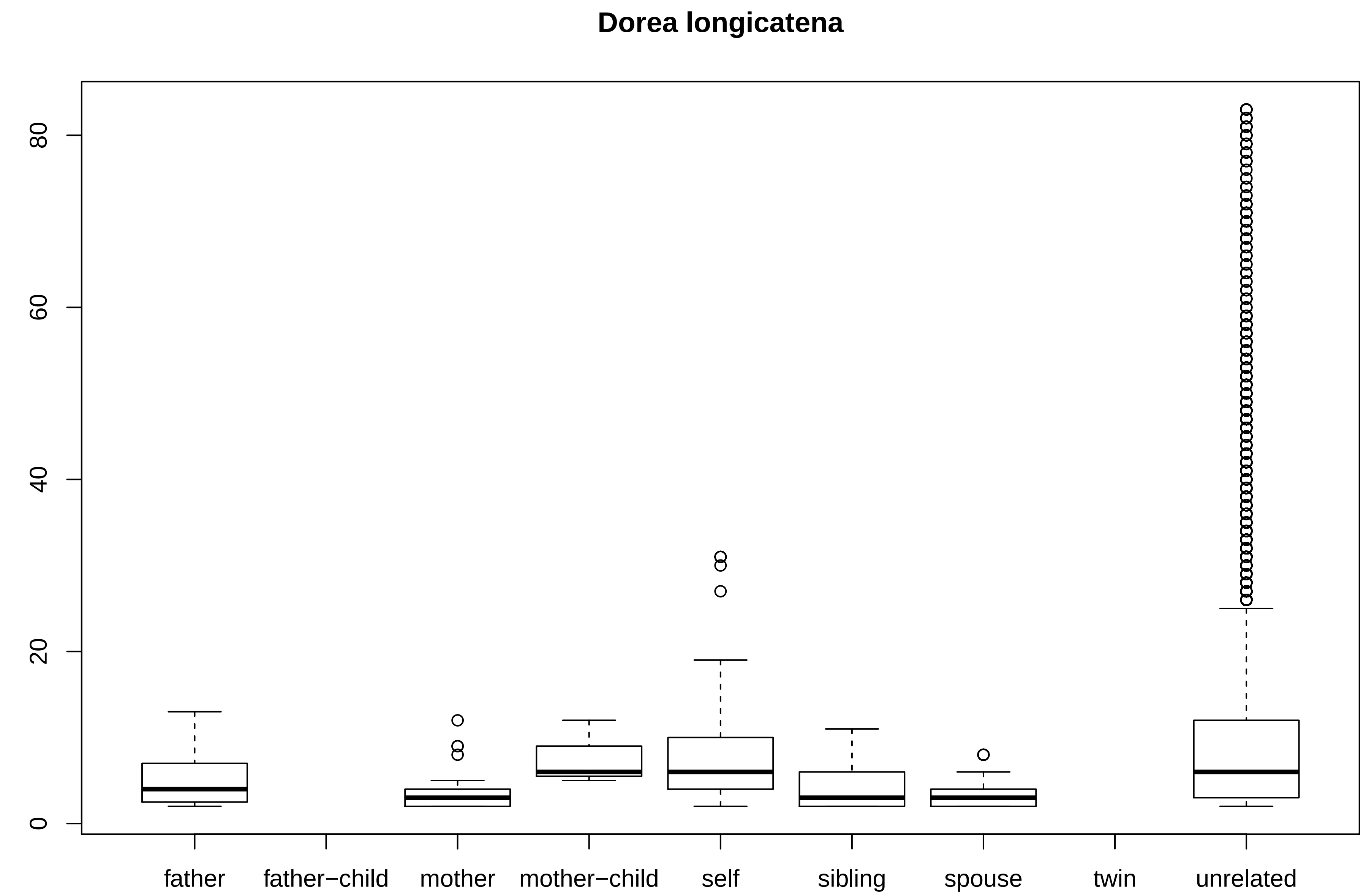
Dorea longicatena
0
20
40
60
80
father
father−child
mother
mother−child
self
sibling
spouse
twin
unrelated

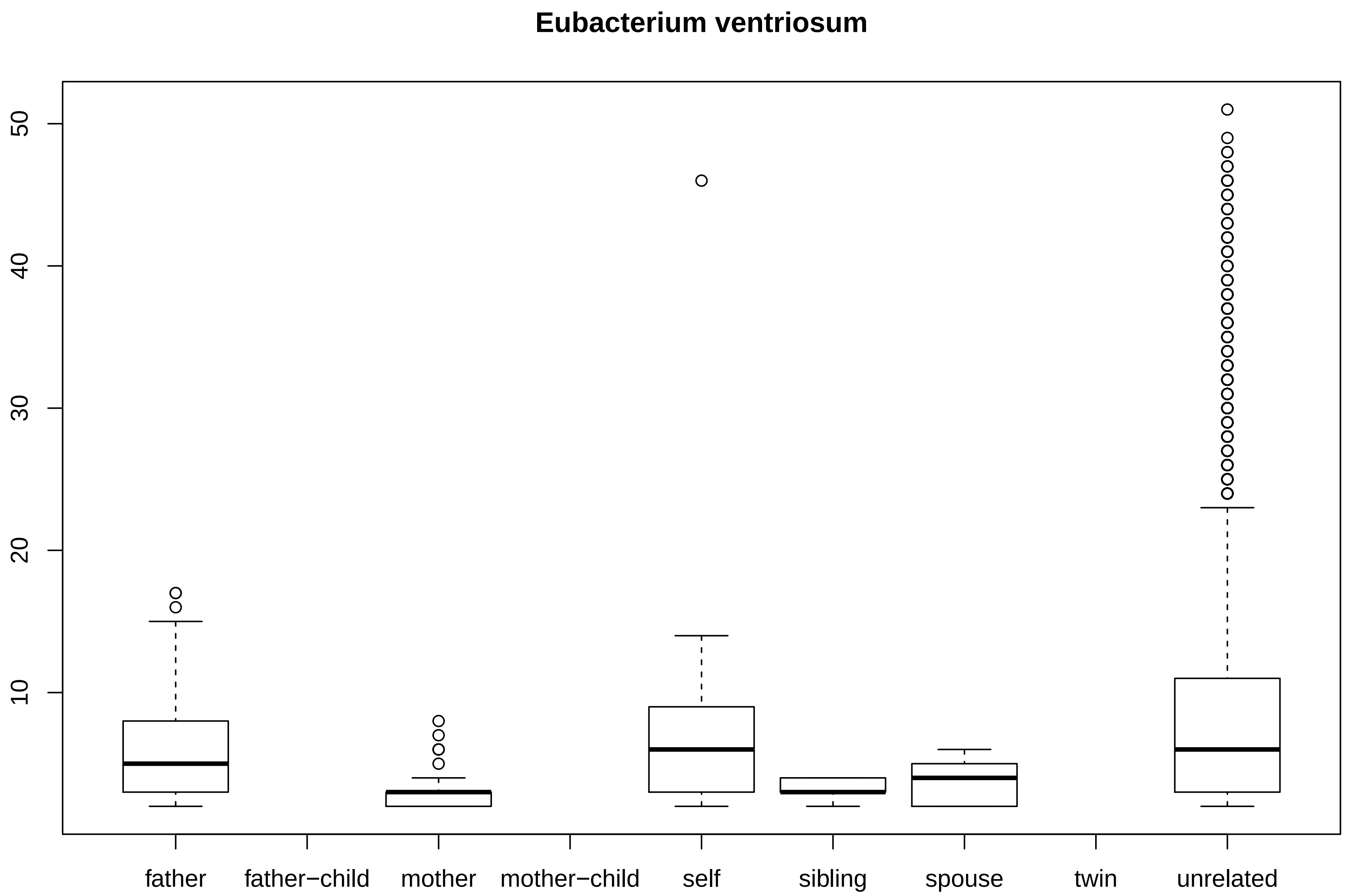
Eubacterium ventriosum
10
20
30
40
50
father
father−child
mother
mother−child
self
sibling
spouse
twin
unrelated

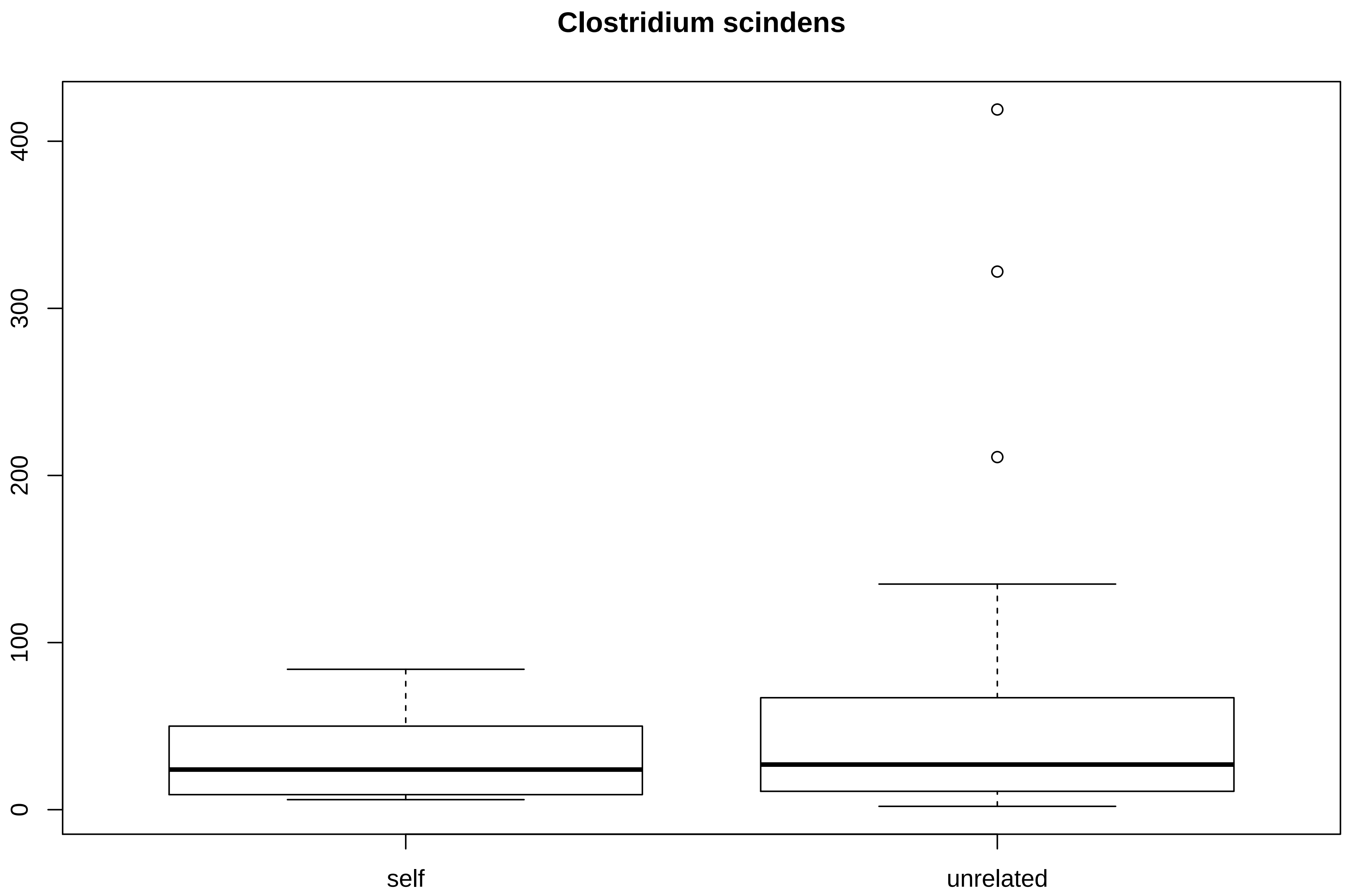

Clostridium scindens
0
100
200
300
400
self
unrelated

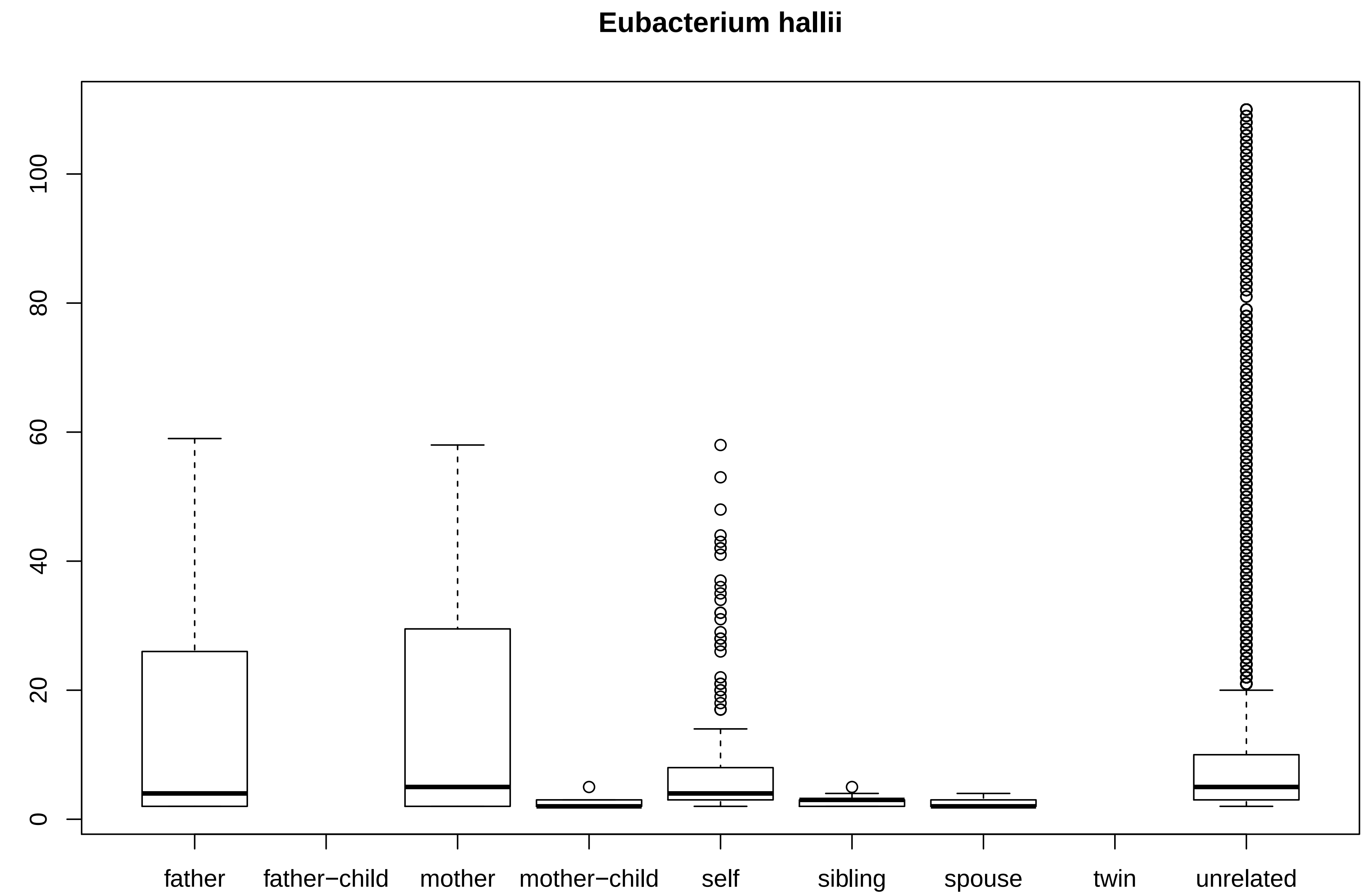
Eubacterium hallii
0
20
40
60
80
100
father
father−child
mother
mother−child
self
sibling
spouse
twin
unrelated

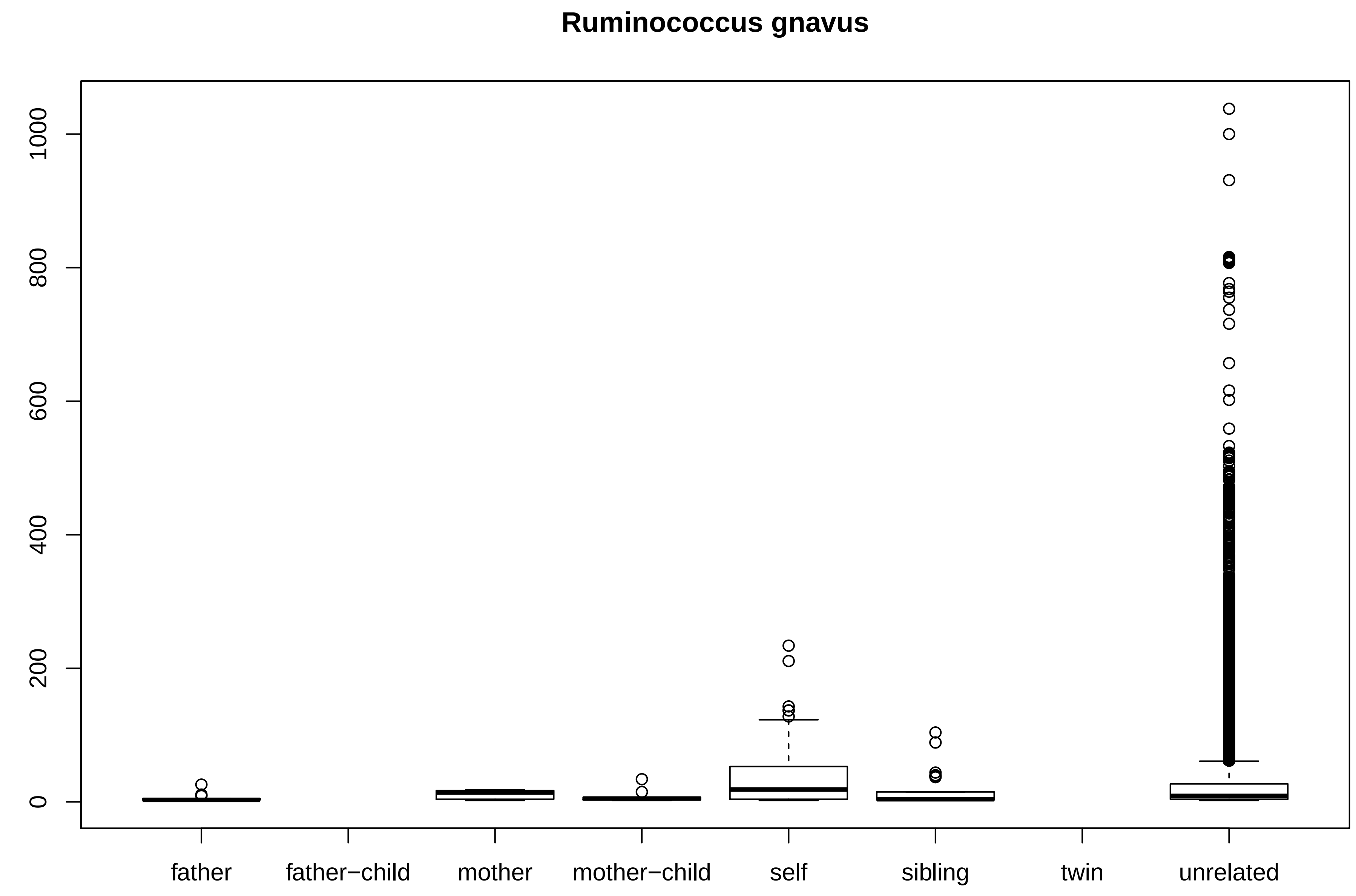
Ruminococcus gnavus
0
200
400
600
800
1000
father
father−child
mother
mother−child
self
sibling
twin
unrelated

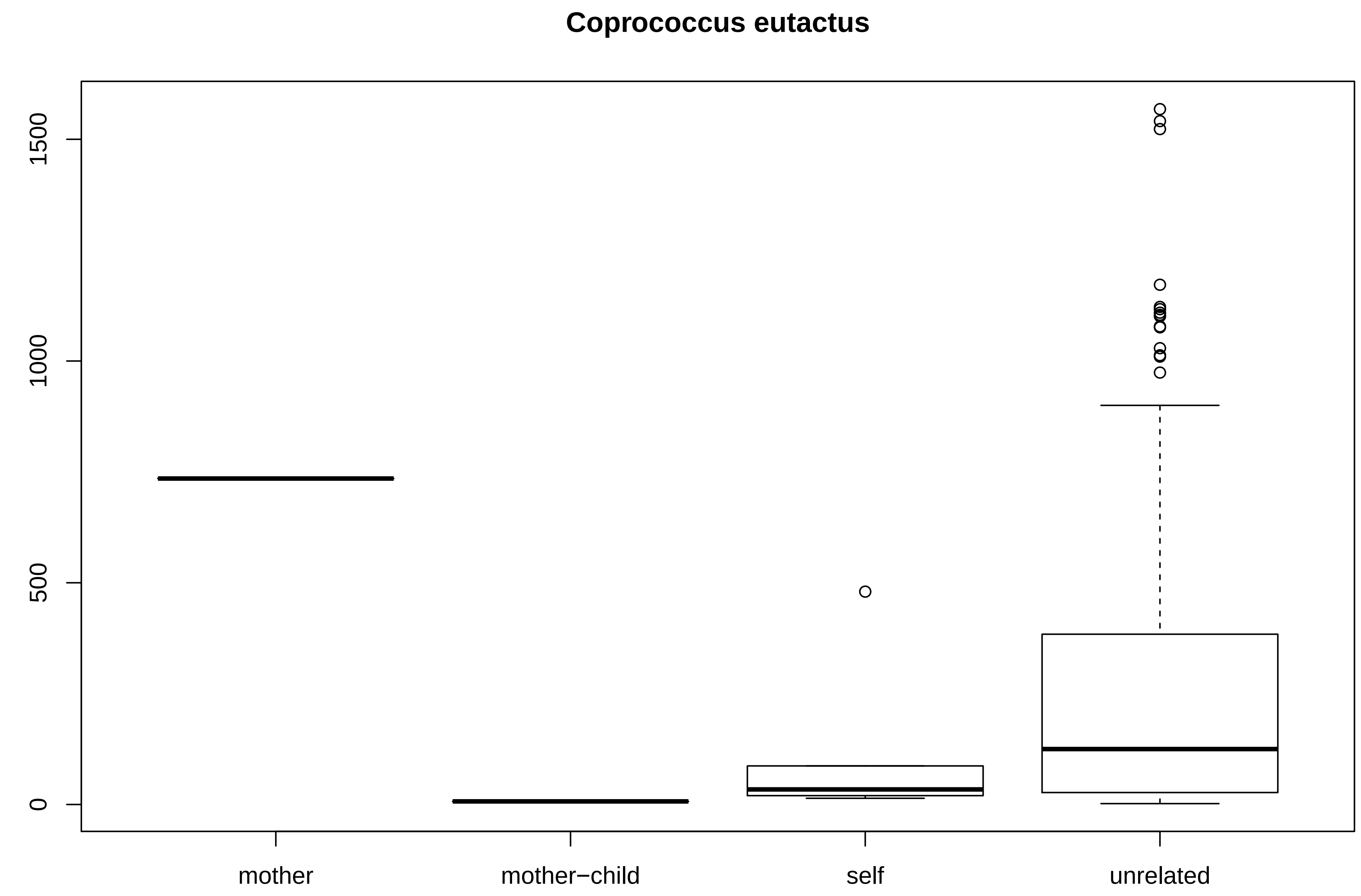

Coprococcus eutactus
0
500
1000
1500
mother
mother−child
self
unrelated

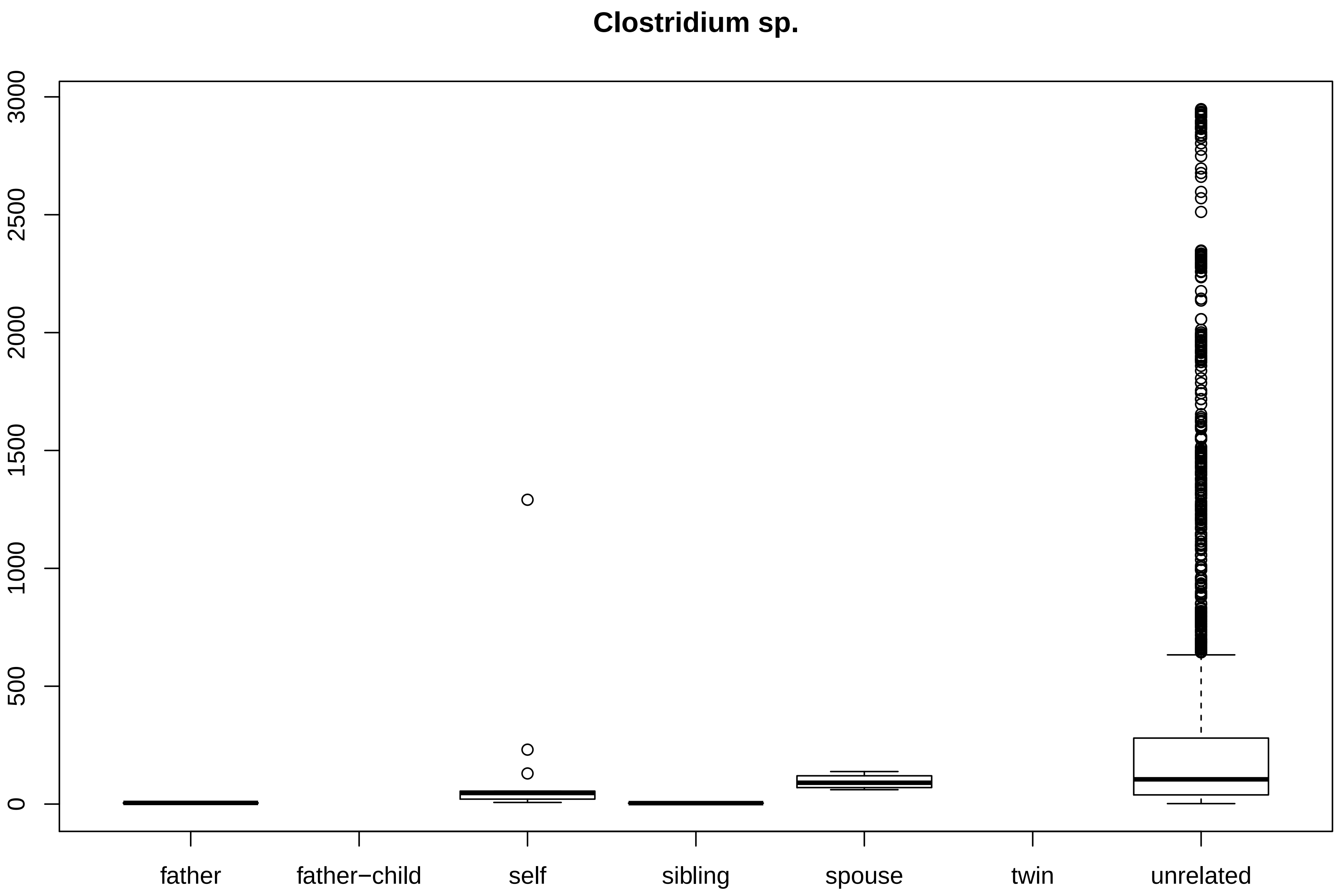
Clostridium sp.
0
500
1000
1500
2000
2500
3000
father
father−child
self
sibling
spouse
twin
unrelated

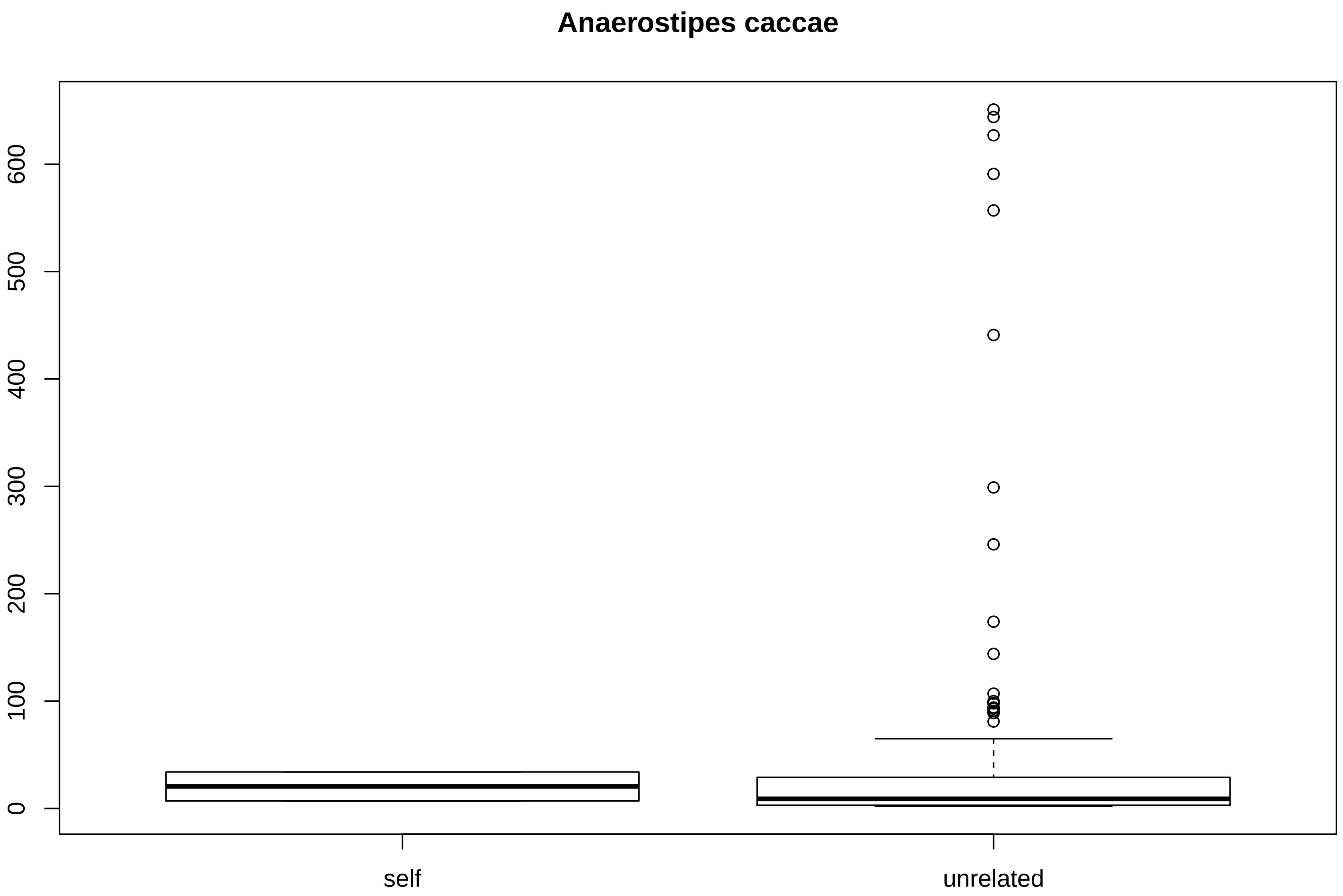
Anaerostipes caccae
0
100
200
300
400
500
600
self
unrelated

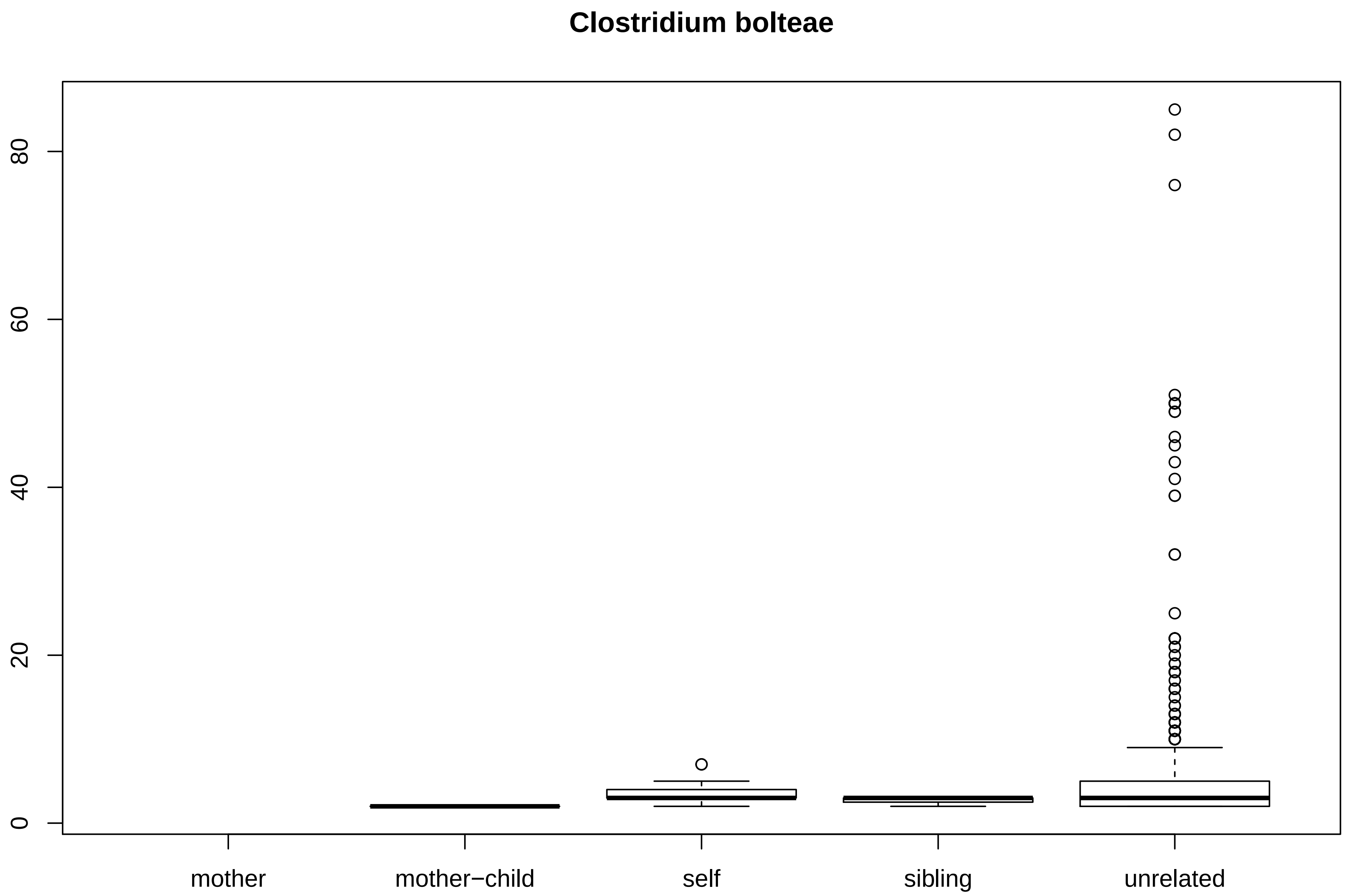
Clostridium bolteae
0
20
40
60
80
mother
mother−child
self
sibling
unrelated

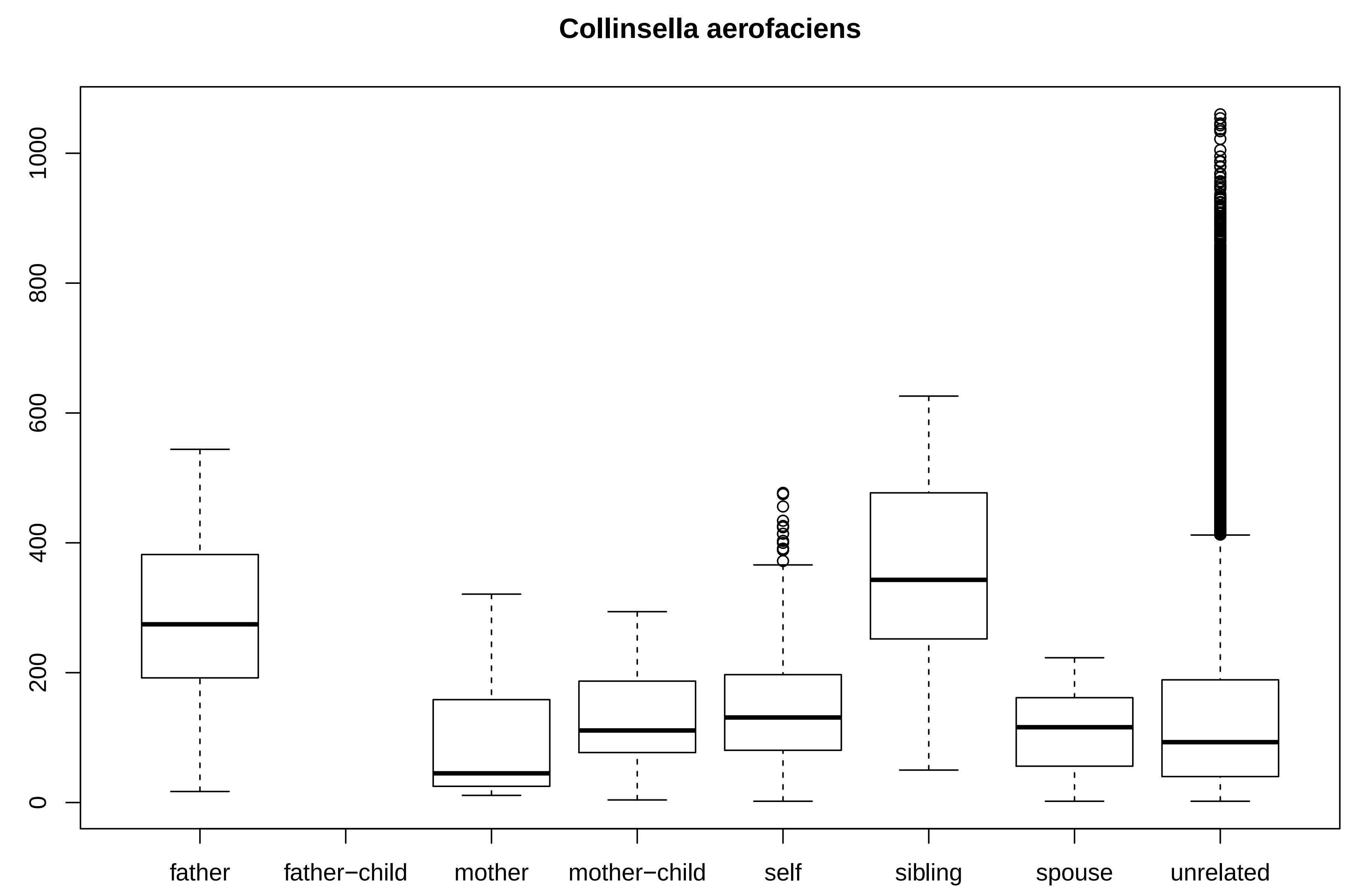
Collinsella aerofaciens
0
200
400
600
800
1000
father
father−child
mother
mother−child
self
sibling
spouse
unrelated

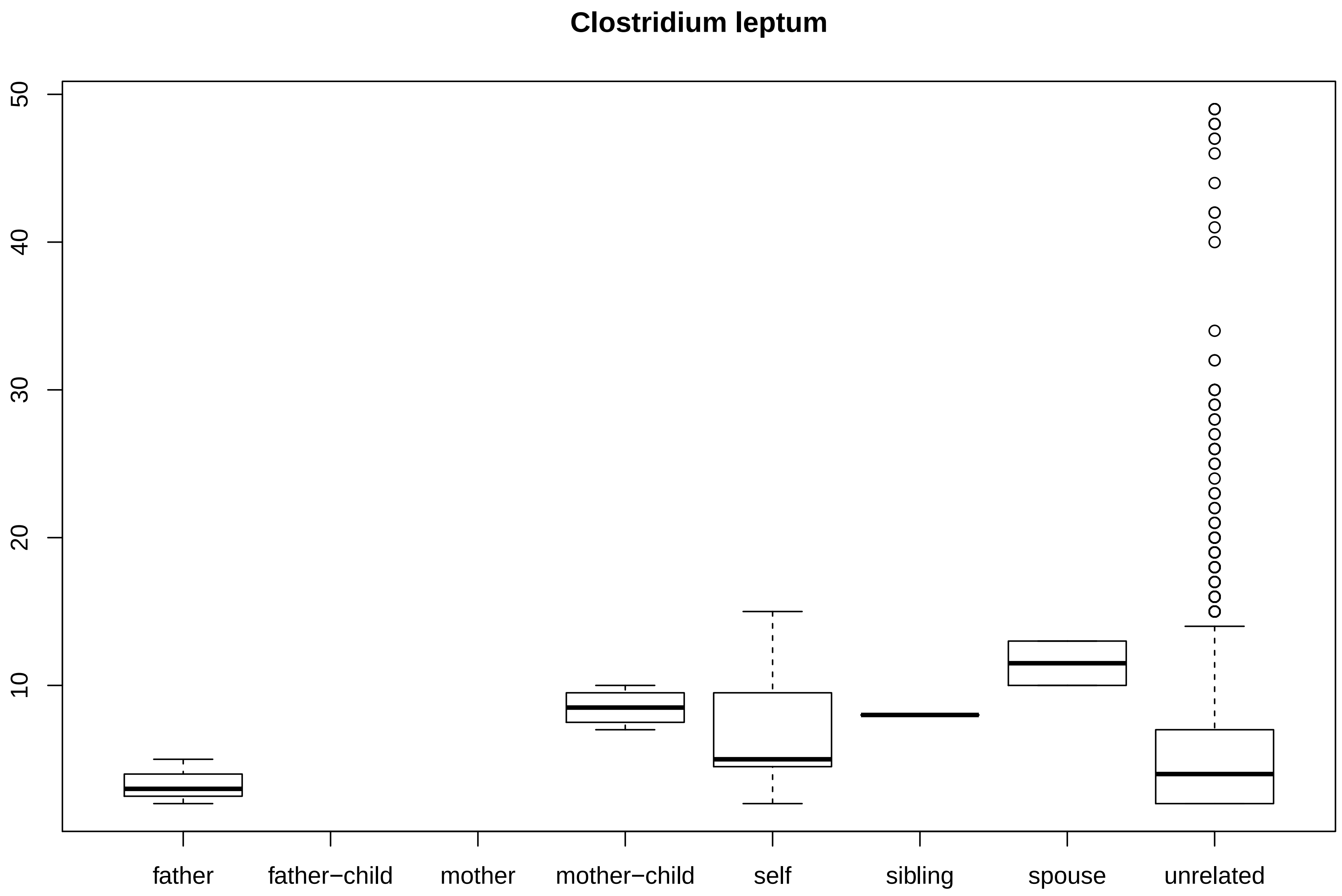
Clostridium leptum
50
40
30
20
10
father
father−child
mother
mother−child
self
sibling
spouse
unrelated

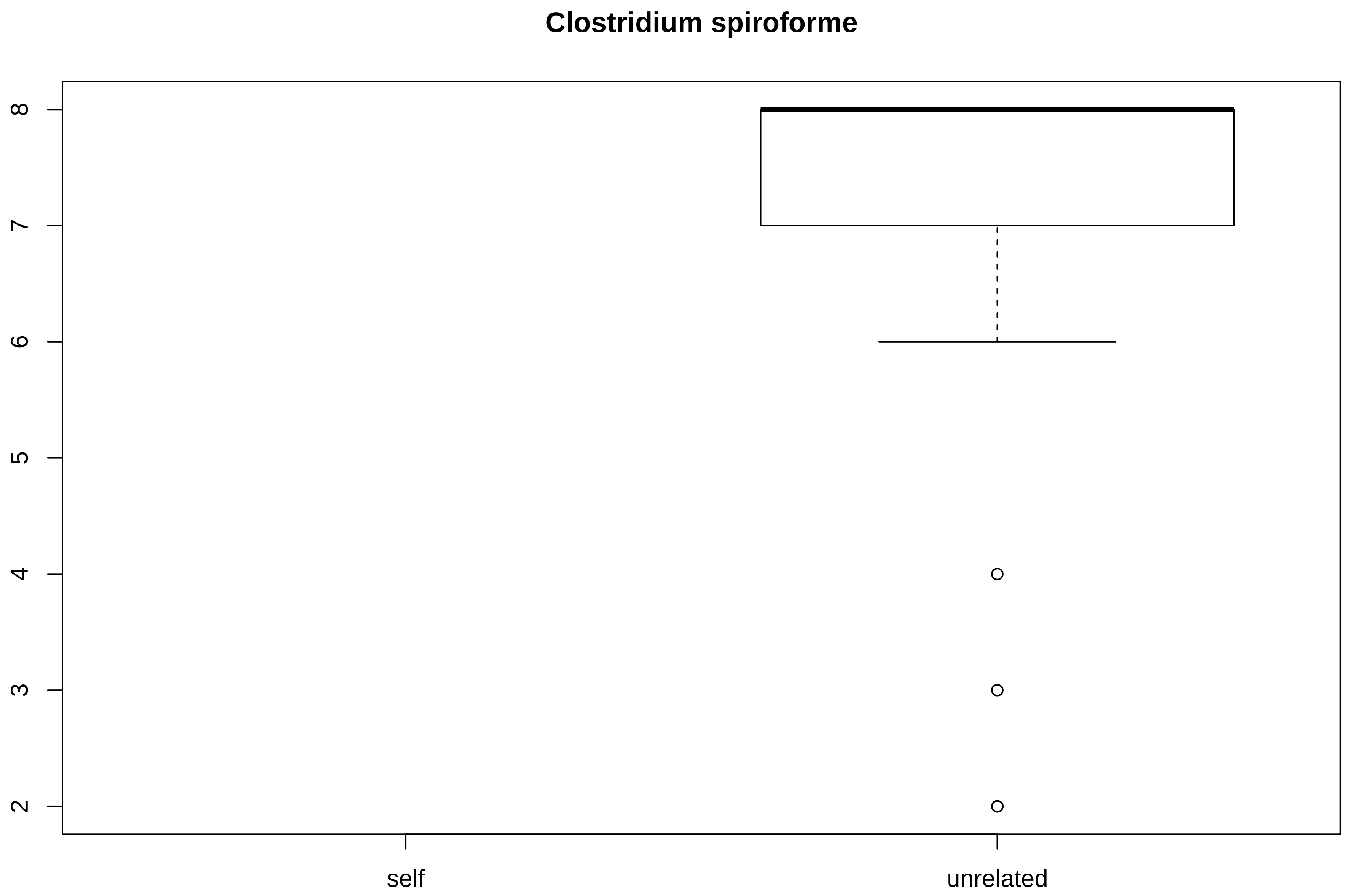
Clostridium spiroforme
8
7
6
5
4
3
2
self
unrelated

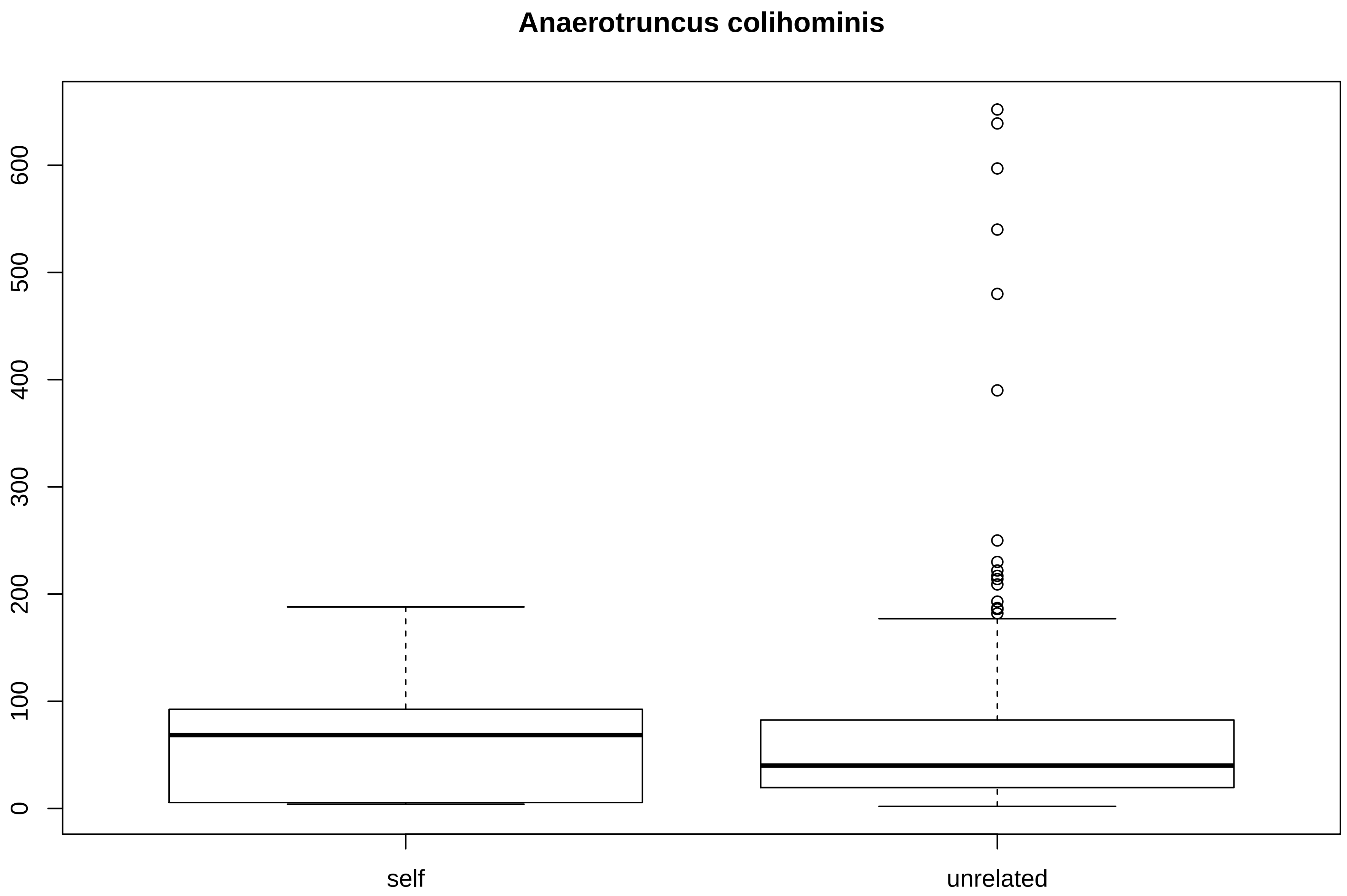
Anaerotruncus colihominis
0
100
200
300
400
500
600
self
unrelated

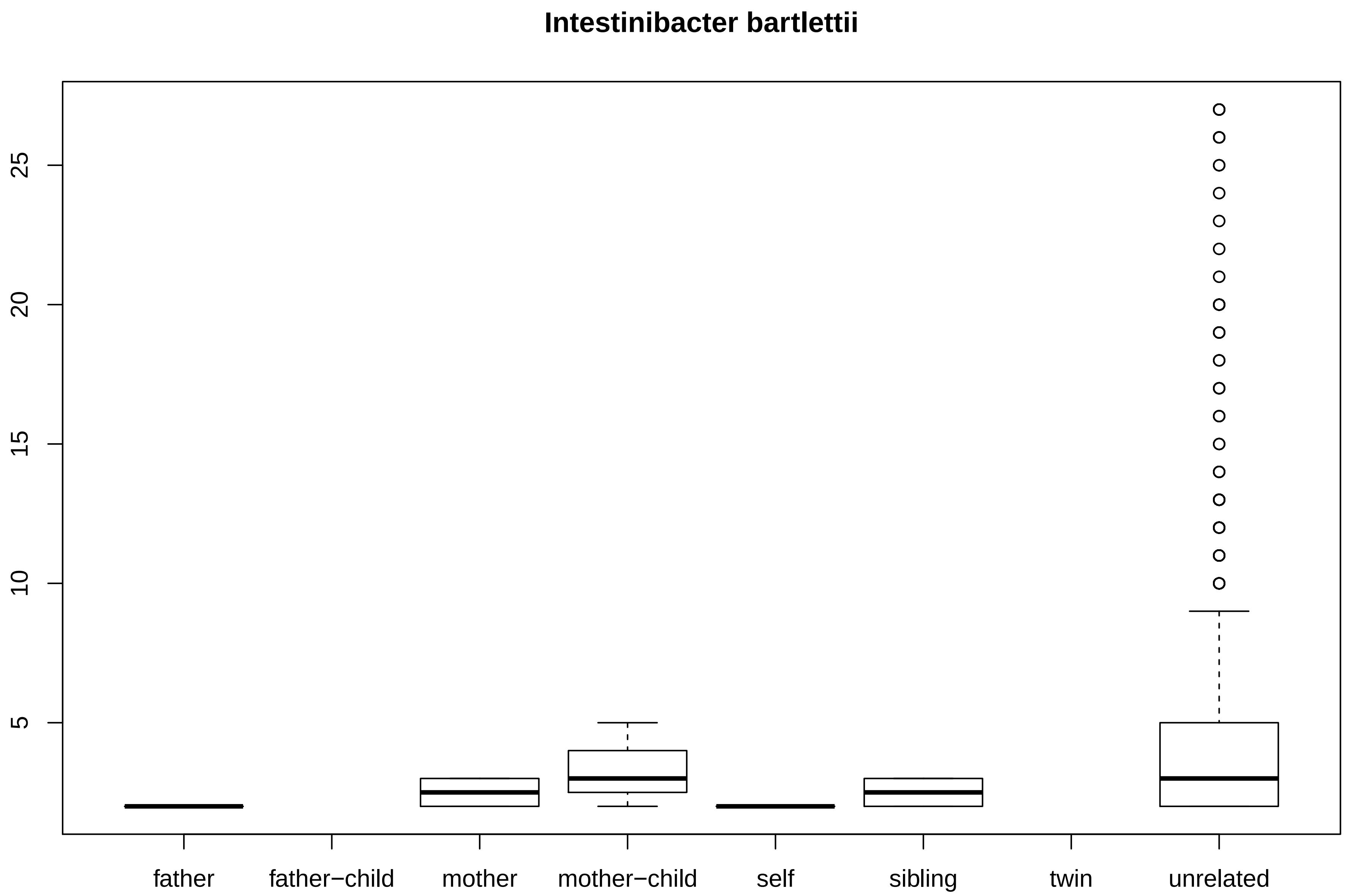
Intestinibacter bartlettii
25
20
15
10
5
father
father−child
mother
mother−child
self
sibling
twin
unrelated

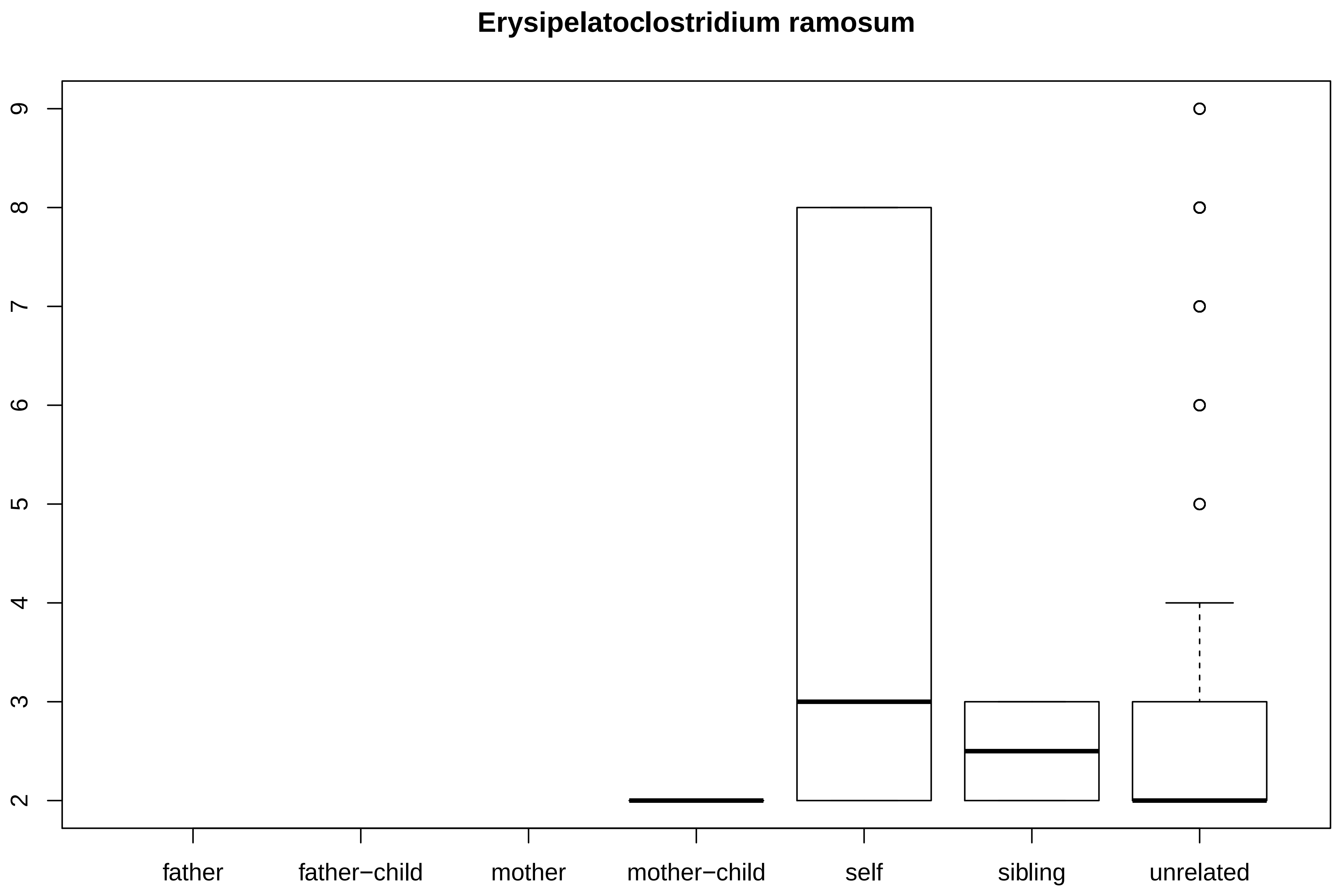

Erysipelatoclostridium ramosum
2
3
4
5
6
7
8
9
father
father−child
mother
mother−child
self
sibling
unrelated

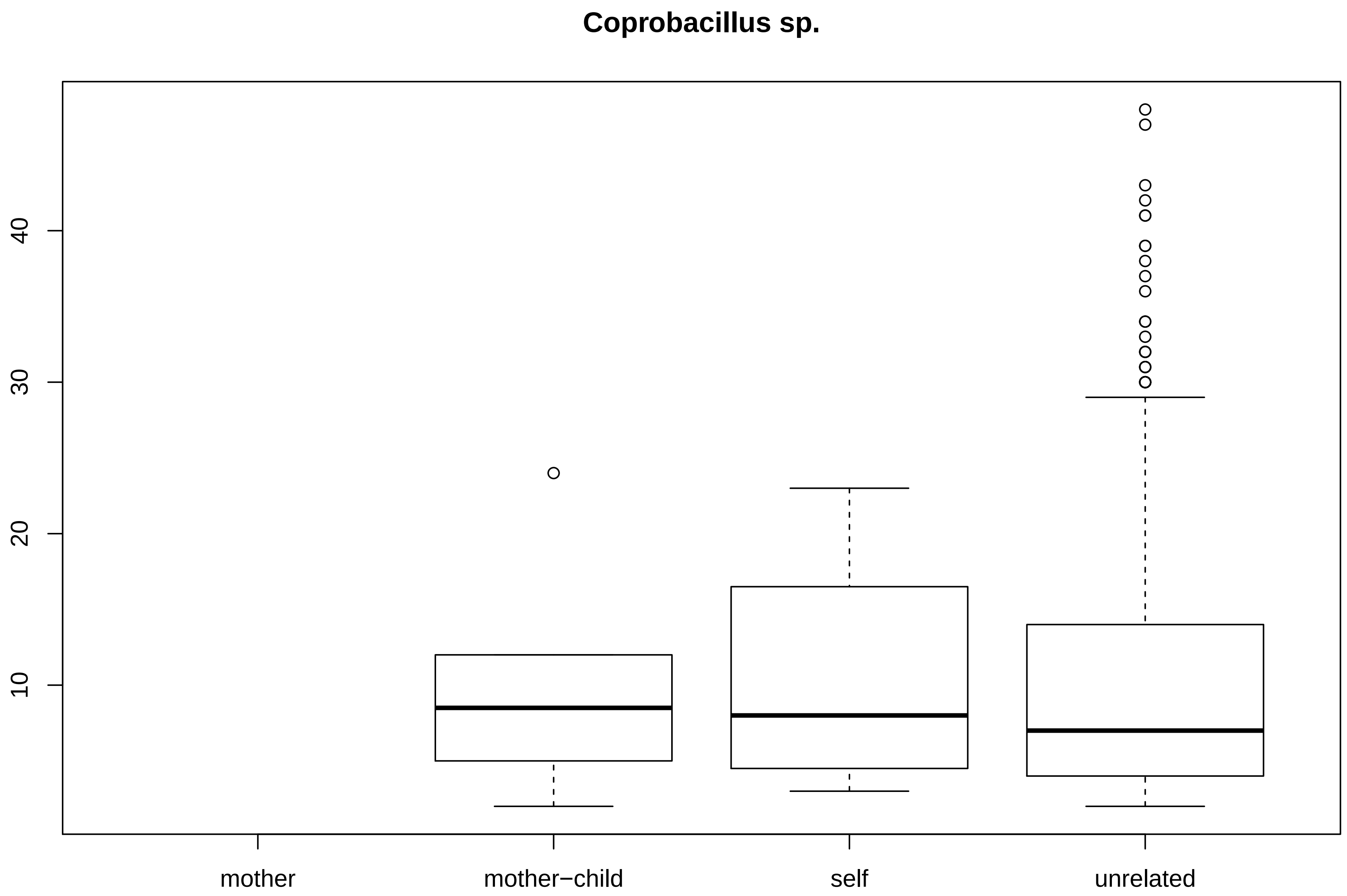
Coprobacillus sp.
10
20
30
40
mother
mother−child
self
unrelated

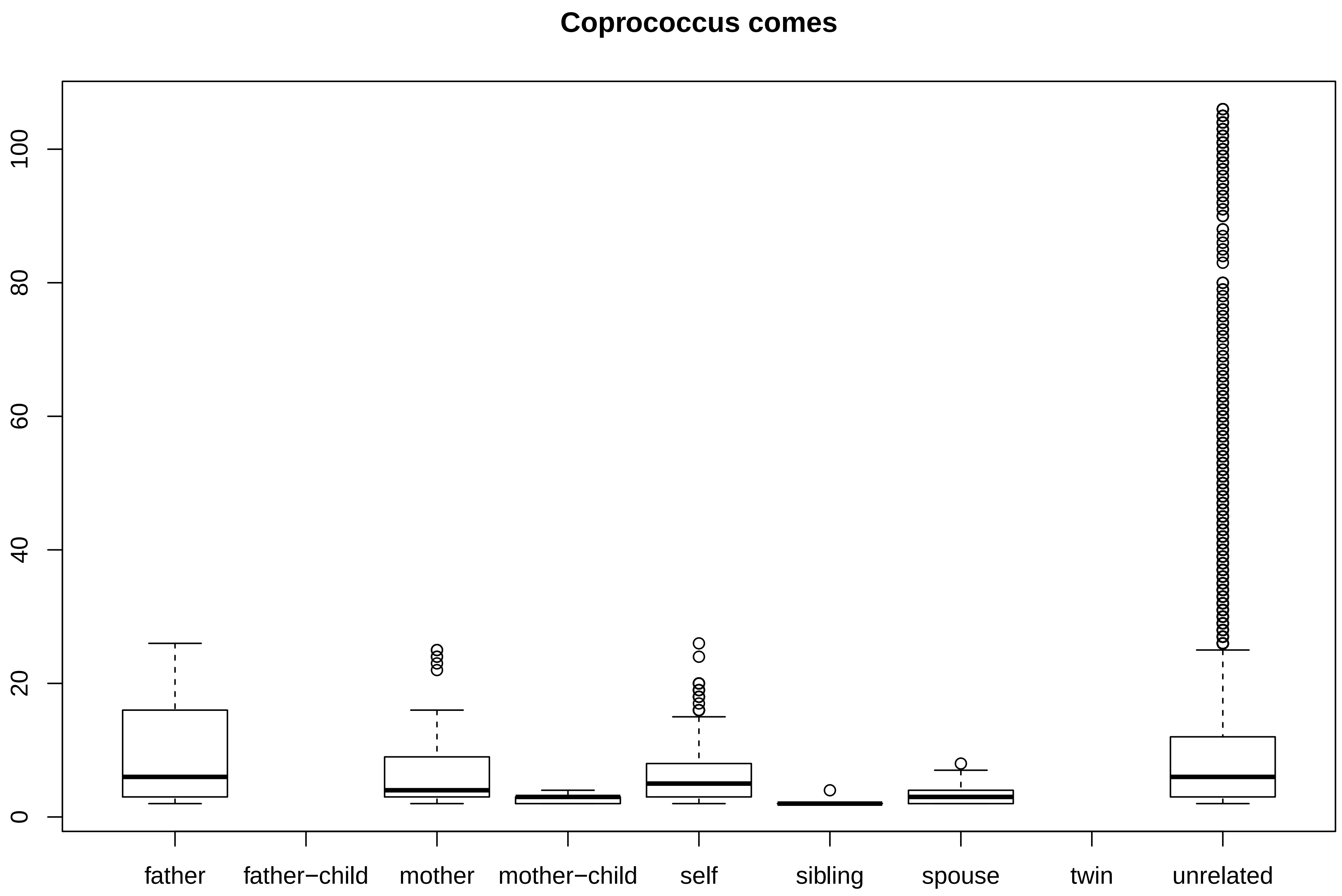
Coprococcus comes
0
20
40
60
80
100
father
father−child
mother
mother−child
self
sibling
spouse
twin
unrelated

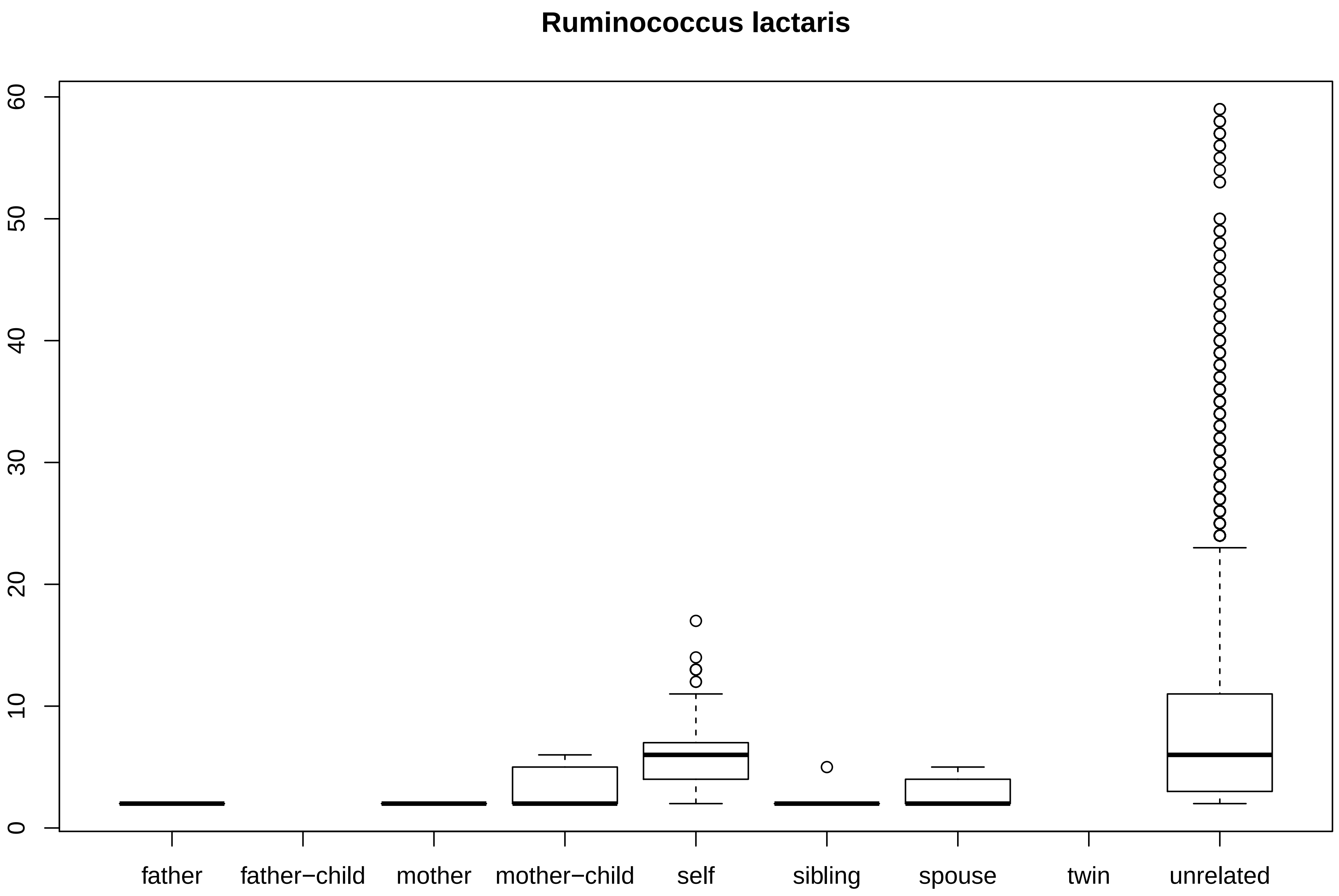

Ruminococcus lactaris
0
10
20
30
40
50
60
father
father−child
mother
mother−child
self
sibling
spouse
twin
unrelated

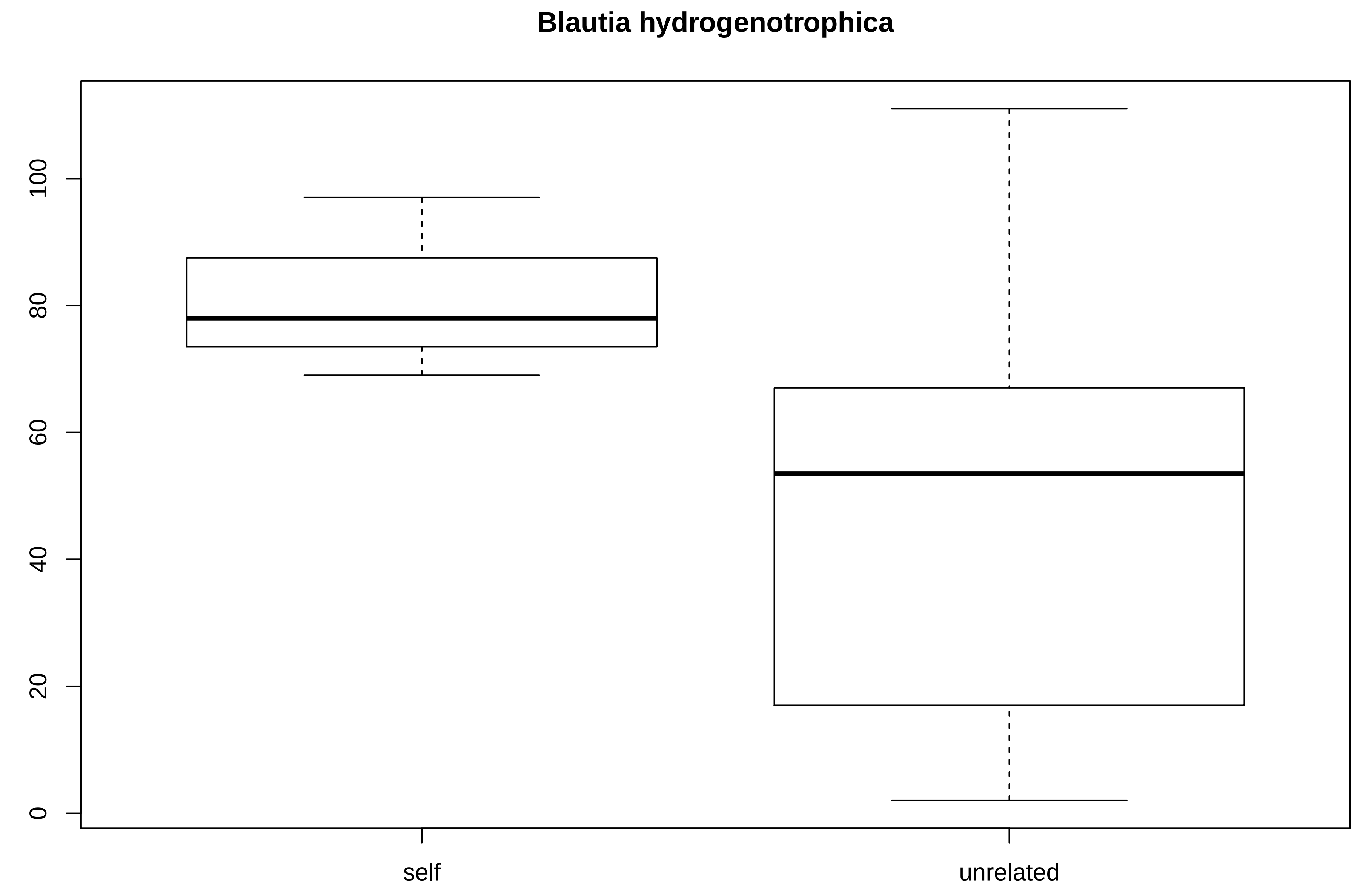
Blautia hydrogenotrophica
100
80
60
40
20
0
self
unrelated

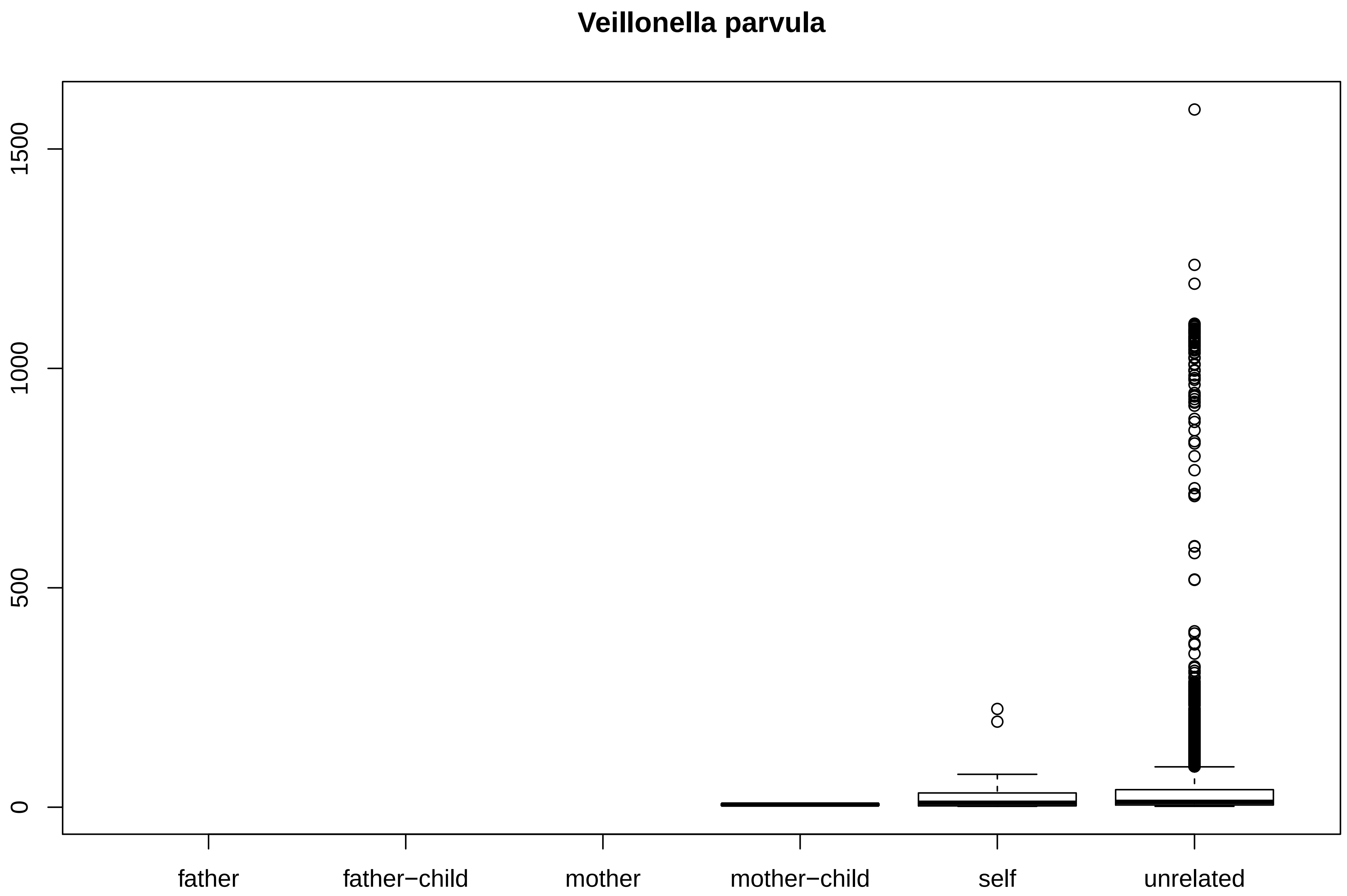

Veillonella parvula
1500
1000
500
0
father
father−child
mother
mother−child
self
unrelated

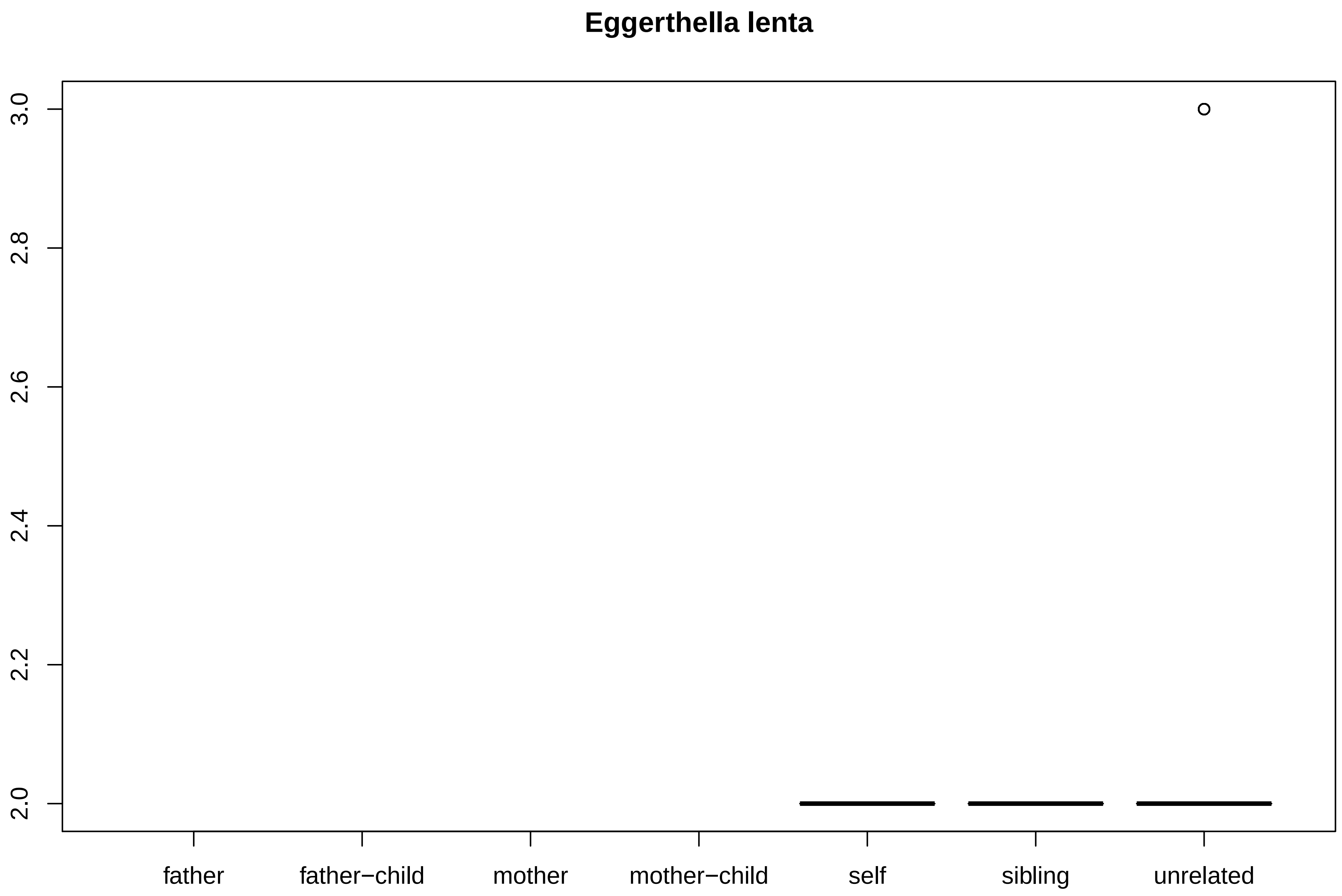

Eggerthella lenta
3.0
2.8
2.6
2.4
2.2
2.0
father
father−child
mother
mother−child
self
sibling
unrelated

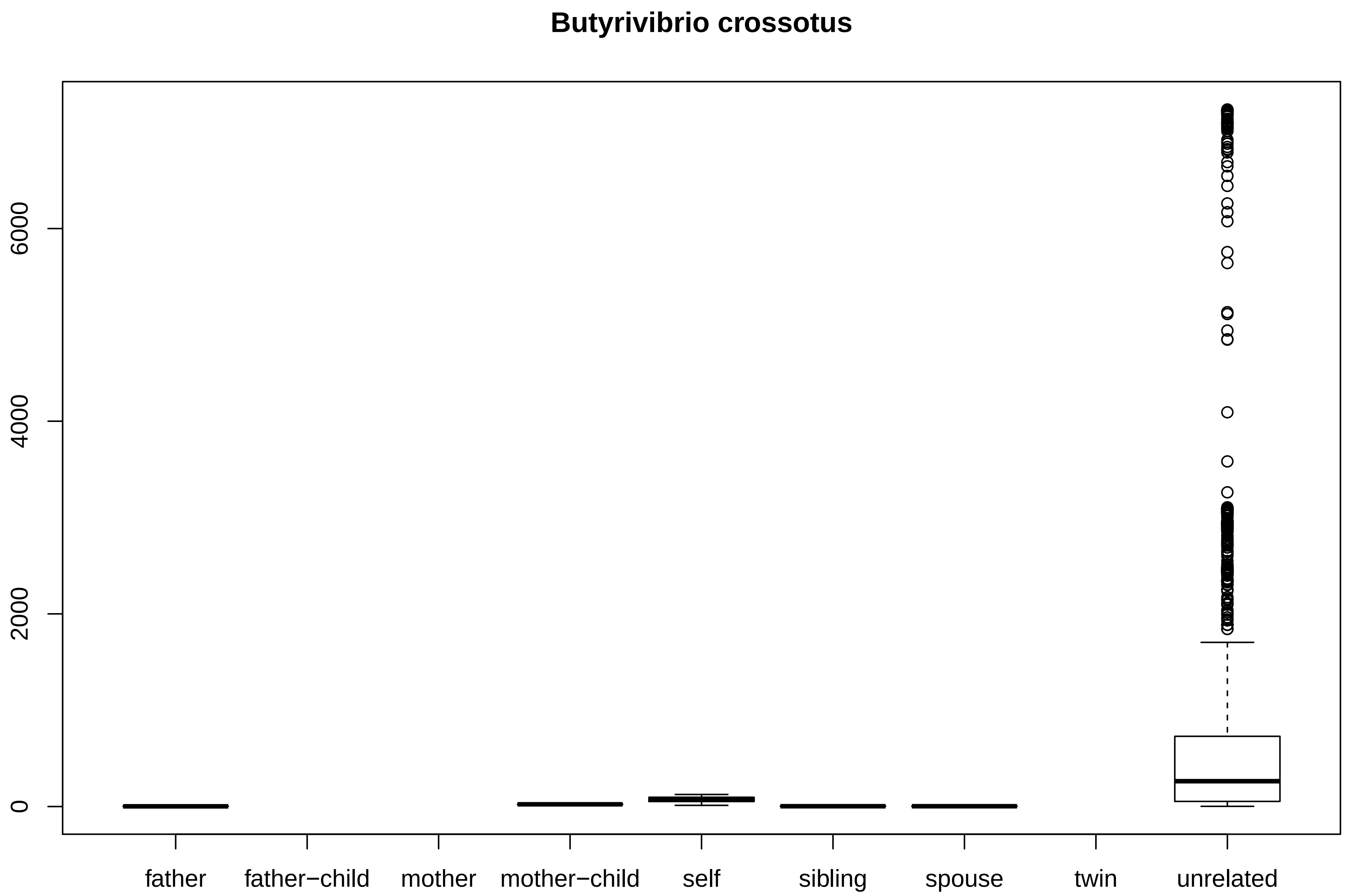
Butyrivibrio crossotus
0
2000
4000
6000
father
father−child
mother
mother−child
self
sibling
spouse
twin
unrelated

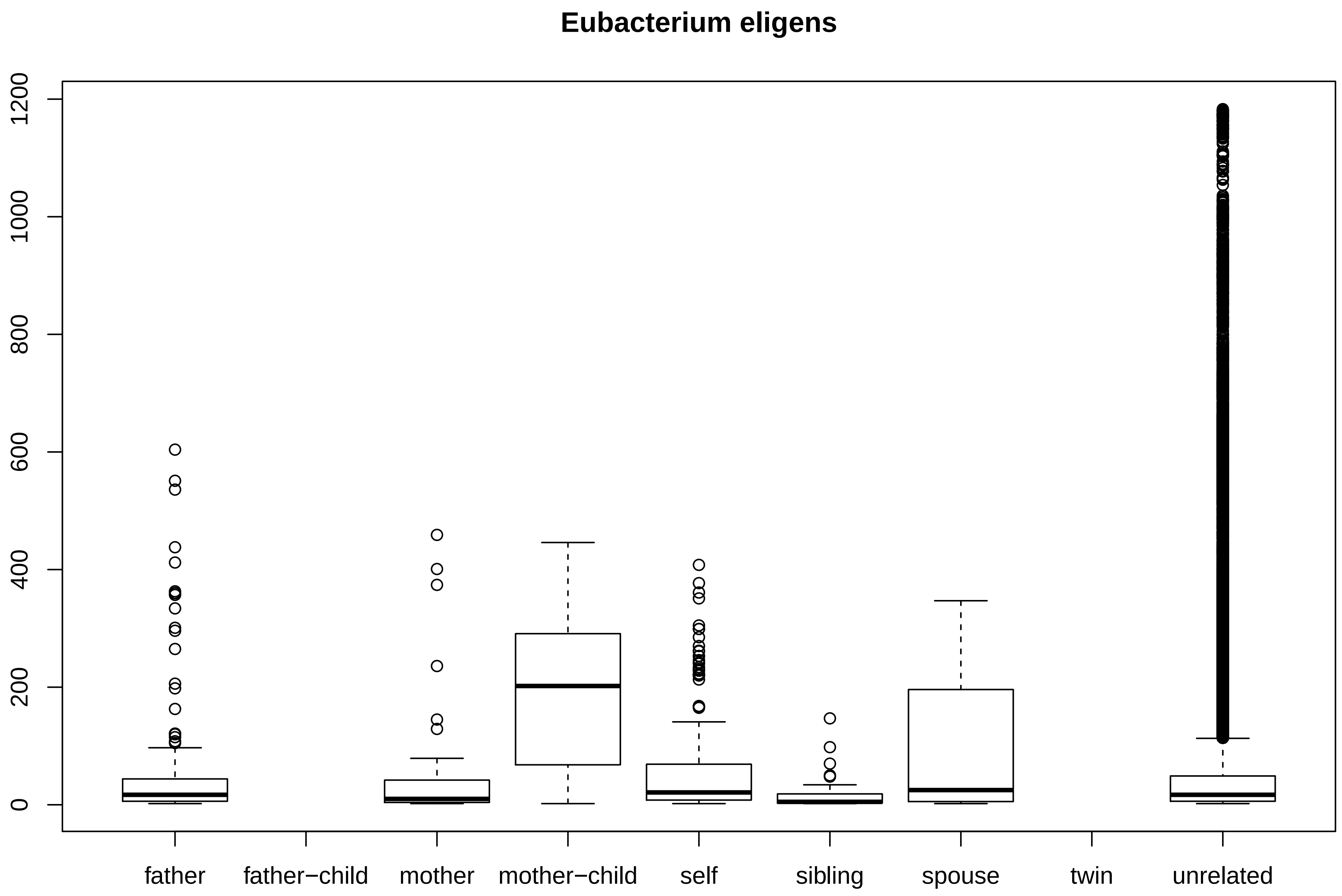
Eubacterium eligens
0
200
400
600
800
1000
1200
father
father−child
mother
mother−child
self
sibling
spouse
twin
unrelated

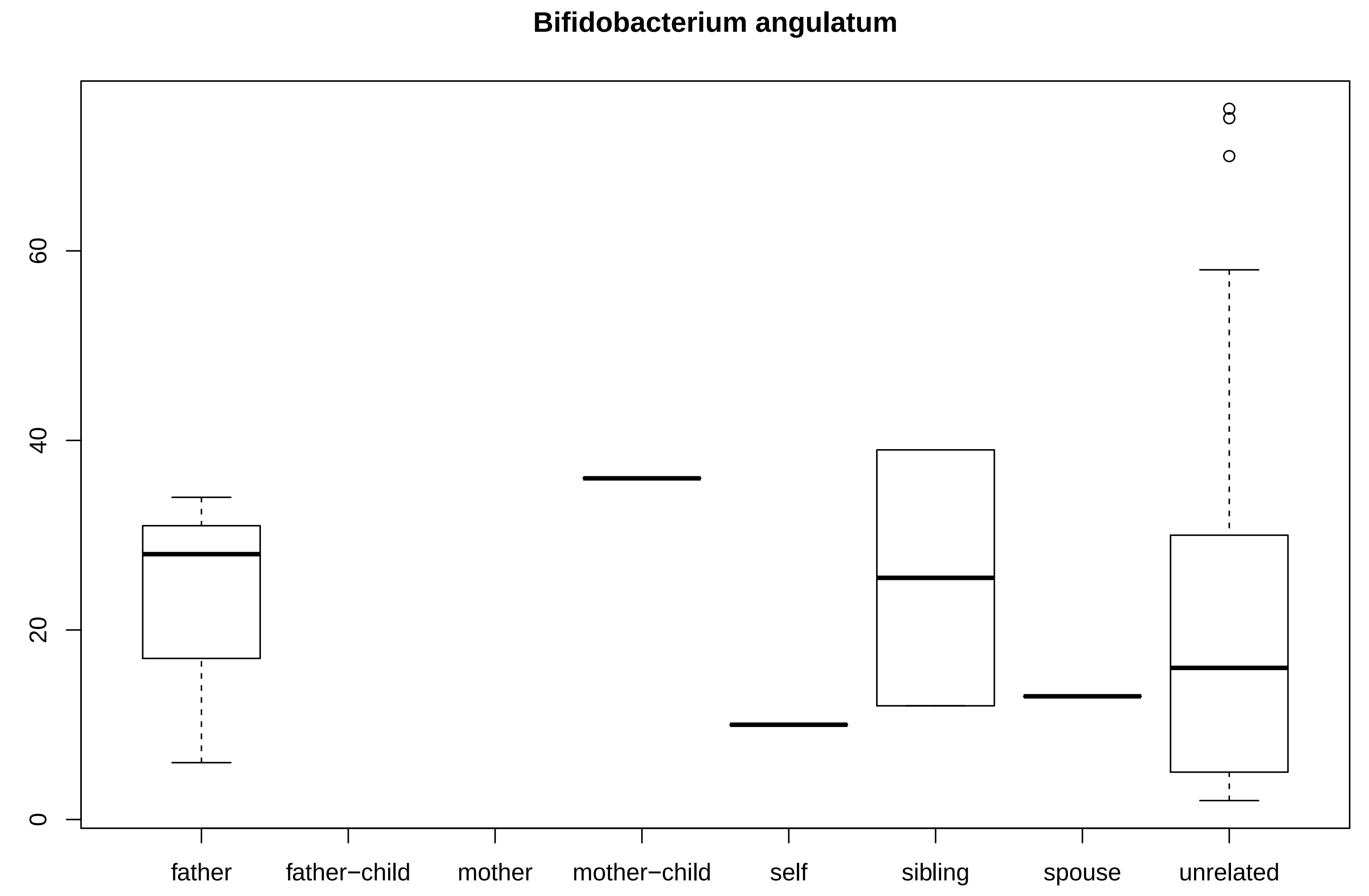
Bifidobacterium angulatum
0
20
40
60
father
father−child
mother
mother−child
self
sibling
spouse
unrelated

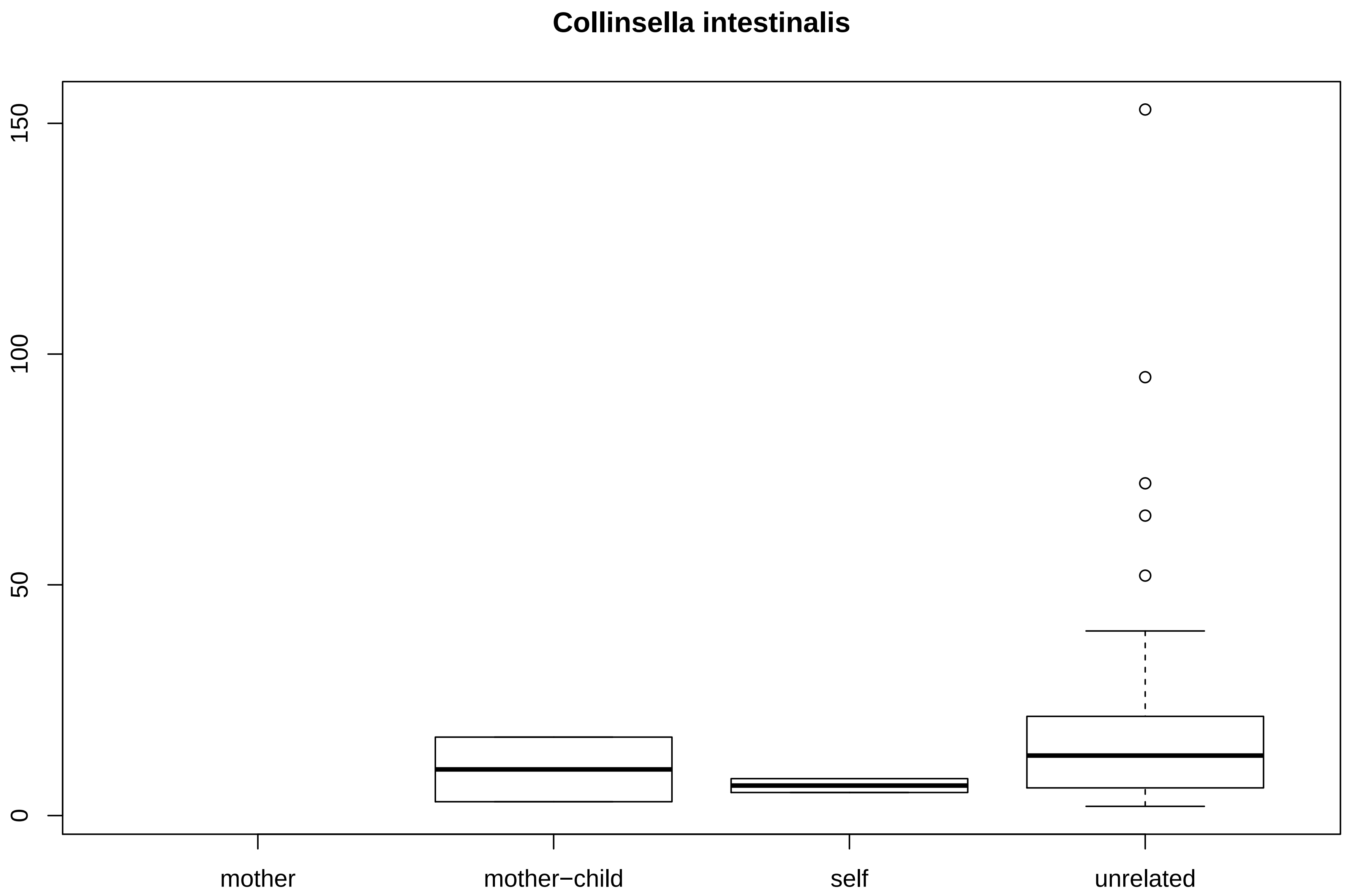
Collinsella intestinalis
150
100
50
0
mother
mother−child
self
unrelated

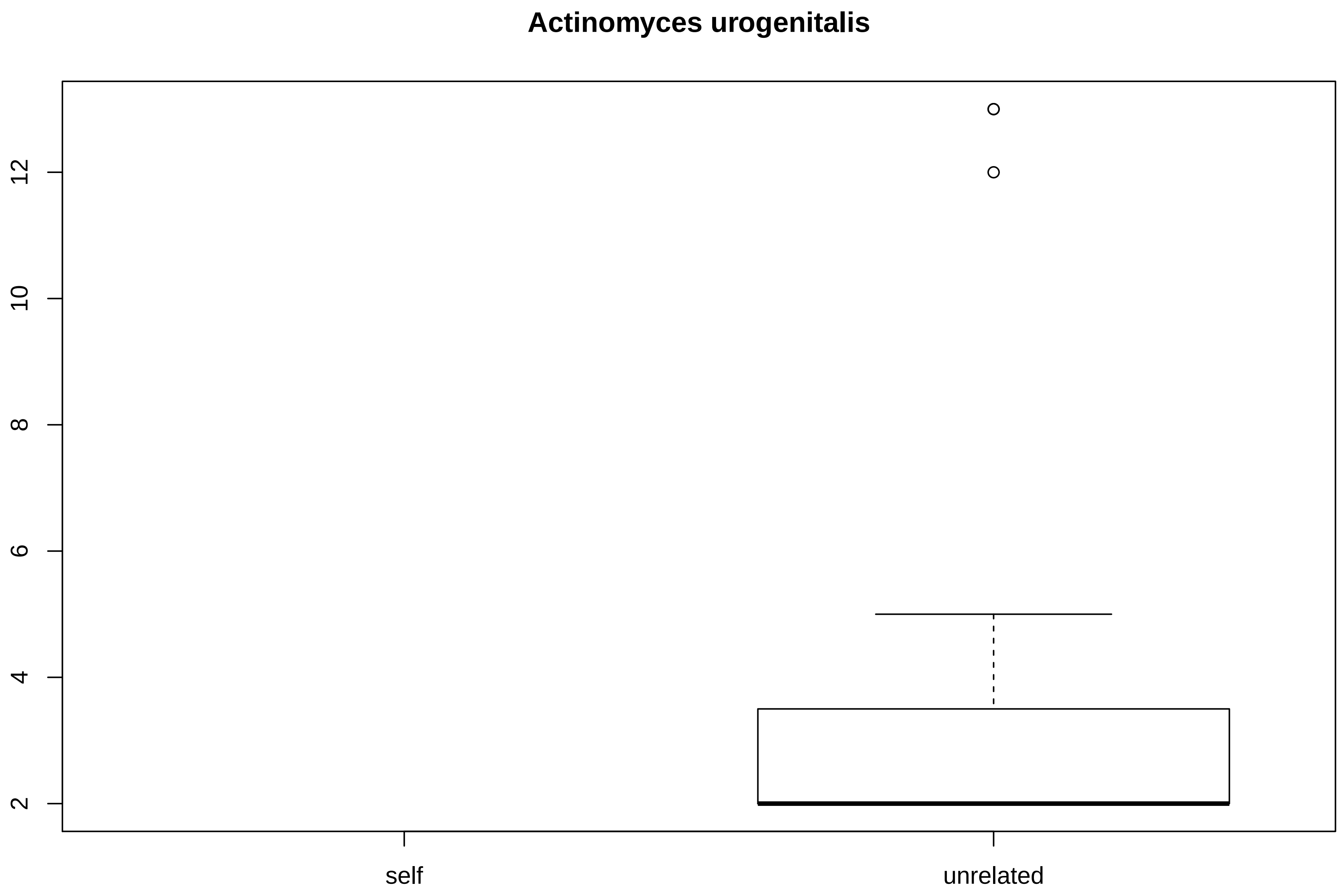

Actinomyces urogenitalis
12
10
8
6
4
2
self
unrelated

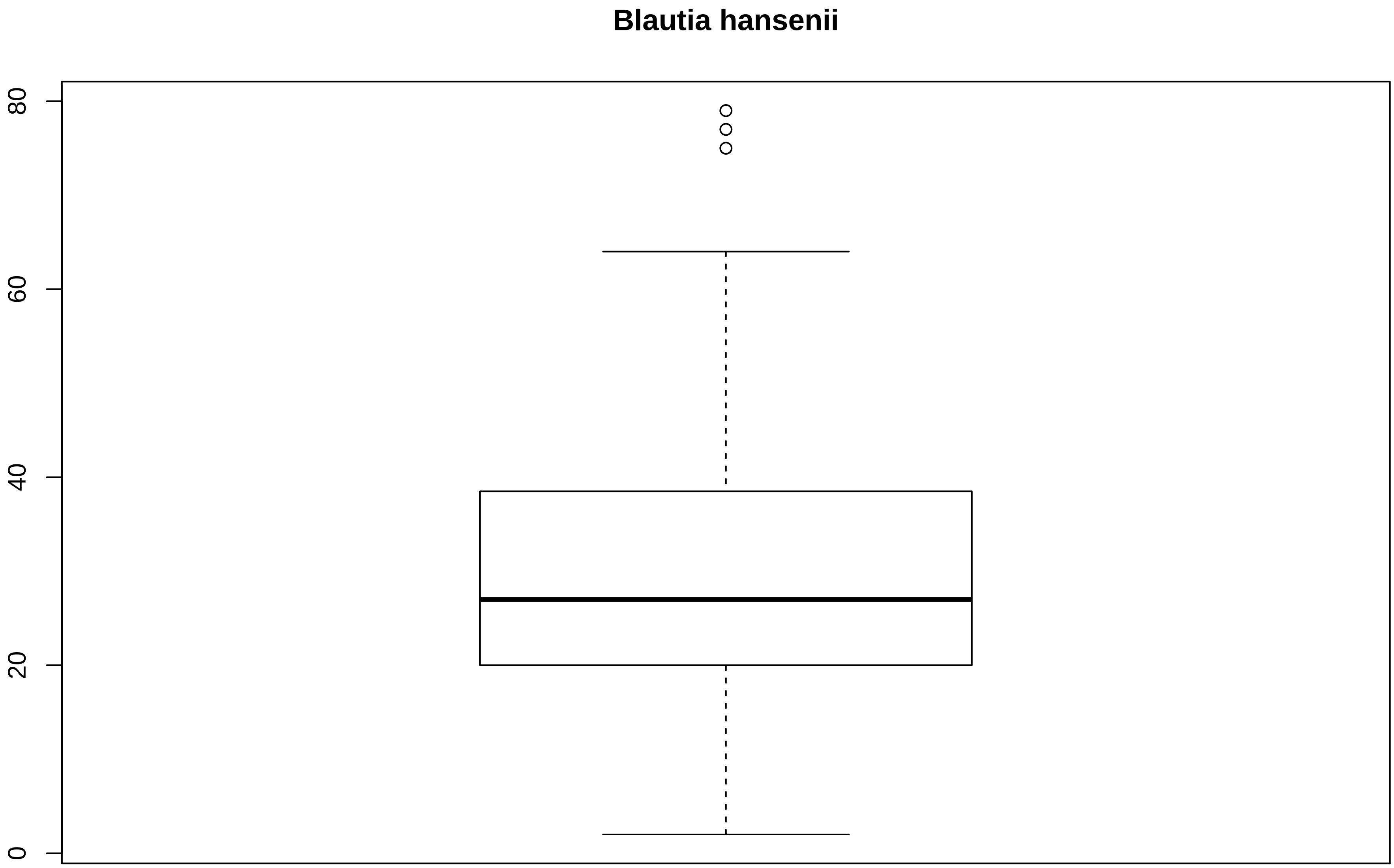
Blautia hansenii
0
20
40
60
80

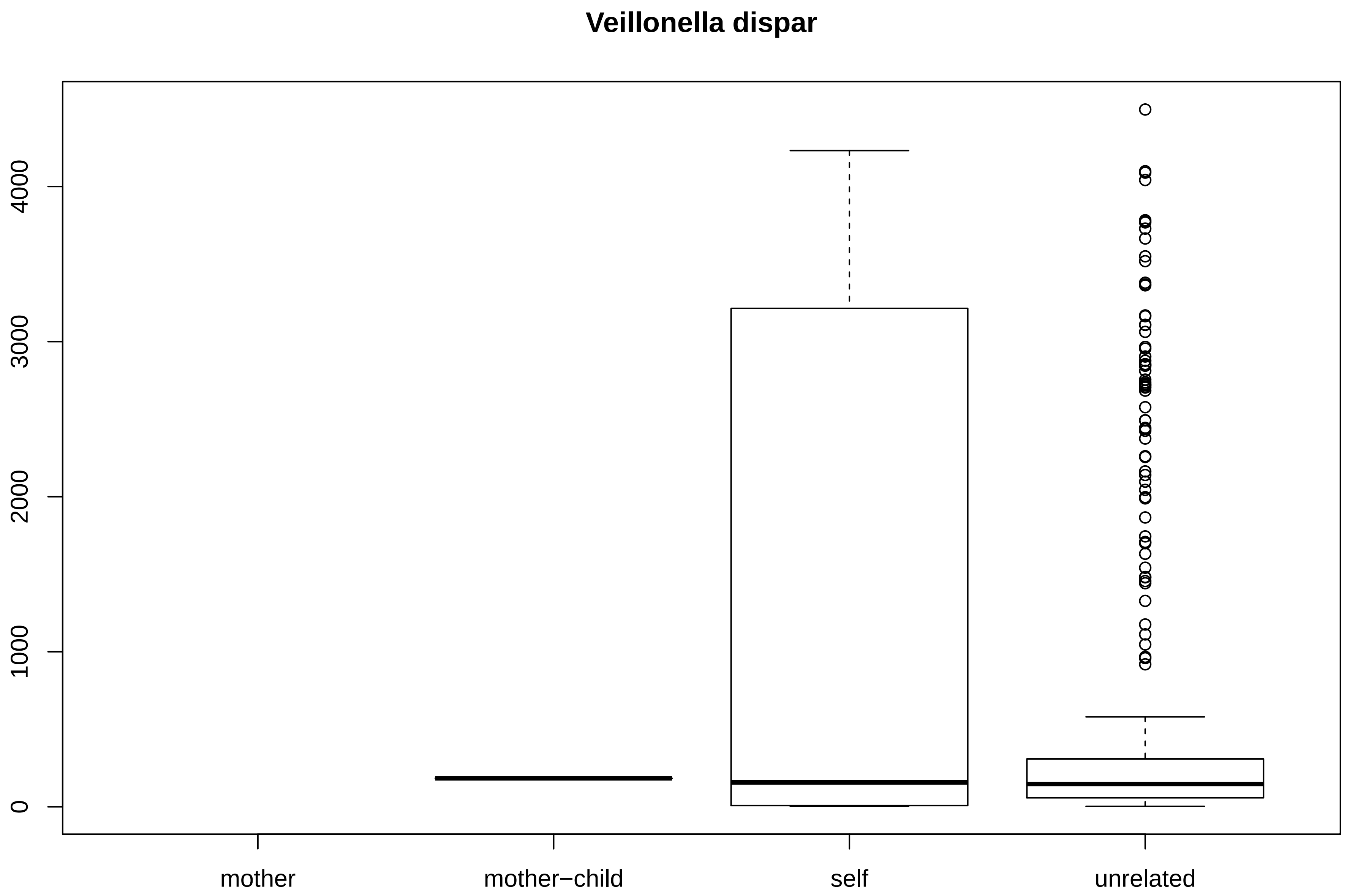

Veillonella dispar
0
1000
2000
3000
4000
mother
mother−child
self
unrelated

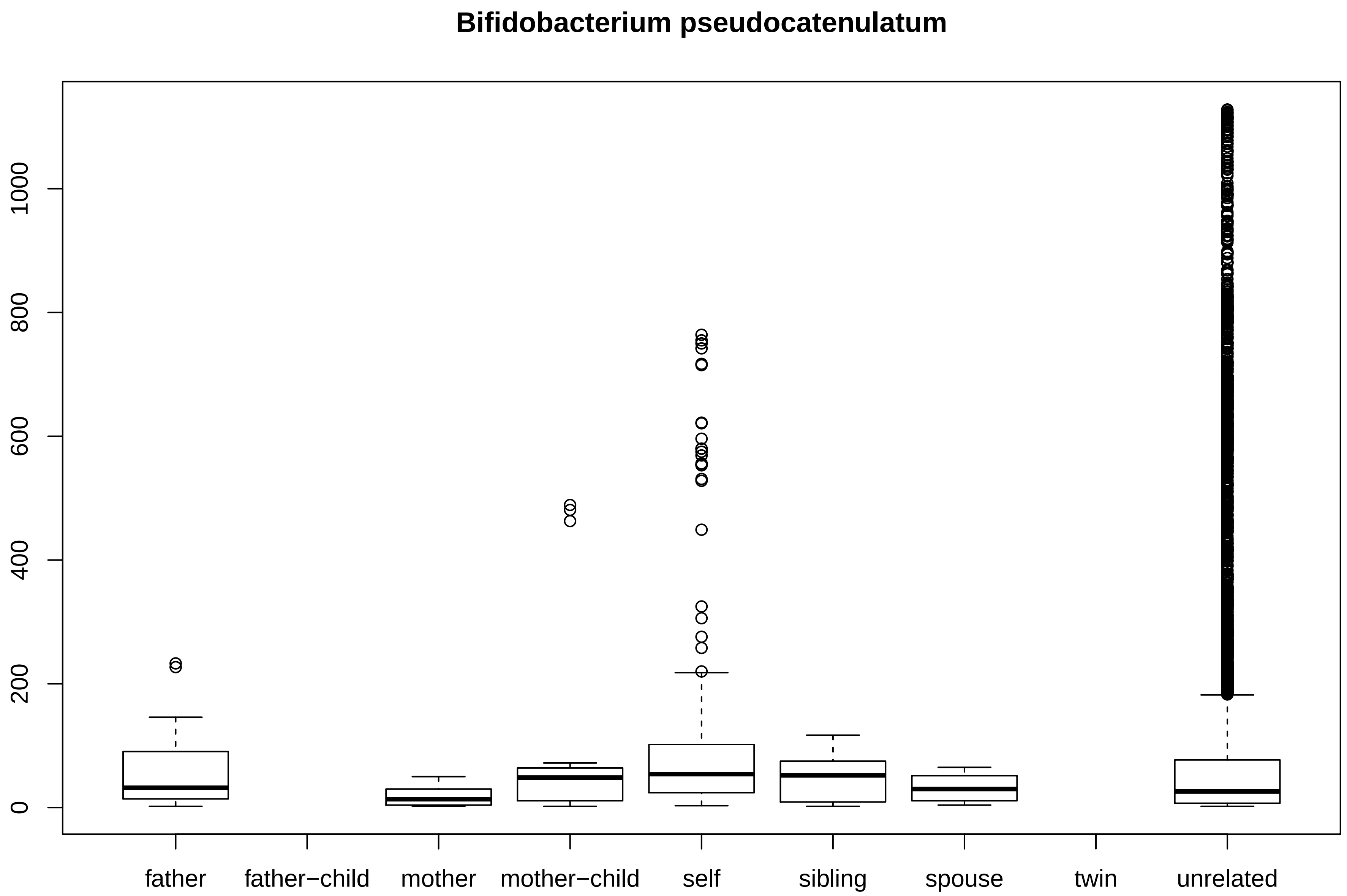
Bifidobacterium pseudocatenulatum
0
200
400
600
800
1000
father
father−child
mother
mother−child
self
sibling
spouse
twin
unrelated

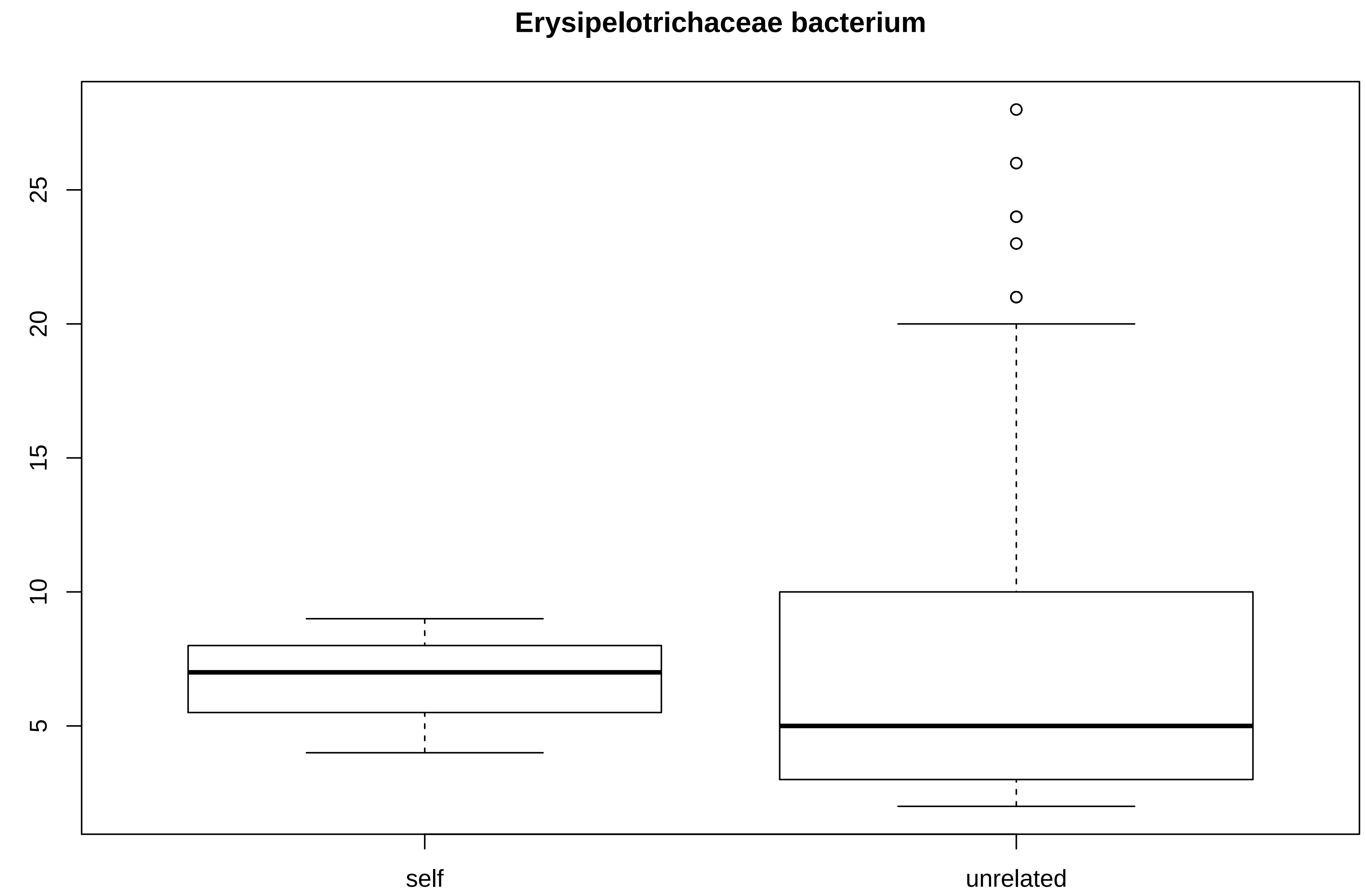
Erysipelotrichaceae bacterium
25
20
15
10
5
self
unrelated

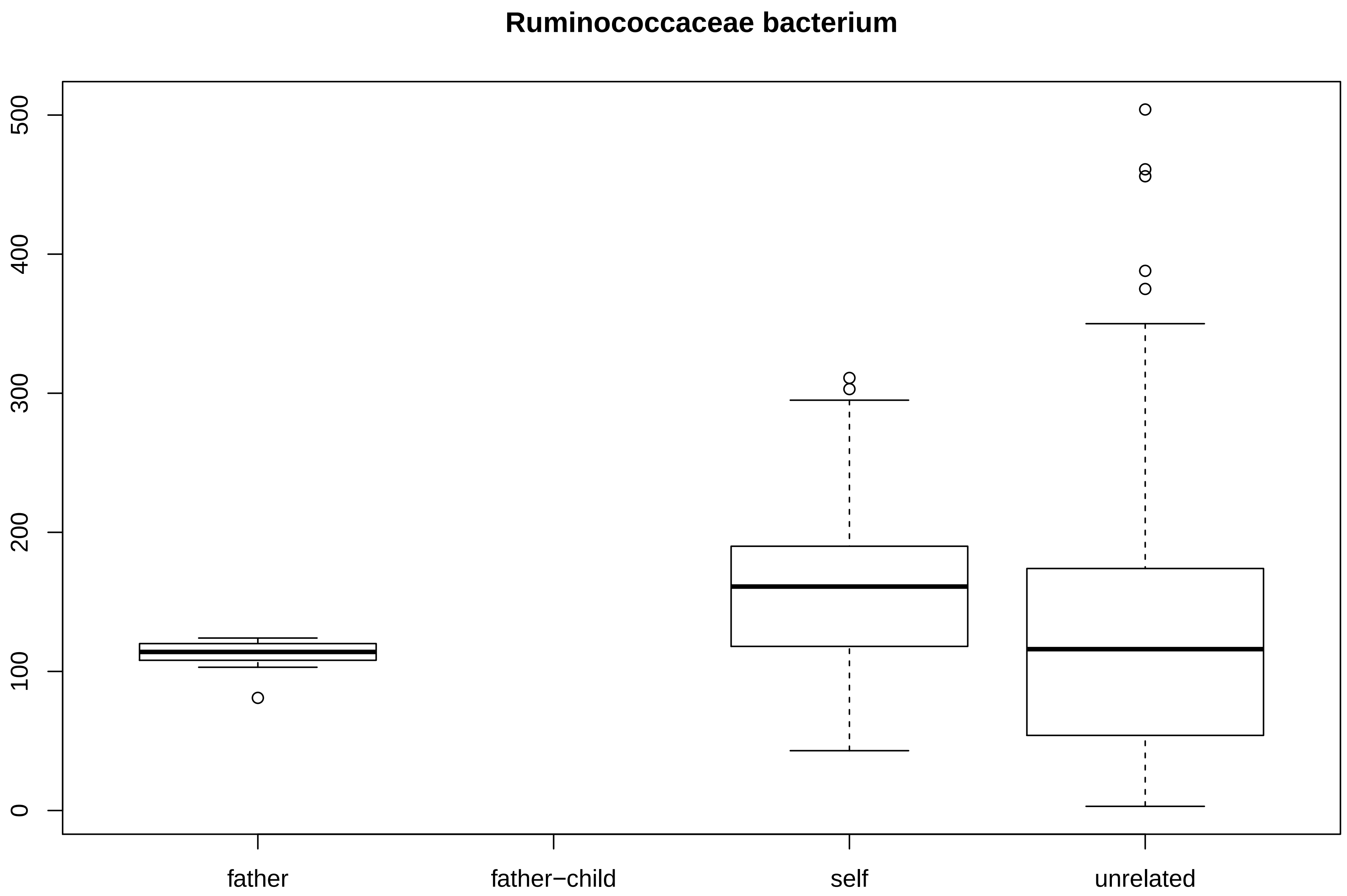

Ruminococcaceae bacterium
0
100
200
300
400
500
father
father−child
self
unrelated

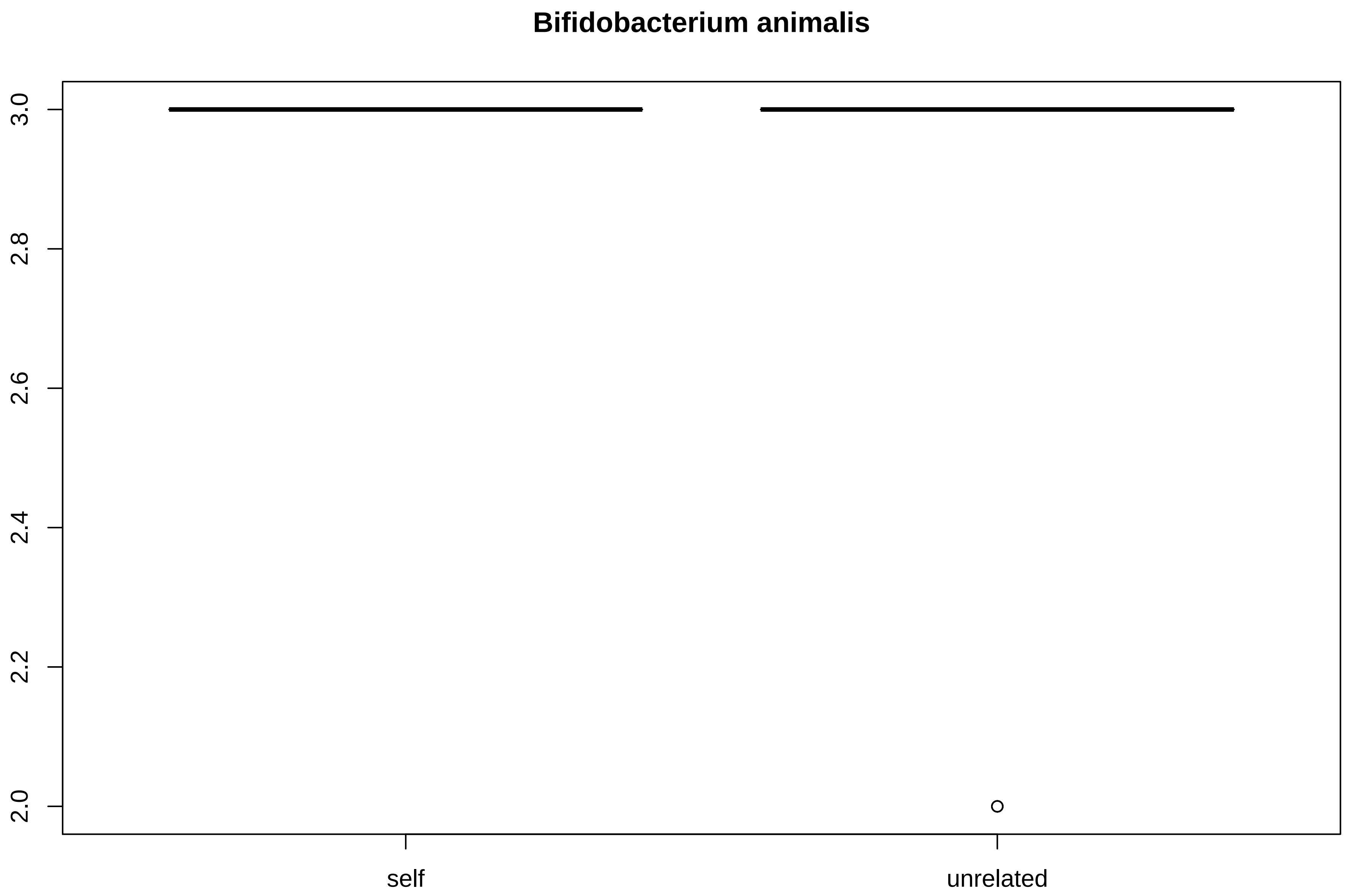
Bifidobacterium animalis
3.0
2.8
2.6
2.4
2.2
2.0
self
unrelated

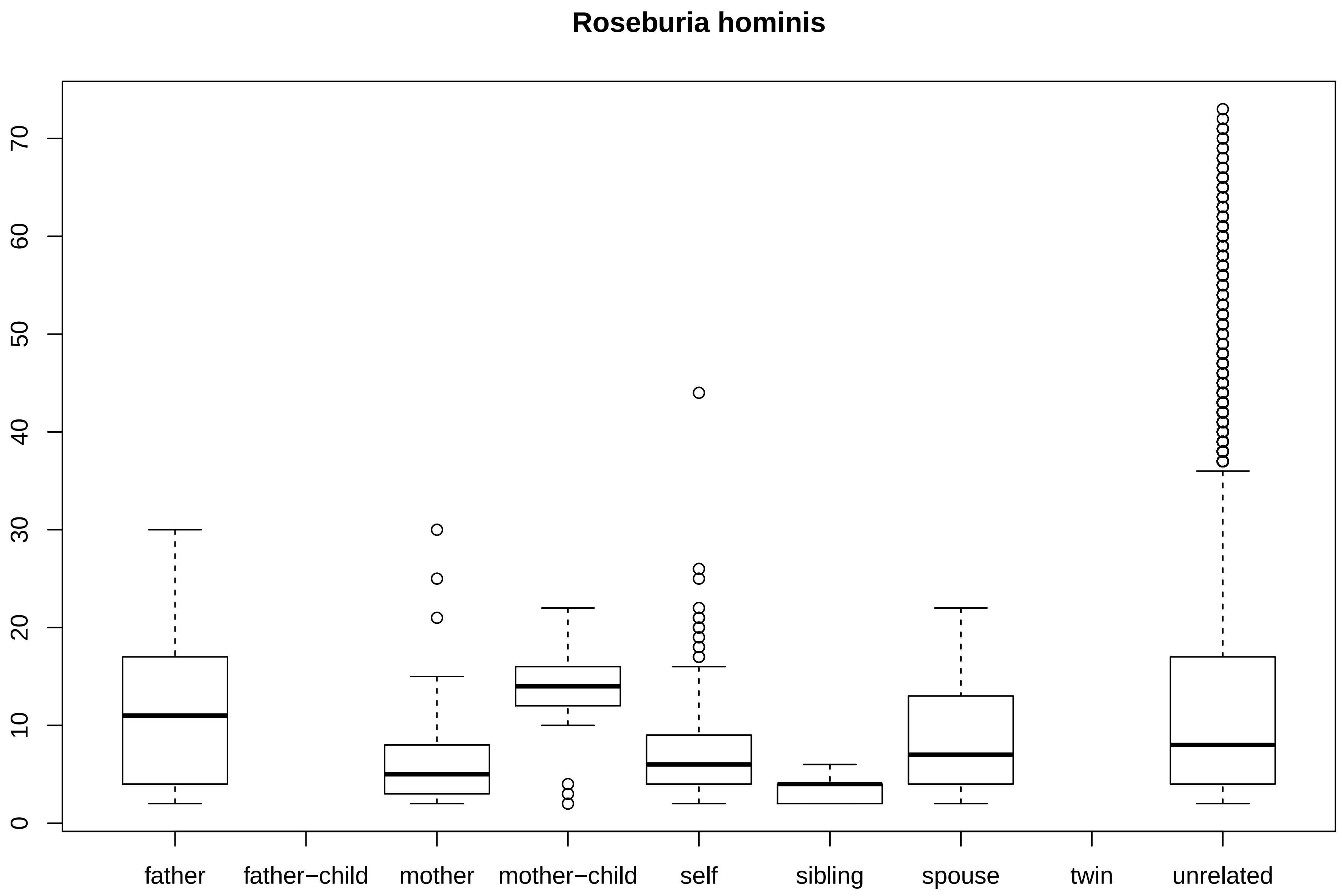
Roseburia hominis
0
10
20
30
40
50
60
70
father
father−child
mother
mother−child
self
sibling
spouse
twin
unrelated

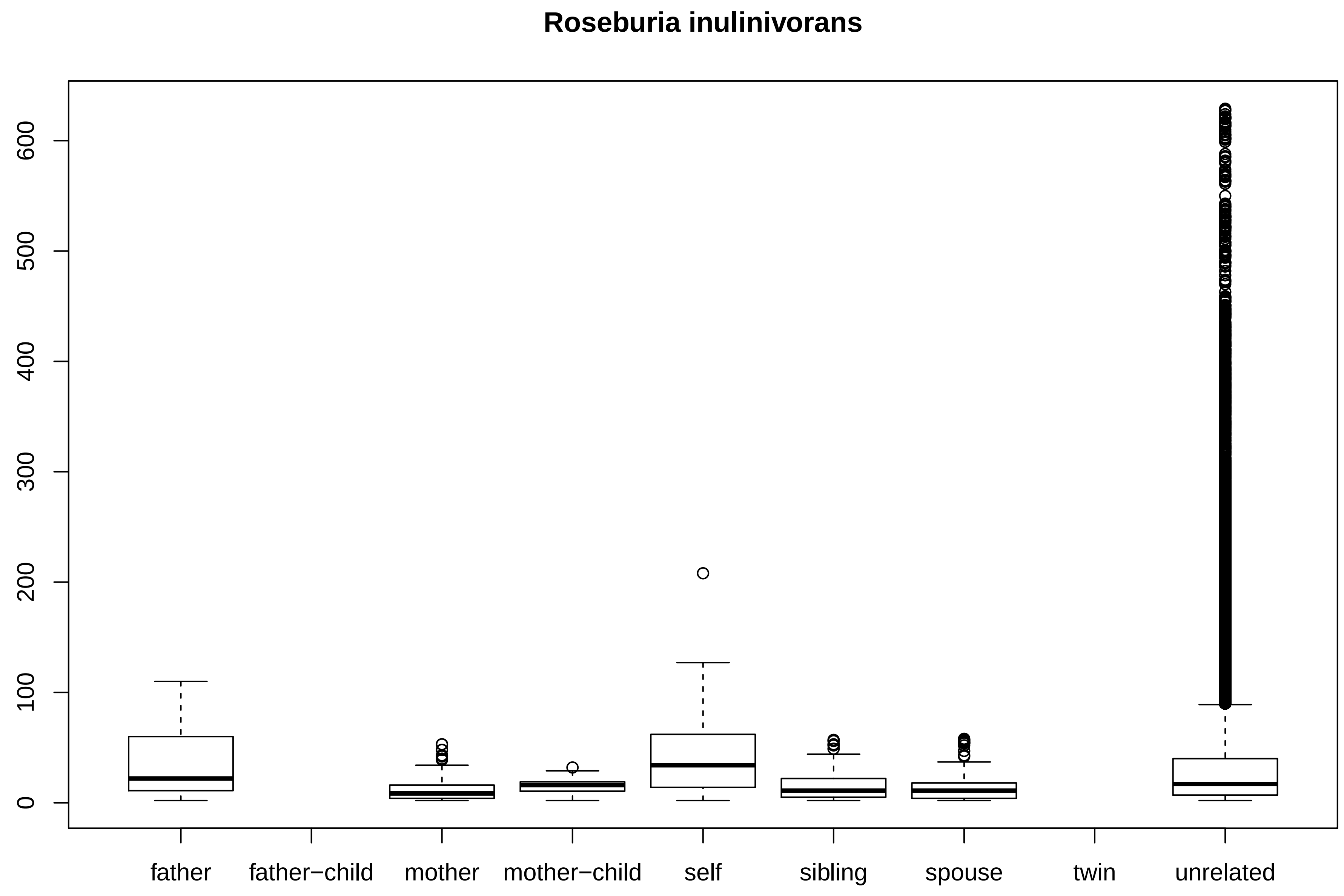
Roseburia inulinivorans
0
100
200
300
400
500
600
father
father−child
mother
mother−child
self
sibling
spouse
twin
unrelated

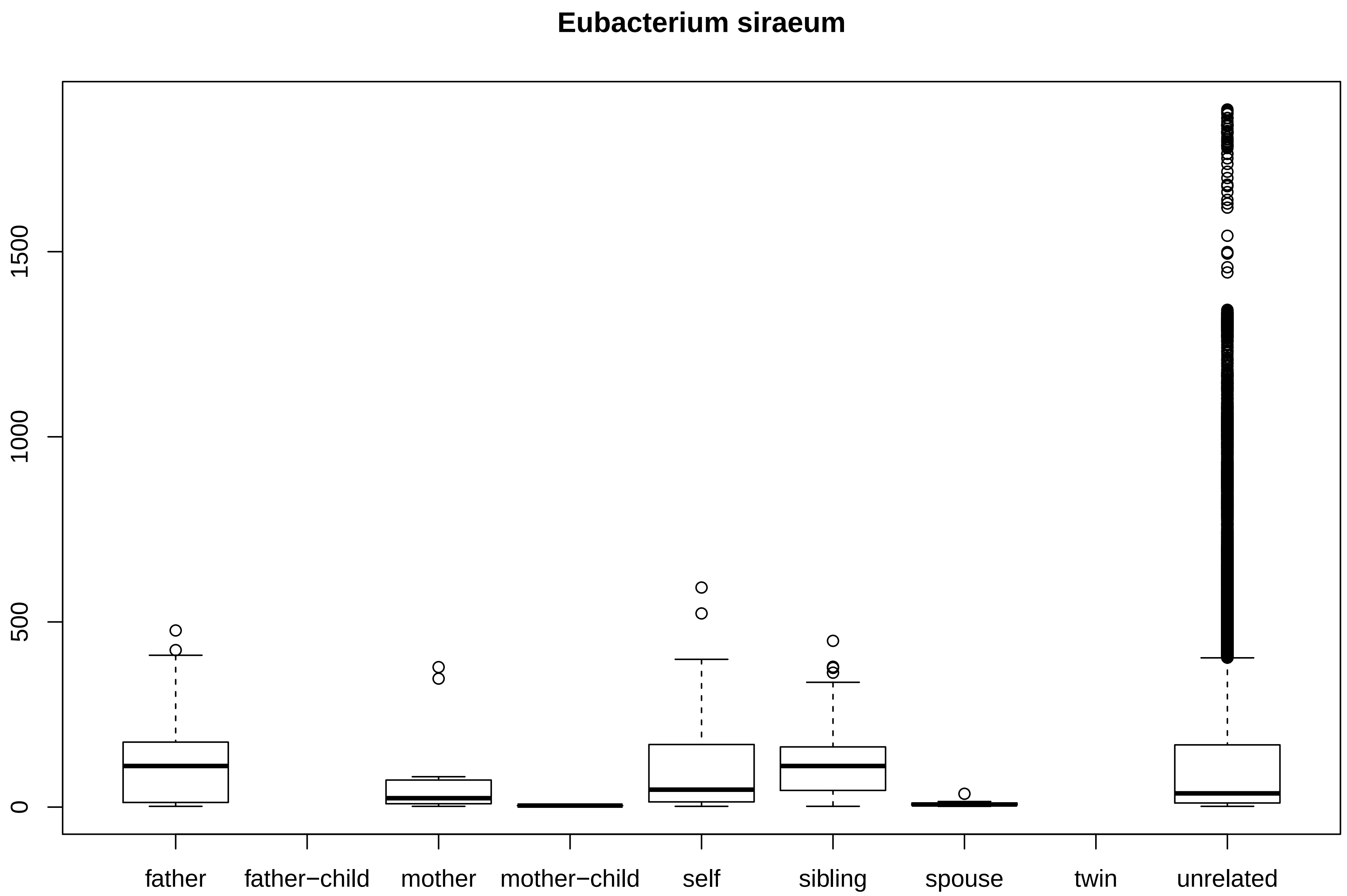
Eubacterium siraeum
0
500
1000
1500
father
father−child
mother
mother−child
self
sibling
spouse
twin
unrelated

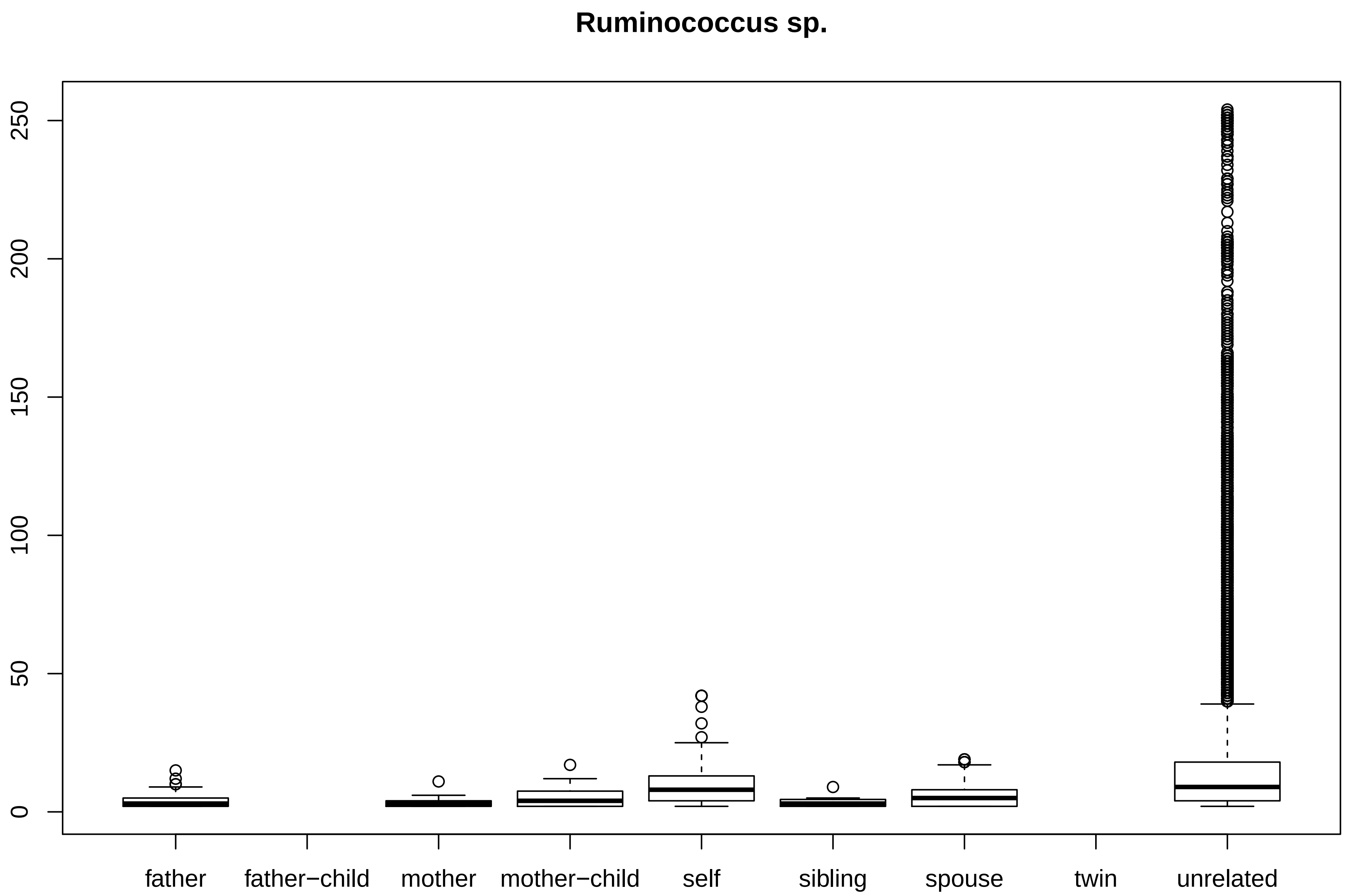

Ruminococcus sp.
0
50
100
150
200
250
father
father−child
mother
mother−child
self
sibling
spouse
twin
unrelated

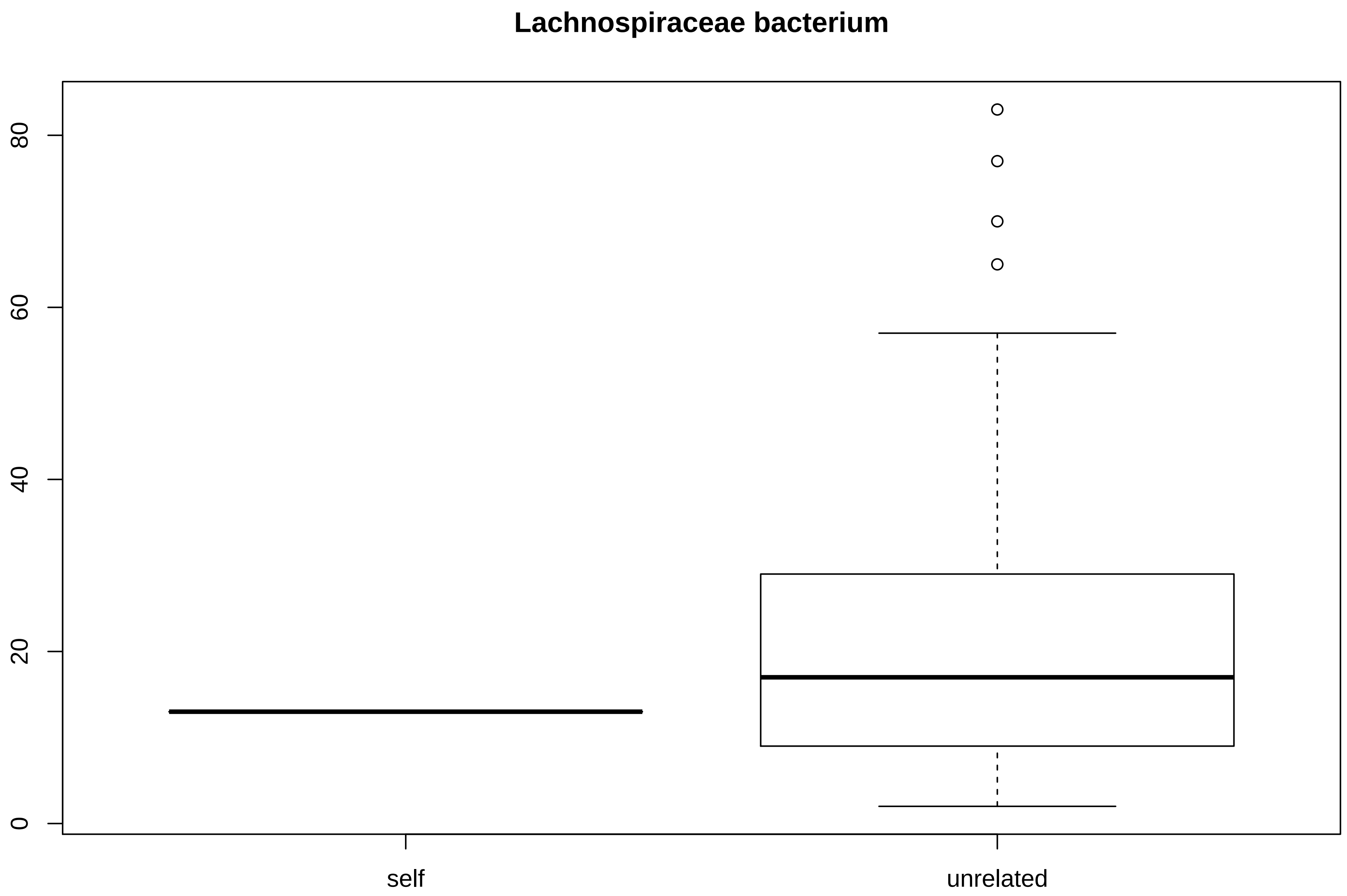
Lachnospiraceae bacterium
0
20
40
60
80
self
unrelated

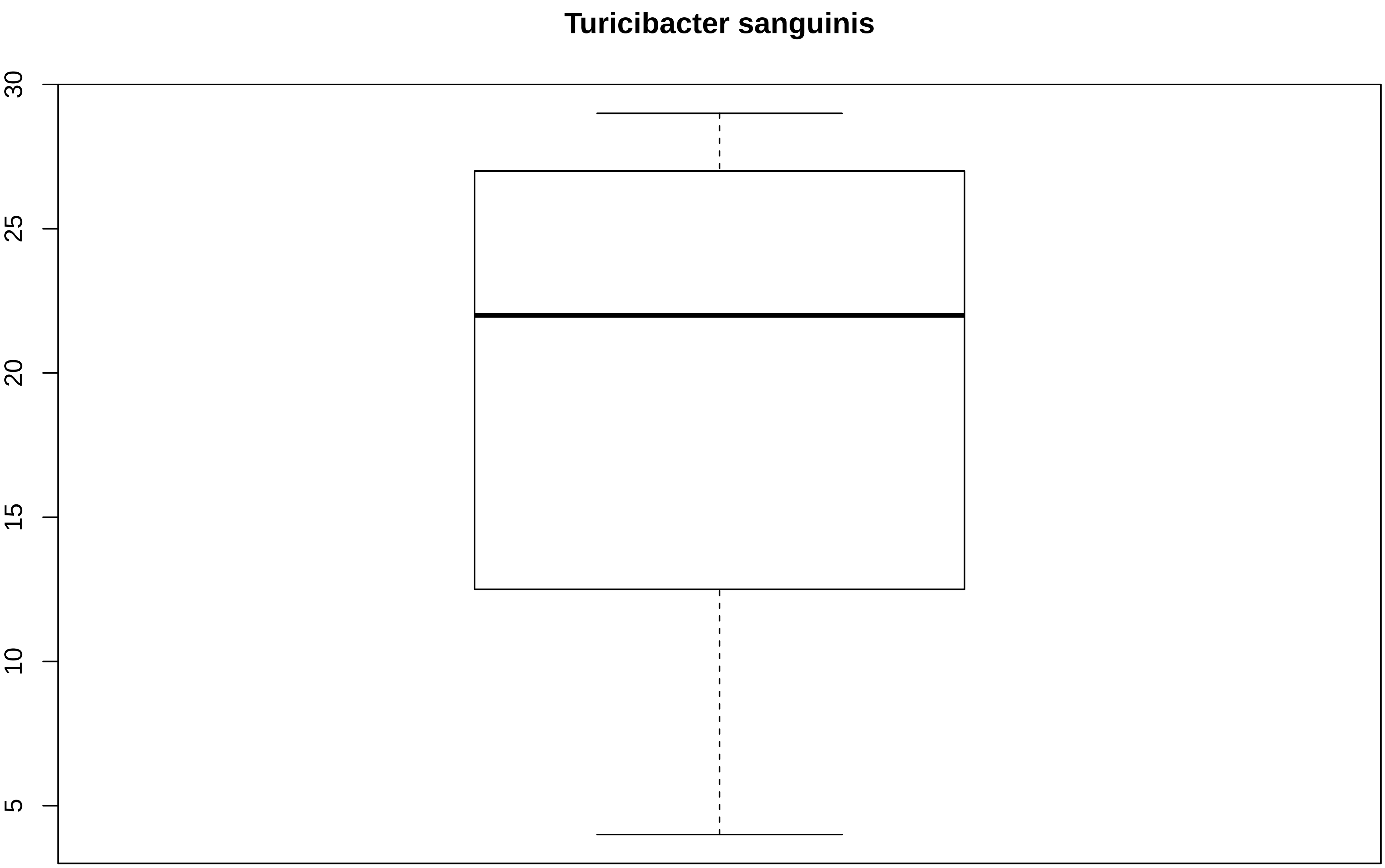
Turicibacter sanguinis
30
25
20
15
10
5

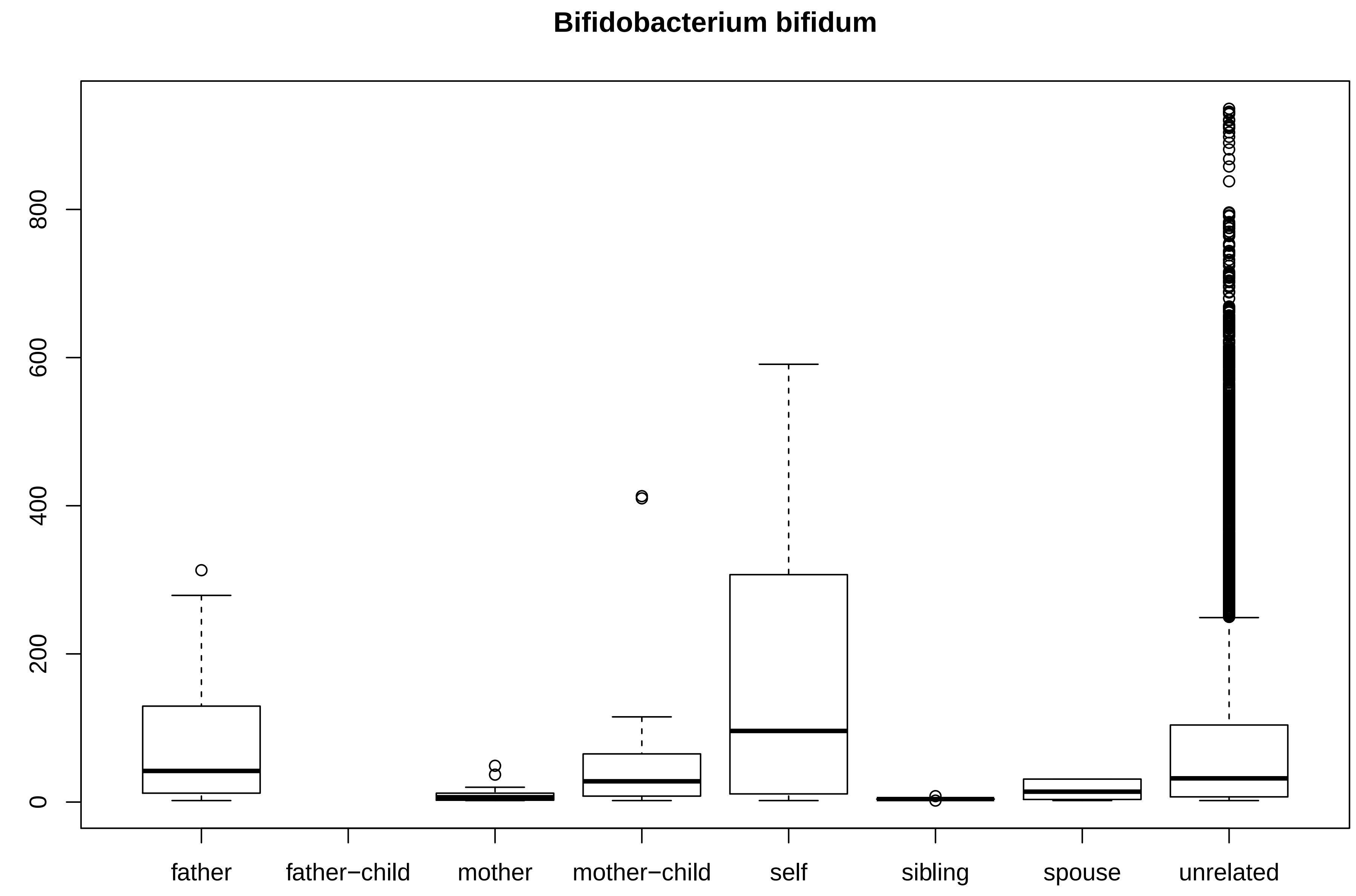
Bifidobacterium bifidum
0
200
400
600
800
father
father−child
mother
mother−child
self
sibling
spouse
unrelated

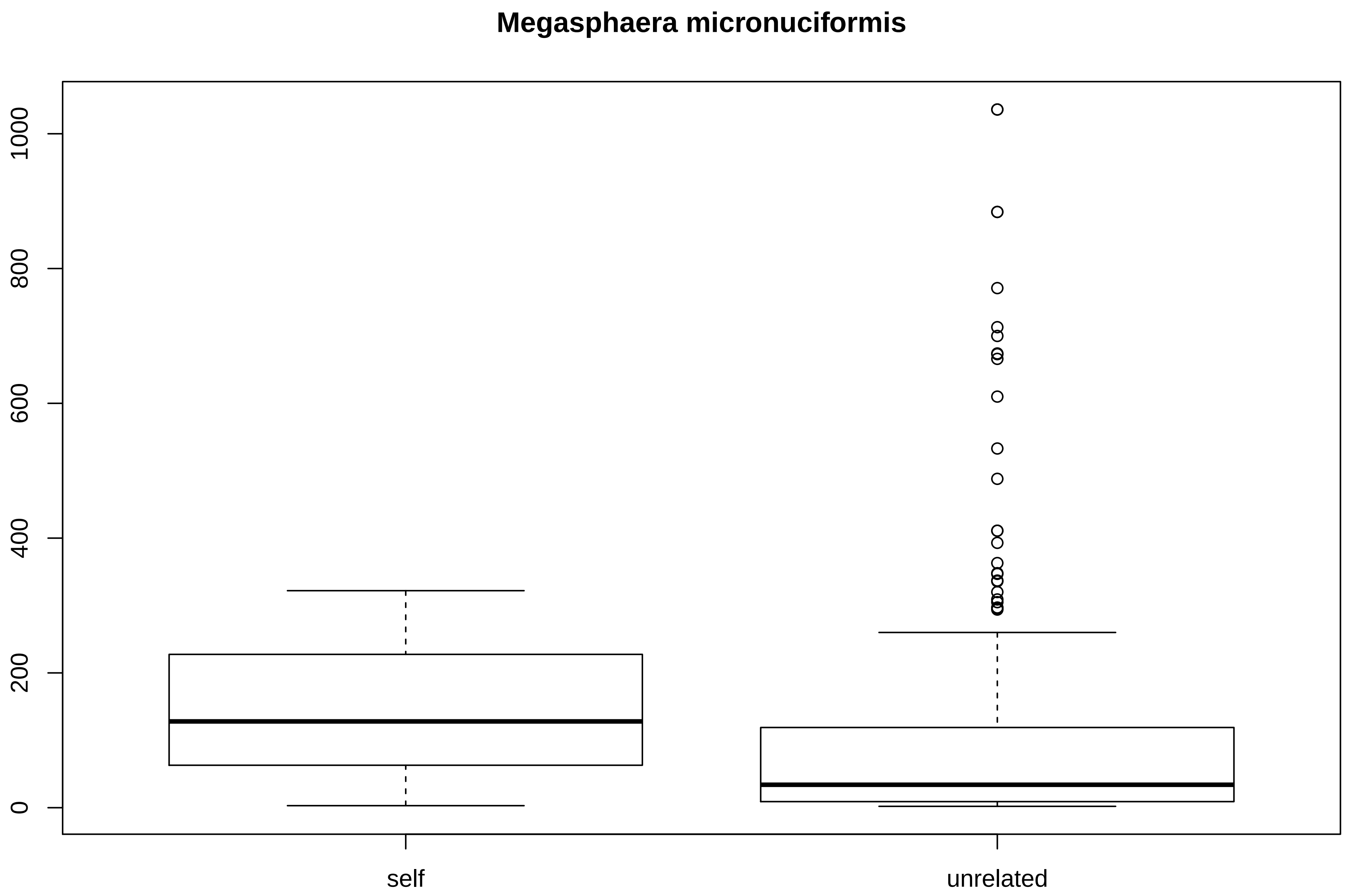
Megasphaera micronuciformis
0
200
400
600
800
1000
self
unrelated

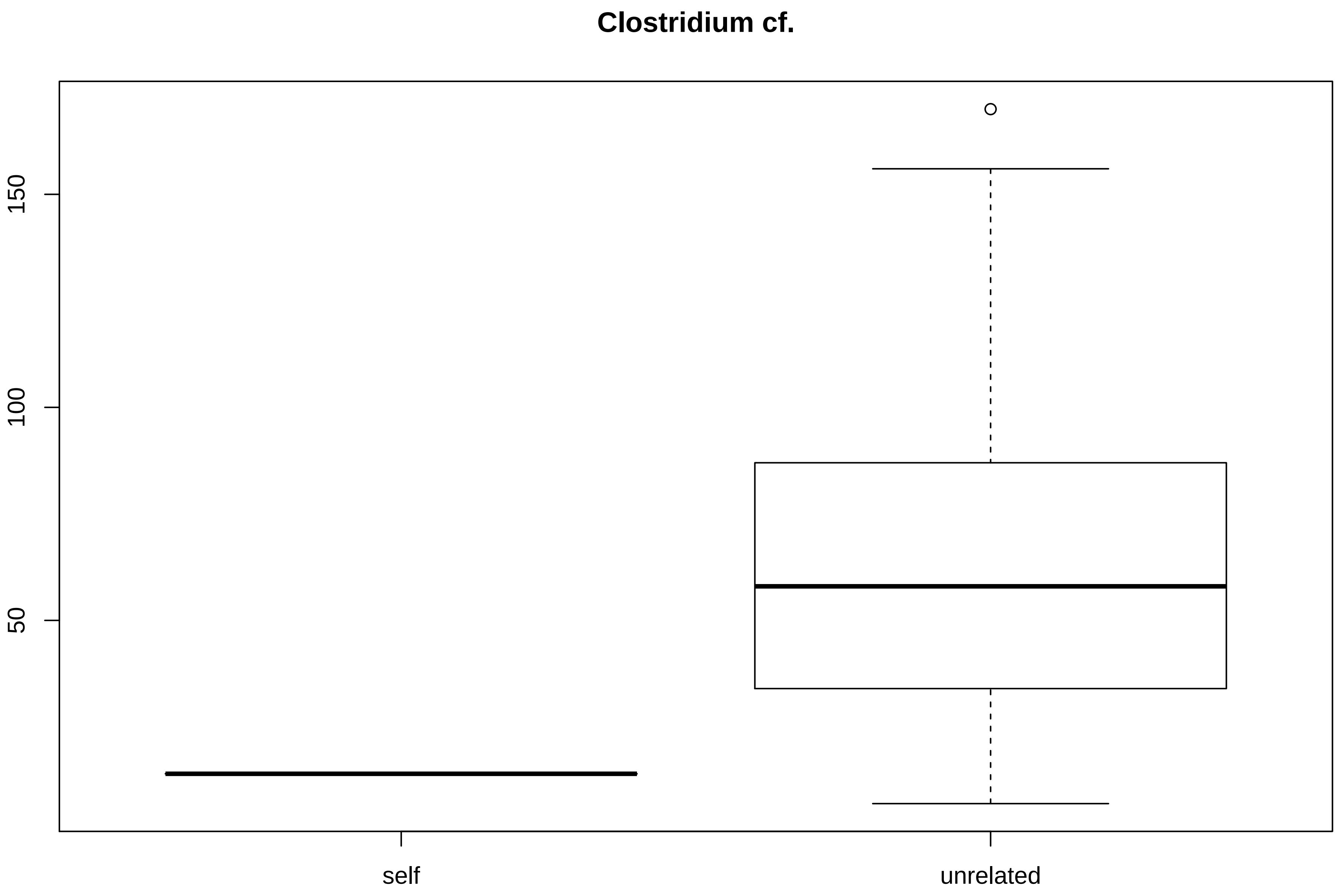

Clostridium cf.
150
100
50
self
unrelated

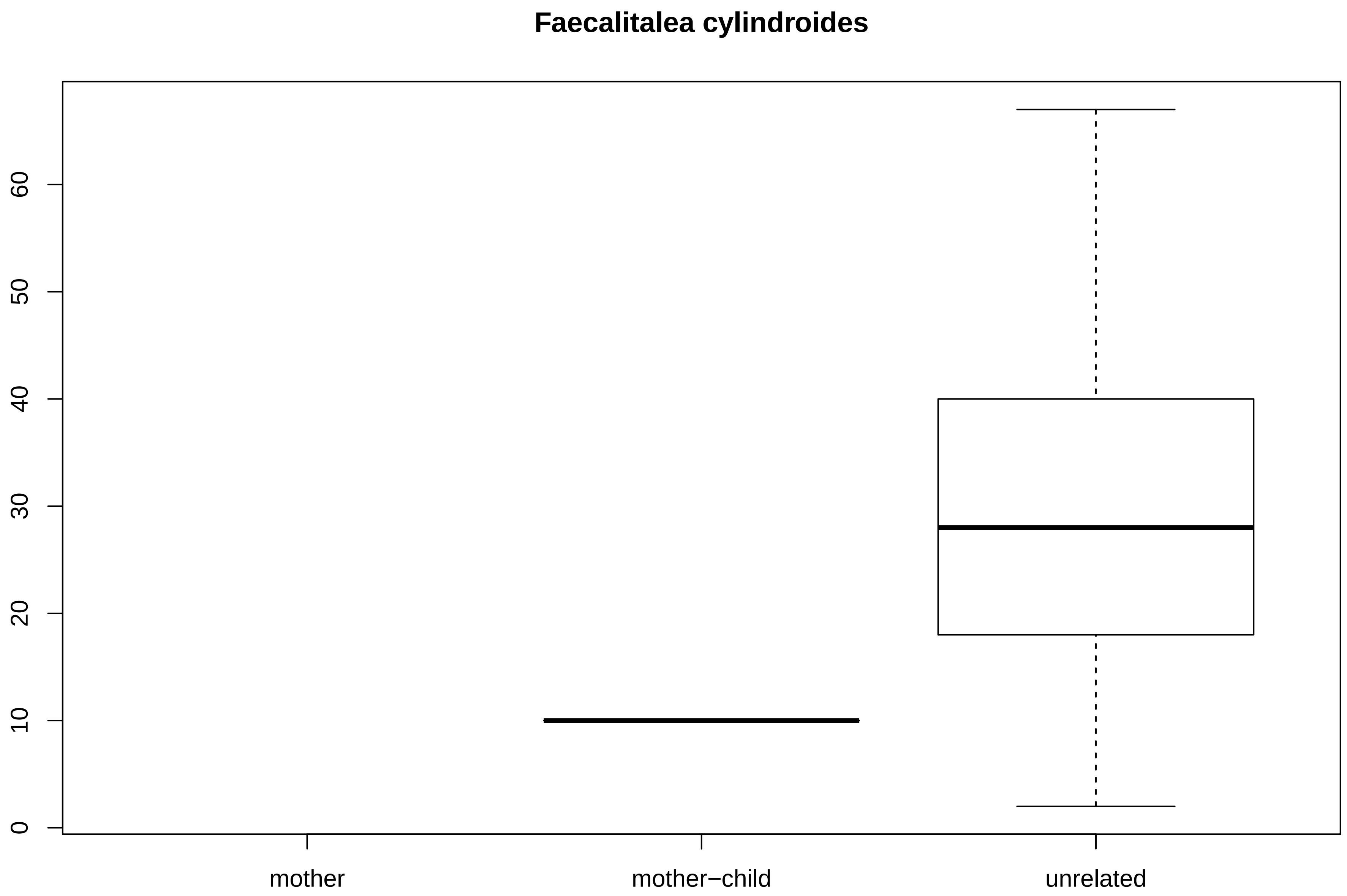
Faecalitalea cylindroides
0
10
20
30
40
50
60
mother
mother−child
unrelated

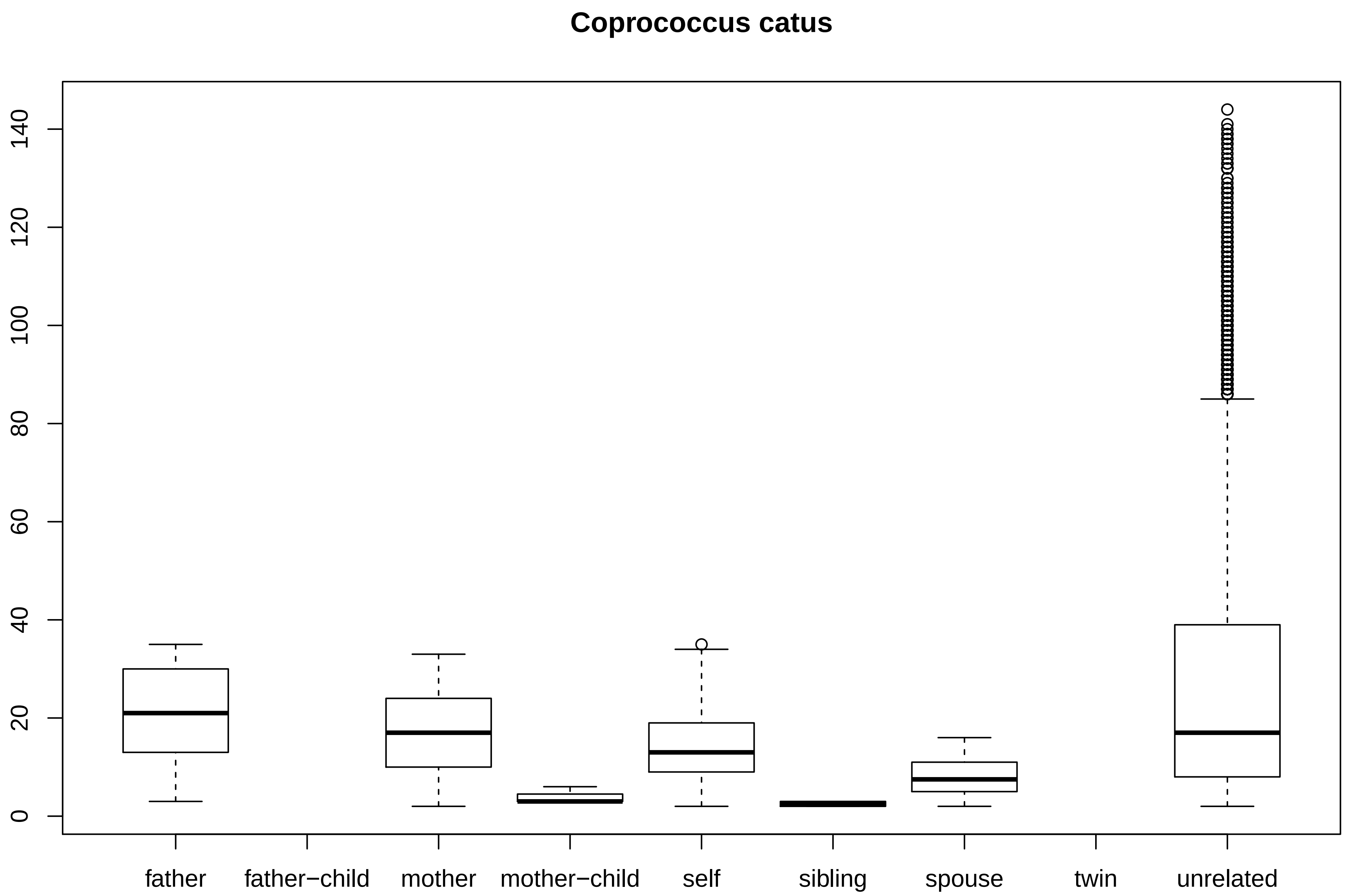

Coprococcus catus
0
20
40
60
80
100
120
140
father
father−child
mother
mother−child
self
sibling
spouse
twin
unrelated

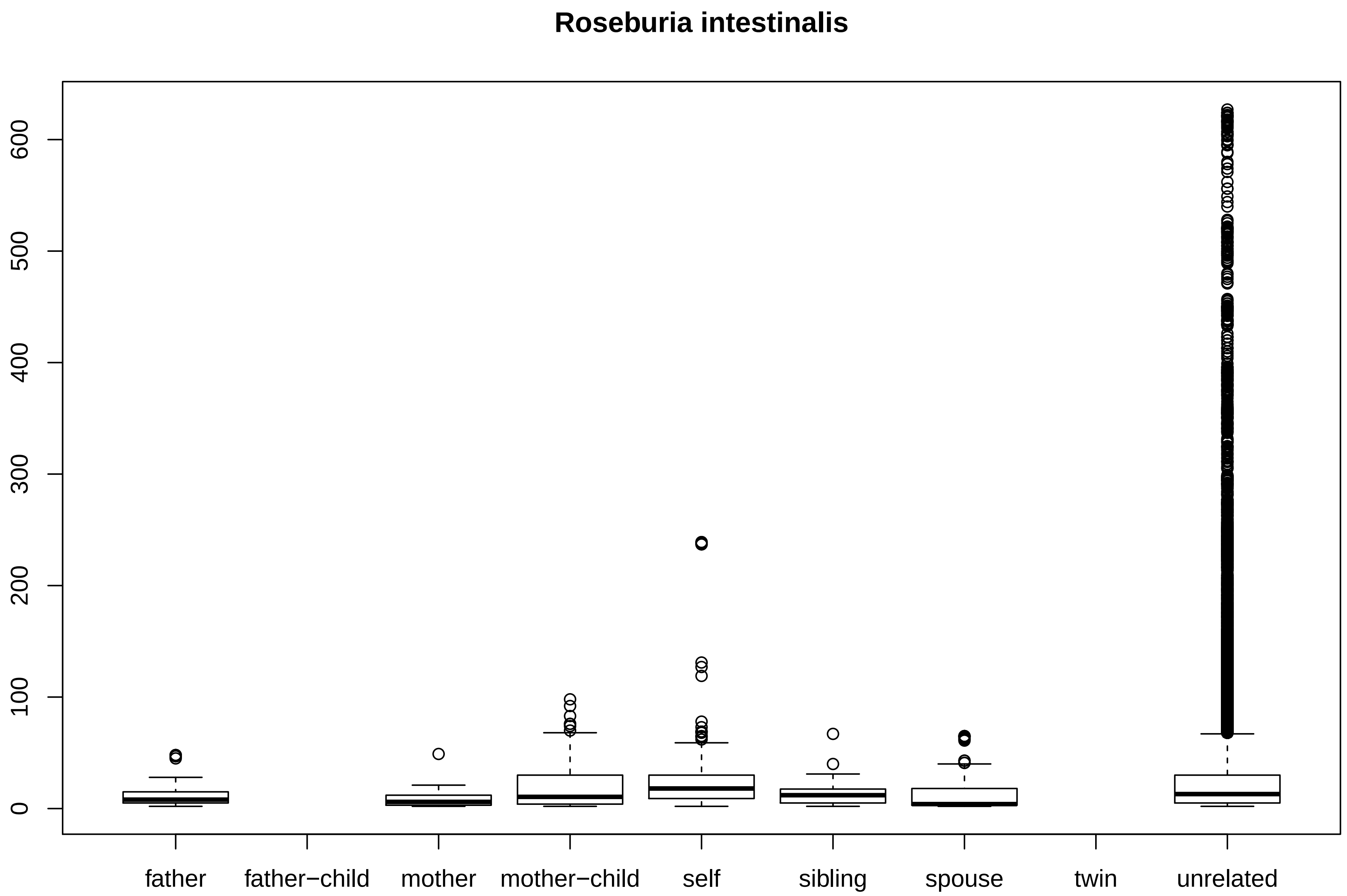
Roseburia intestinalis
0
100
200
300
400
500
600
father
father−child
mother
mother−child
self
sibling
spouse
twin
unrelated

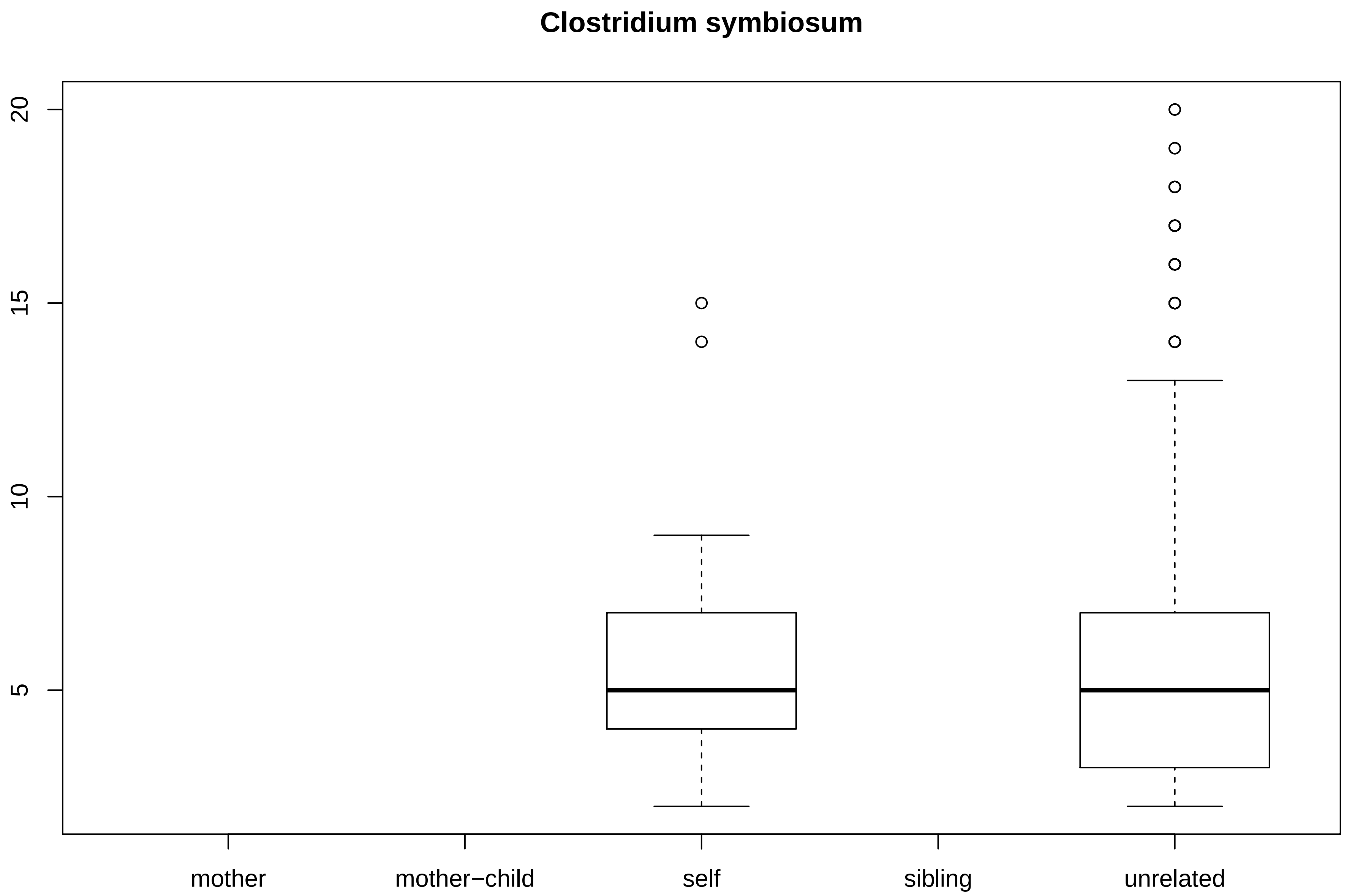
Clostridium symbiosum
20
15
10
5
mother
mother−child
self
sibling
unrelated

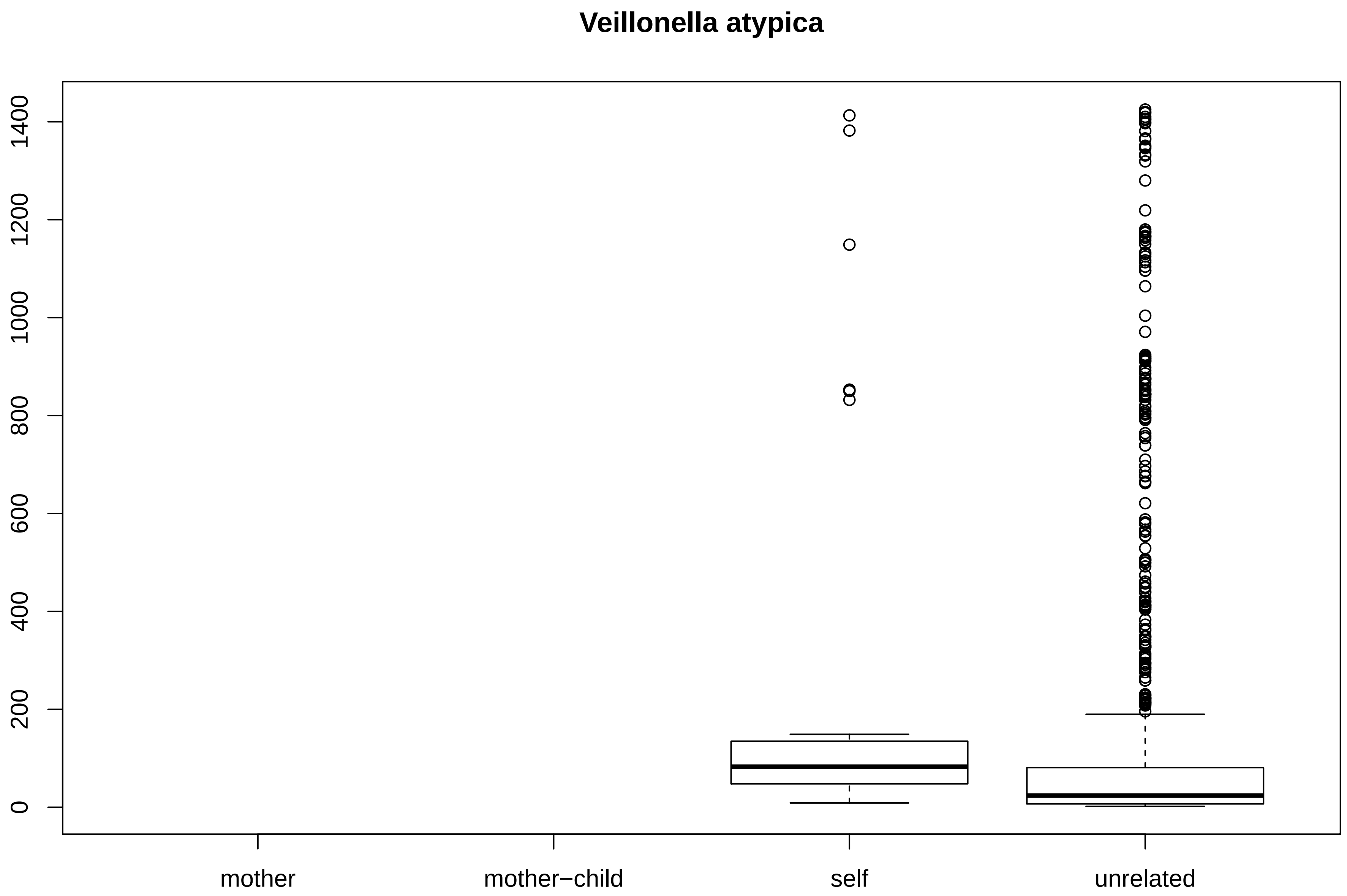
Veillonella atypica
0
200
400
600
800
1000
1200
1400
mother
mother−child
self
unrelated

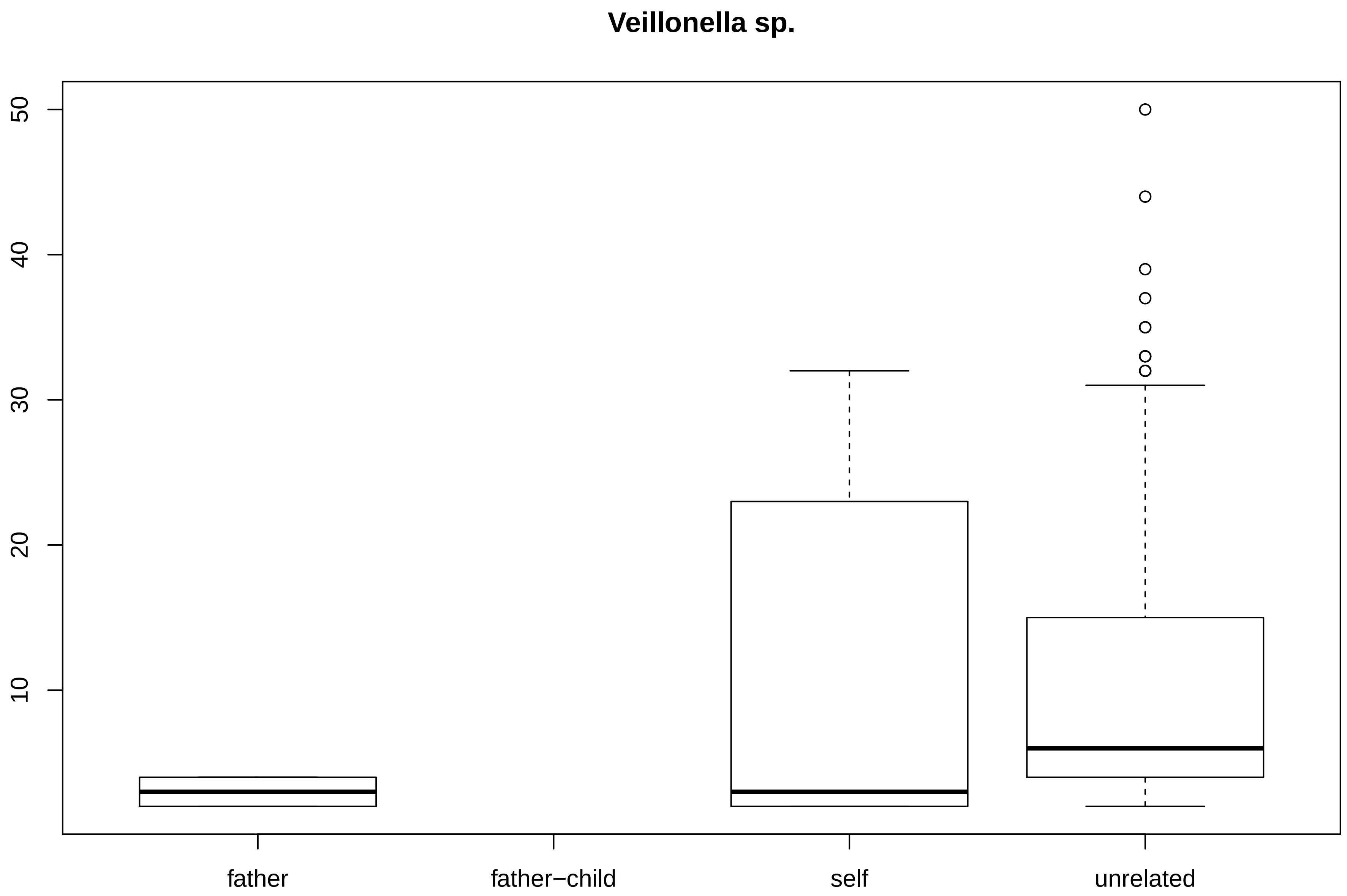
Veillonella sp.
50
40
30
20
10
father
father−child
self
unrelated
