# Supplementary material for: Selective maternal seeding and environment shape the human gut microbiome
Source: Genome Res. 2018 Apr;28(4):561–8. doi: 10.1101/gr.233940.117 (PMC5880245; doi:10.1101/gr.233940.117)
Supplement: Supplemental Material [file supp_gr.233940.117_Supplemental_Table_S1.docx]

Supplemental Table S1. Number of samples/individuals per age group, stratified by birth mode and feeding type. NA = information not available. V= vaginal birth; C = Caesarean birth.

| Birth | Feeding | <1wk | 6mo | 12mo | 2-10yr | 10-25yr | >25yr |
| --- | --- | --- | --- | --- | --- | --- | --- |
| NA | Solid | 0/0 | 0/0 | 0/0 | 0/0 | 114/60 | 407/232 |
| V | Breastfeeding | 70/69 | 65/60 | 8/2 | 0/0 | 0/0 | 0/0 |
| V | Mixed | 15/15 | 43/40 | 0/0 | 0/0 | 0/0 | 0/0 |
| V | Formula | 0/0 | 12/12 | 0/0 | 0/0 | 0/0 | 0/0 |
| V | Solid food | 0/0 | 0/0 | 75/74 | 22/6 | 0/0 | 0/0 |
| V | NA | 12/12 | 4/4 | 14/14 | 0/0 | 0/0 | 0/0 |
| C | Breastfeeding | 8/8 | 7/7 | 0/0 | 0/0 | 0/0 | 0/0 |
| C | Mixed | 6/6 | 13/13 | 0/0 | 0/0 | 0/0 | 0/0 |
| C | Formula | 0/0 | 3/3 | 0/0 | 0/0 | 0/0 | 0/0 |
| C | Solid food | 0/0 | 0/0 | 12/12 | 18/6 | 0/0 | 0/0 |
| C | NA | 3/3 | 0/0 | 3/3 | 0/0 | 0/0 | 0/0 |
|  | Total | 114/113 | 147/139 | 112/105 | 40/12 | 114/60 | 407/232 |
