# Supplementary material for: Selective maternal seeding and environment shape the human gut microbiome
Source: Genome Res. 2018 Apr;28(4):561–8. doi: 10.1101/gr.233940.117 (PMC5880245; doi:10.1101/gr.233940.117)
Supplement: Supplemental Material [file supp_gr.233940.117_Supplemental_Table_S2.docx]

Supplemental Table S2. Number of samples/time span (days) in the unpublished family data sets from the Netherlands (NL) and Germany (GE).

| Family | Father | Mother | Sibling1 | Sibling2 | Twin1 | Twin2 |
| --- | --- | --- | --- | --- | --- | --- |
| NL1 | 3/28 | 3/28 |  |  | 3/28 | 3/28 |
| NL2 | 3/28 | 3/28 | 3/28 |  | 3/28 | 3/28 |
| GE1 | 26/1362 | 7/754 | 8/735 | 2/725 |  |  |
| GE2 | 22/1375 | 9/250 | 8/189 | 6/213 |  |  |
| GE3 | 3/30 | 3/30 | 3/30 |  | 3/30 | 3/30 |
| GE4 | 3/30 | 3/30 | 3/30 |  | 3/30 | 3/30 |
| GE5 | 11/252 | 7/252 | 8/252 | 8/252 |  |  |
| GE6 | 11/918 | 1/1 | 1/1 | 1/1 |  |  |
| GE7 | 3/30 | 3/30 |  |  | 3/30 | 3/30 |
| GE8 | 1/1 | 4/748 | 1/1 | 1/1 |  |  |
