# Supplementary material for: Selective maternal seeding and environment shape the human gut microbiome
Source: Genome Res. 2018 Apr;28(4):561–8. doi: 10.1101/gr.233940.117 (PMC5880245; doi:10.1101/gr.233940.117)
Supplement: Supplemental Material [file supp_gr.233940.117_Supplemental_Table_S3.docx]

| Relation | N pairs |
| --- | --- |
| Father-child | 21 |
| Mother-child | 107 |
| Self | 201 |
| Fraternal sibling | 9 |
| Spouse | 10 |
| Twin | 4 |
| Unrelated | 329 |

**Supplemental Table S3**. Number of compared pairs of individuals by relation.
