# Supplementary material for: Selective maternal seeding and environment shape the human gut microbiome
Source: Genome Res. 2018 Apr;28(4):561–8. doi: 10.1101/gr.233940.117 (PMC5880245; doi:10.1101/gr.233940.117)
Supplement: Supplemental Material [file supp_gr.233940.117_Supplemental_Table_S4.docx]

**Supplemental Table S4**. Number of neonates with maternal (>20% SNV similarity to mother) or non-maternal (<20% similarity) strains, stratified by species.

| Species | Class | Maternal | Non-maternal |
| --- | --- | --- | --- |
| *Bifidobacterium adolescentis* | Actinobacteria | 23 | 0 |
| *Bifidobacterium bifidum* | Actinobacteria | 13 | 0 |
| *Bifidobacterium breve* | Actinobacteria | 1 | 1 |
| *Bifidobacterium longum* | Actinobacteria | 5 | 1 |
| *Bifidobacterium pseudocatenulatum* | Actinobacteria | 9 | 0 |
| *Alistipes shahii* | Bacteroidia | 1 | 0 |
| *Bacteroides caccae* | Bacteroidia | 8 | 1 |
| *Bacteroides eggerthii* | Bacteroidia | 3 | 0 |
| *Bacteroides finegoldii* | Bacteroidia | 1 | 0 |
| *Bacteroides fragilis* | Bacteroidia | 13 | 1 |
| *Bacteroides stercoris* | Bacteroidia | 6 | 0 |
| *Prevotella copri* | Bacteroidia | 1 | 2 |
| *Blautia obeum* | Clostridia | 0 | 1 |
| *Butyrivibrio crossotus* | Clostridia | 0 | 1 |
| *Clostridium sp.* | Clostridia | 0 | 1 |
| *Coprobacillus sp.* | Clostridia | 0 | 1 |
| *Coprococcus catus* | Clostridia | 0 | 2 |
| *Coprococcus eutactus* | Clostridia | 0 | 1 |
| *Dorea longicatena* | Clostridia | 0 | 2 |
| *Erysipelotrichaceae bacterium* | Clostridia | 0 | 1 |
| *Eubacterium hallii* | Clostridia | 0 | 1 |
| *Eubacterium ventriosum* | Clostridia | 0 | 1 |
| *Ruminococcus lactaris* | Clostridia | 0 | 1 |
| *Ruminococcus torques* | Clostridia | 0 | 3 |
| *Turicibacter sanguinis* | Clostridia | 0 | 1 |
| *Dialister invisus* | Negativicutes | 1 | 10 |
| *Collinsella aerofaciens* | Coriobacteria | 1 | 0 |
| *Akkermansia muciniphila* | Verrucomicrobia | 0 | 5 |
