# Supplementary material for: Selective maternal seeding and environment shape the human gut microbiome
Source: Genome Res. 2018 Apr;28(4):561–8. doi: 10.1101/gr.233940.117 (PMC5880245; doi:10.1101/gr.233940.117)
Supplement: Supplemental Material [file supp_gr.233940.117_Supplemental_Fig_S2.pdf]

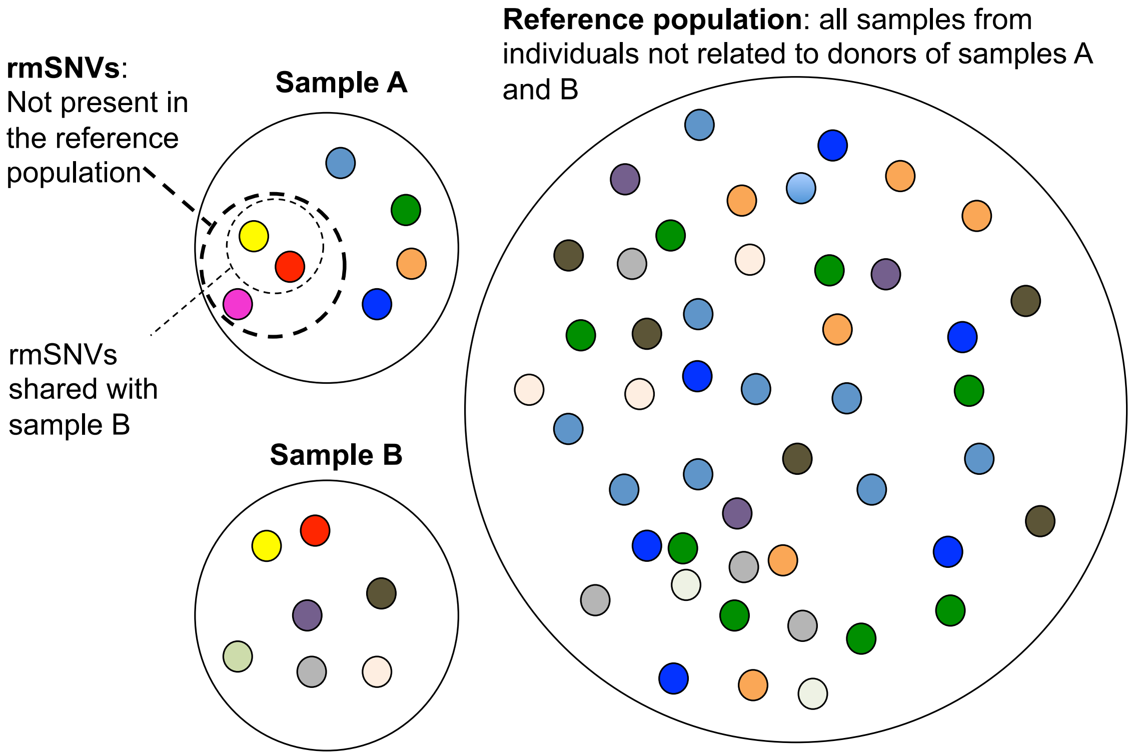

Supplemental Figure S2. Definition of rmSNVs.
For each compared sample pair (here sample A and sample B), we first define a reference population. The reference population contains all other samples that are not derived from the donors of samples A or B, or from their family members. The reference population is thus different for each compared pair of samples. For sample A, the rmSNVs are defined as the SNVs that are not present in the reference population. This set of rmSNVs are then searched for in the SNVs of sample B. In this example, 2/3 rmSNVs are found in sample B, which would mean that sample A shares ca. 67% of the rmSNVs with sample B. Each sample functions as “sample A” and as “sample B” in all pairwise comparisons.
